# Performance indicators for evaluating and optimising hospital services during COVID-19: a scoping review

**DOI:** 10.1136/bmjopen-2026-122362

**Published:** 2026-09-28

**Authors:** Anna Dankers, Dilek Yildirim, Sander Dijkstra, Lem Ngongalah, Máire A Connolly, Jim Duggan, Anne Zander, Tessa van Loenen, Richard J Boucherie, Aura Timen

**Affiliations:** 1 Center for Healthcare Operations Improvement and Research, University of Twente, Enschede, The Netherlands; 2 Primary and Community Care, Radboud University Medical Centre, Nijmegen, The Netherlands; 3 School of Health Sciences, University of Galway, Galway, Ireland; 4 School of Medicine, College of Medicine, Nursing and Health Sciences, University of Galway, Galway, Ireland; 5 Computer Science, University of Galway, Galway, Ireland; 6 Athena Institute, VU University Amsterdam, Amsterdam, The Netherlands

**Keywords:** COVID-19, Quality in health care, Hospitals, Review

## Abstract

**Abstract:**

**Objectives:**

To identify which logistical and clinical healthcare key performance indicators (KPIs) were applied in hospitals during the COVID-19 pandemic and how frequently they were used when evaluating and optimising hospital care services.

**Design:**

Scoping review reported in accordance with the Preferred Reporting Items for Systematic Reviews and Meta-Analyses extension for Scoping Reviews (PRISMA-ScR) framework.

**Data sources:**

The search was performed in PubMed, CINAHL, EMBASE, Scopus and Web of Science and included articles published from the beginning of the COVID-19 pandemic, that is, 1 January 2020, until 11 November 2024, the date of the final search. The search strategy combined terms related to (1) COVID-19/pandemic, (2) performance and (3) hospital care.

**Eligibility criteria:**

Eligible studies were peer-reviewed, English-language publications that reported measurable performance indicators evaluating or optimising hospital care services during the COVID-19 pandemic. Studies had to explicitly define or evaluate KPIs and focus on inpatient hospital settings (≥1 overnight stay) in countries within the European Union, the European Economic Area, the Schengen area or the UK. Multicountry or global studies were included when they contained relevant European data or when the geographical context was not determinant (eg, modelling studies). Exclusion criteria included non-peer-reviewed publications, conference abstracts, organisational reports, literature reviews, studies without original results, studies without accessible full text and non-English publications.

**Data extraction and synthesis:**

Titles, abstracts and full texts were screened independently by three reviewers. Disagreements were resolved through discussion and consultation with two additional reviewers. Relevant study characteristics and KPI-related information were extracted using a predefined data extraction form. KPIs were categorised inductively into thematic categories. Structured data were analysed at both the study and KPI-category level. Data without a predefined structure, such as KPI definitions and key findings, were analysed manually at the KPI-category level.

**Results:**

Across 94 included studies, 459 distinct KPIs were extracted. Given substantial heterogeneity, KPIs were grouped inductively into six main categories: (1) clinical outcomes, (2) resources and capacity, (3) care delivery volumes, (4) care delivery performance, (5) access time and sojourn time and (6) other indicators. KPIs were reported in diverse formats, including volumes, percentages, scores, scales, characteristics and binary outcomes. Identical KPIs were sometimes defined differently across studies, while similar measures were reported under different terminology. The most common reason for KPI use was for the comparison between pre-COVID-19 and COVID-19 periods, followed by supporting optimisation and exploring quality of care during the pandemic. Most studies emphasised either clinical or logistical indicators, with limited integration of both perspectives within a single performance assessment. The majority of KPIs were not pandemic-specific but reflected indicators already used in routine hospital performance monitoring.

**Conclusions:**

Hospital performance during COVID-19 was evaluated using a wide range of heterogeneous KPIs, with substantial variation in definitions and reporting practices. Greater transparency and standardisation in KPI definitions may improve comparability, reproducibility and preparedness for future health crises. Integrating clinical and logistical performance perspectives may provide a broader view of hospital performance and support decision-making during pandemics.

**STRENGTHS AND LIMITATIONS OF THIS STUDY**

- This scoping review adopted a broad, hospital-wide perspective, mapping key performance indicators (KPIs) across multiple clinical areas rather than focusing on a single specialty.
- Studies from medical, organisational and operations research perspectives were included, allowing hospital performance to be examined from multiple perspectives.
- The multidisciplinary research team, combining clinical and operations research expertise, supported the interpretation and contextualisation of findings across domains.
- The review was restricted to European countries and English-language publications, which may limit representativeness and exclude relevant work from other regions.
- The review focused exclusively on the COVID-19 pandemic, and therefore, the identified KPIs may reflect pandemic-specific characteristics and not fully capture performance aspects relevant to future pandemics with different epidemiological features.

## Introduction

 In January 2020, the WHO declared the outbreak of COVID-19 a Public Health Emergency of International Concern,^1^ signalling the start of a global health crisis that profoundly disrupted healthcare systems.^2 3^ Hospitals were confronted with sudden surges of patients infected with the SARS-CoV-2 virus while also striving to maintain essential care for non-infected patients.^4 5^ Together with infection prevention and control measures, workforce shortages and the reallocation of resources, this dual challenge of managing a rapid influx of infectious patients while ensuring continuity of non-infectious patient care created unprecedented pressure on delivery, efficiency and quality of care.^6–9^ These combined pressures emphasised the importance of systematic performance measurement to monitor hospital functioning and to sustain both continuity and quality of care for all patients.

Evaluating hospital service performance involves understanding how effectively and efficiently both logistical processes and clinical care are delivered. Logistical key performance indicators (KPIs) refer to indicators that capture the organisation and coordination of hospital resources and processes that support care delivery. These include metrics related to capacity utilisation, patient flow, staffing availability and resource allocation within the hospital system. While the term logistical KPIs in some healthcare literature may specifically refer to supply chain performance (eg, procurement, inventory management and storage efficiency), in our context of hospital service delivery they are used more broadly to describe operational aspects that enable the delivery of care, such as patient throughput and length of stay. In contrast, clinical care KPIs refer to indicators that assess the quality, safety and outcomes of patient care. These include measures such as treatment timeliness, adherence to clinical guidelines or care pathways, complication rates and patient outcomes. Together, these two perspectives provide a comprehensive view of hospital functioning, linking the organisational and operational structure of healthcare delivery to (the resulting quality of) clinical practice. During the COVID-19 pandemic, monitoring both dimensions became essential as hospitals were required to continuously adjust their internal logistics to adapt hospital processes to surges in demand but also to maintain access to and quality of essential services for all patient groups (both COVID-19 and non-pandemic care patients).^10 11^

Several reviews have assessed how healthcare systems responded to the COVID-19 pandemic. Moynihan *et al*^12^ performed a systematic review of studies reporting on pandemic-related changes in overall healthcare utilisation, where they focus on quantifying volume changes (eg, visits, admissions, diagnostics) during the COVID-19 pandemic; whereas Fleming *et al*^13^ examine how the resilience of health systems has been measured across various health system crises. This latter review focuses on the relation between indicators and the phase of the crisis. Within a hospital, that is, concentrating on KPIs related to hospital (care) services instead of system-wide healthcare, we observed that literature reviews during the COVID-19 pandemic are available at the clinical condition-level, for example, ST-elevation myocardial infarction care^14^ (emergency treatment for a severe heart attack), cancer care^15^ and cardiac care.^16^ Furthermore, we encountered reviews that outline performance indicators for the emergency department (ED)^17^ and for the acute care pathway.^18^ However, none of these reviews integrate both operational/logistical KPIs and clinical outcome indicators from a hospital-wide perspective.

This scoping review identifies which logistical and clinical healthcare performance indicators were applied in hospitals during the COVID-19 pandemic, and how frequently they were used (across studies), when evaluating and optimising hospital (care) services. This paper provides the reader with a comprehensive and categorised collection of indicators that have been used in different performance analysis studies, specifically during the COVID-19 pandemic. This review may serve as a reference for researchers, hospital managers and health system planners when selecting or developing performance indicators to evaluate hospital services during future pandemics and other health emergencies.

## Methods

### Study design

We conducted a scoping review to obtain an overview of how hospital performance has been assessed in the literature during COVID-19. We opted for a scoping review because the integrated assessment of hospital performance from both logistical and clinical perspectives, during the COVID-19 pandemic, is broad and, to the best of our knowledge, has not been synthesised in existing reviews that categorise both indicator types at a hospital-wide level. Given our exploratory aim to map and categorise hospital performance indicators, including how they were defined and applied across studies, we used a scoping review approach.^19^ This approach was well suited to identifying which logistical and clinical KPIs were used and highlighting gaps in the evidence. The eligibility criteria, search strategy (including search string), databases to be searched and screening procedures were determined a priori before conducting the review. The screening process was conducted by three reviewers, with two additional reviewers available for consultation and conflict resolution. No review protocol was prospectively registered or made publicly available. The findings of this review can inform the development of hospital quality assessment frameworks for future pandemics. Reporting followed the Preferred Reporting Items for Systematic Reviews and Meta-Analyses extension for Scoping Reviews (PRISMA-ScR) checklist.^20^ In line with the scoping review approach and our objective to map and characterise hospital performance indicators, we did not undertake a formal critical appraisal.

### Information sources and search strategy

The literature search was performed in PubMed, CINAHL, EMBASE, Scopus and Web of Science and included articles published from the beginning of the COVID-19 pandemic, that is, 1 January 2020, until 11 November 2024, the date of the final search. The search strategy was developed in collaboration with an experienced medical information specialist. Search terms were structured around three main concepts: (1) COVID-19/pandemic, (2) performance and (3) hospital care. Pilot searches informed refinements of the strategy. For each database, the complete search string is provided in online supplemental appendix A. All identified references were exported to EndNote^21^ for initial deduplication. These were then imported into Covidence,^22^ where a second automated deduplication was performed.

### Eligibility criteria

We developed explicit eligibility criteria aligned with our research question. The following inclusion criteria were set: (1) studies reporting data from countries within the European Union, the European Economic Area, the Schengen area or from the UK; multicountry or global studies were included when they contained relevant European data, or when the country of conduct was not a determining factor (eg, operations research, mathematical models); (2) peer-reviewed publications; (3) studies published in English; (4) studies reporting data from hospitals providing inpatient care (≥1 overnight stay), with outpatient facilities eligible only if directly affiliated with hospitals through shared management or resources; hospital support services (eg, laboratories, pharmacies) were eligible if hospital-operated, as were studies of simulated pandemic settings (eg, drills); (5) studies reporting quantitative measures or frameworks for evaluating or optimising hospital care services with indicators explicitly defined or evaluated; and (6) studies focusing on the COVID-19 pandemic. Studies were excluded if they did not provide measurable performance indicators on hospital care services during the COVID-19 pandemic. In addition, the following were excluded: (1) non-peer-reviewed publications, conference abstracts, organisational reports or country-level annual health assessments; (2) literature reviews or other types of studies that do not provide original results; and (3) studies for which the full text was not available through online access, university library searches or after contacting the authors.

### Study selection and data extraction

Title and abstract screening were performed in Covidence.^22^ To ensure consistency, for the first 150 papers, the title and abstract were screened in parallel by three reviewers (AD, SD and DY), independently and blinded to each other’s decisions. After reaching agreement (88%), the remaining records were divided among the three researchers and screened independently. Full-text screening was first conducted in blind mode by three reviewers (AD, SD and DY), by screening 50 articles (10%). After disagreements were resolved through discussion, the screening was conducted independently. If necessary, conflicts were referred to a fourth and fifth reviewer for consultation (TvL and AZ). The reasons for exclusion were documented in Covidence.

A data extraction form was developed in Airtable^23^ to capture relevant information from included studies. The following variables were extracted: title, authors, year of publication, country, study design (grouped by observational quantitative studies, modelling studies and mixed study designs), hospital type (single or multiple hospitals; categorised as general, specialty or academic, or combinations thereof), medical specialty, patient type (categorised as COVID-19, non-pandemic, mixed or unknown), inpatient or outpatient care, data reference period, KPIs, KPI definitions, methods of measurement, reason for using KPI, key findings on KPI and lessons and recommendations for the use of the KPI. This was piloted with a small set of studies and refined iteratively. Data extraction was divided among three reviewers (AD, SD and DY), and extracted data were subsequently cross-checked for accuracy.

### Data synthesis

To enable structured analysis, the identified KPIs were organised into thematic groups such as resource capacity, patient flow, clinical outcomes, process and quality measures and other categories reflecting distinct aspects of hospital performance. The categorisation of KPIs was conducted inductively, allowing subcategories to emerge from the data itself rather than being predefined. The structured data (eg, bibliographic details such as year and country, study design, hospital type, medical specialty, patient type and methods of measurement) was analysed at both the article and KPI-category level using pandas^24^ in Python. In contrast, data that did not follow a predefined structure, such as definitions and key findings, was not suitable for automated processing and was therefore analysed manually by the reviewers at the KPI-category level.

### Patient and public involvement

None.

## Results

### General characteristics of included articles

The final search yielded 5424 records across the five databases: 1096 from PubMed, 829 from CINAHL, 1678 from Embase, 944 from Scopus and 877 from Web of Science. After initial duplicate removal, 3300 records remained for screening. An additional 88 duplicate records were identified during the screening process and removed before title and abstract screening, resulting in 3212 records for screening. Titles and abstracts were screened for relevance, resulting in 503 papers selected for full-text review. Following the full text screening, 94 studies met the inclusion criteria and were included for data extraction. The PRISMA diagram is given in figure 1. A complete list of the included studies and their general characteristics (eg, year and country) is presented in online supplemental appendix B.

**Figure 1:**
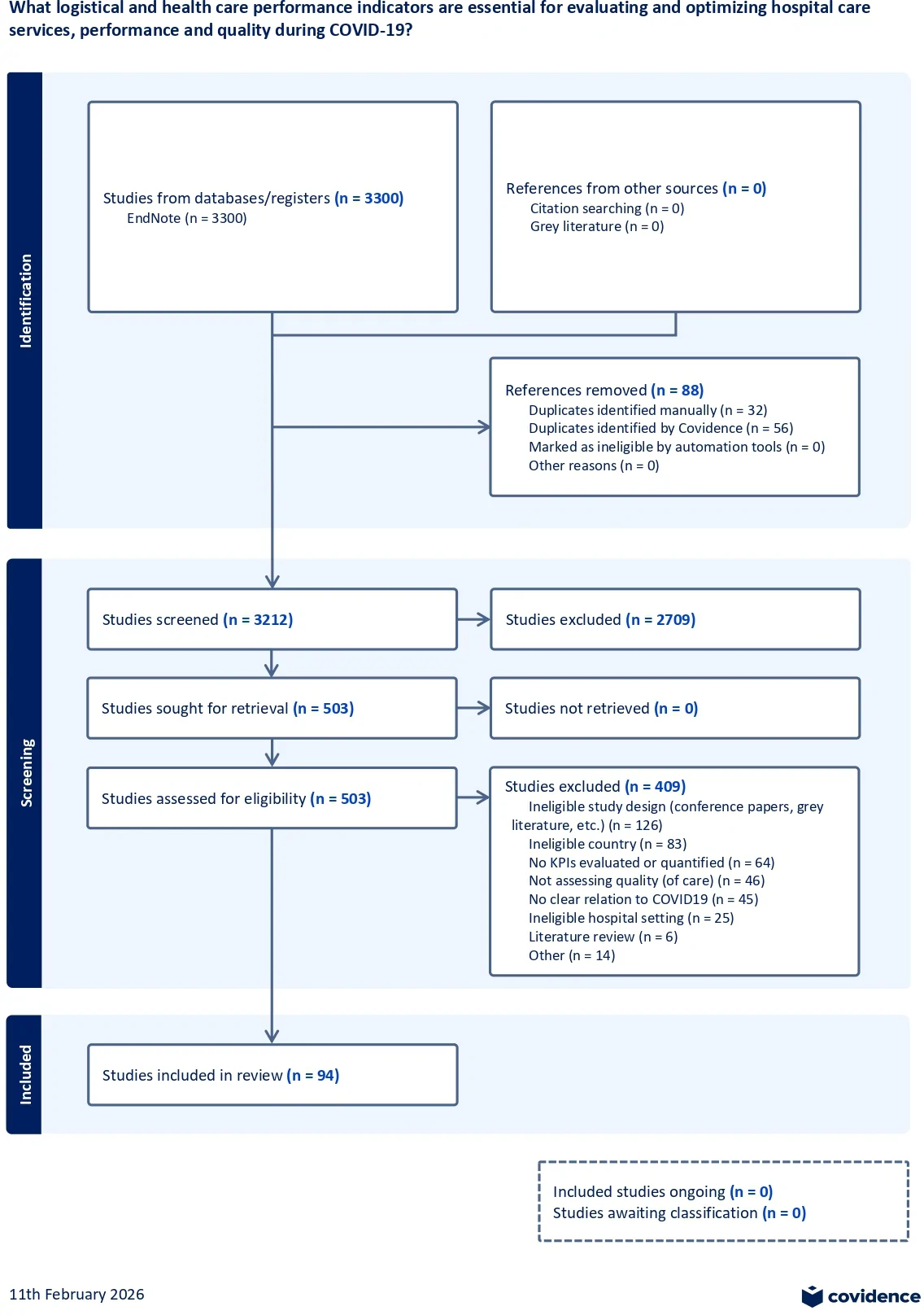
PRISMA diagram. KPIs, key performance indicators; PRISMA, Preferred Reporting Items for Systematic Reviews and Meta-Analyses.

Figure 2 presents the distribution of the 94 articles by country. The literature found was concentrated largely in Southern and Western Europe. A subset of twelve studies involved cross-national designs, for example, collaborative work between the UK and the Netherlands. Of the included articles, 11 were from 2020, 21 from 2021, 33 from 2022, 18 from 2023 and 11 from 2024.

**Figure 2:**
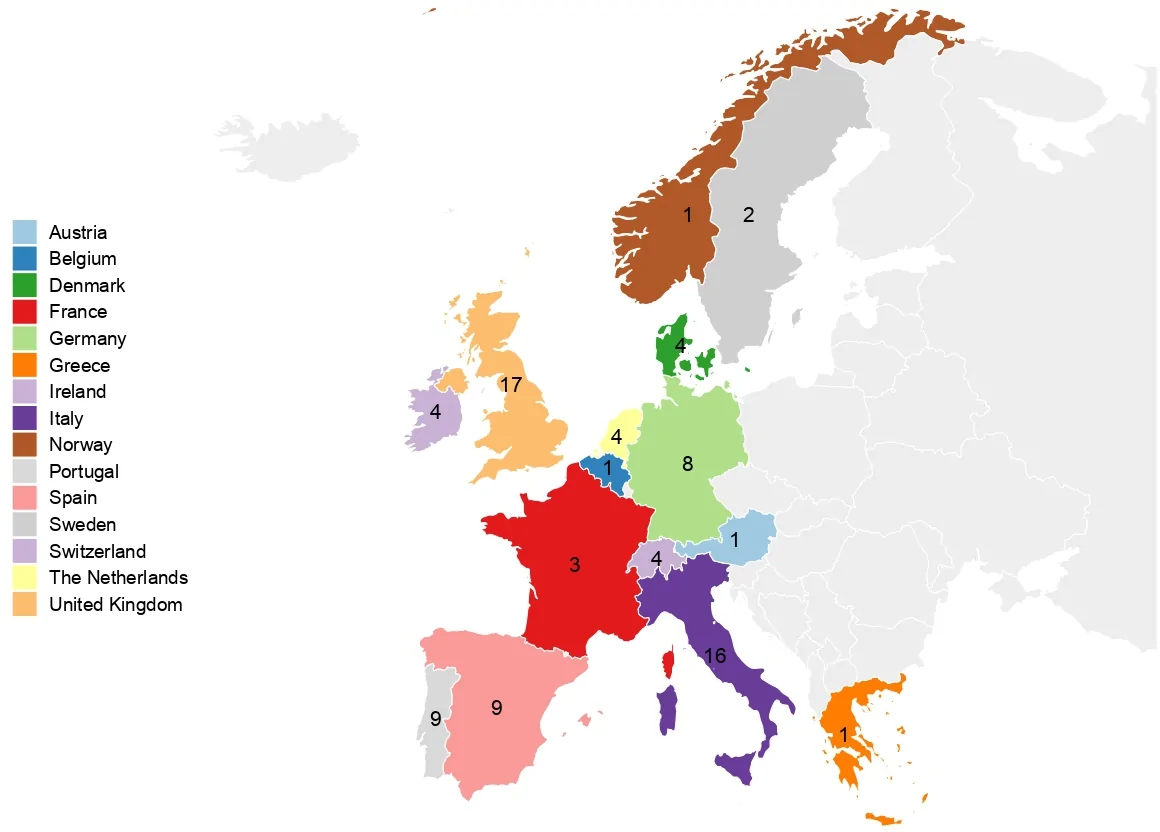
Country of origin of included studies*. *Six studies were conducted in multiple (≥3) countries. **For the studies conducted in two countries, an additional count was added for each country.

Regarding study design, 59 were observational quantitative studies (mostly retrospective), 27 were modelling studies and 8 articles combined modelling work with data obtained from retrospective observational quantitative studies. Of the included articles, 28 involved multiple mixed hospital types, while 21 focused on a single academic hospital. A smaller number of studies addressed single general hospitals (10), single specialty hospitals (5) or single hospitals of unknown type (10). An additional 12 studies involved multiple hospitals of unknown type. Only a few studies examined multiple academic hospitals (3), multiple specialty hospitals (3), or did not specify the hospital type at all (2).

Regarding medical specialty, most articles assessed performance in infectious disease or COVID-19 care (12), multiple specialties or hospital-wide care (11), emergency care (10), followed by orthopaedics or orthopaedic and trauma surgery (10), cardiology (9), neurology/neurosurgery (6), oncology (5), intensive care unit (ICU) (4), gastroenterology (4) and gynaecology/obstetric care (4). A smaller number of studies addressed other specialties, including endocrinology, general surgery, haematology, palliative care and transplantation. Seven articles did not specify a medical specialty.

In terms of patient type, most articles focused on non-pandemic patient care (35) or a combination of COVID-19 and non-pandemic care (24), while 20 articles addressed COVID-19 patient care exclusively. 15 studies did not specify the patient type. Regarding care setting, 34 articles focused on inpatient care, 10 on outpatient care and 15 included a mix of both. 35 articles did not specify whether care was inpatient or outpatient.

The primary reasons reported in the articles for using KPIs were to compare pre-COVID-19 to COVID-19 periods (36 articles), support optimisation of decision-making (19) and explore quality of care during COVID-19 (10). Other reasons, reported less frequently, included evaluating efficiency, quantifying productivity and comparing hospitals or countries, with some studies combining multiple reasons.

For methods of measuring KPIs, most articles obtained their quantitative data from electronic data records (66 articles), with smaller numbers of studies using model calculations (8), questionnaires (9) or simulation outputs (4). A few studies combined methods (6) or used direct observation (1).

### Performance indicators

From 94 articles, we extracted 459 KPIs. Given the heterogeneity of the identified indicators, we adopted an inductive, bottom-up grouping approach rather than applying a predefined classification framework. All KPIs were reviewed iteratively and grouped based on conceptual similarity, that is, the type of information they provided regarding hospital performance and quality of care. KPIs reflecting similar underlying constructs (eg, mortality-related measures, volume of admissions or procedures) were first clustered into smaller, conceptually coherent groups. These groups, later called subcategories, were subsequently organised into six main categories: (1) *clinical outcomes*, covering patient characteristics related to disease and treatment, such as mortality, individual outcomes, readmissions and reoperations, complications; (2) *resources and capacity*, covering KPIs related to the availability and utilisation of (sometimes limited) hospital resources, such as beds, staff and equipment, as well as efficiency-related measures, for example, surgical efficiency; (3) *care delivery volumes*, including KPIs that report on production measures, such as the number of surgeries performed or diagnoses established; (4) *care delivery performance*, covering KPIs on the quality of care delivery, including adherence to treatment and diagnostic guidelines, patient safety and adverse events, as well as the quality of logistical processes and patient satisfaction; (5) *access time and sojourn time*, consisting of all KPIs that measure time-related indicators; and (6) *other*, containing KPIs that do not fit into any of the aforementioned categories. Although some KPIs subcategories could conceptually relate to more than one category, classification was based on their primary informational function.

In figure 3, an overview of the categories and their subcategories, and the number of KPIs found in each subcategory, is presented. A table presenting the full list of KPIs together with their definition, study design and medical specialty can be found in online supplemental appendix C.

**Figure 3:**
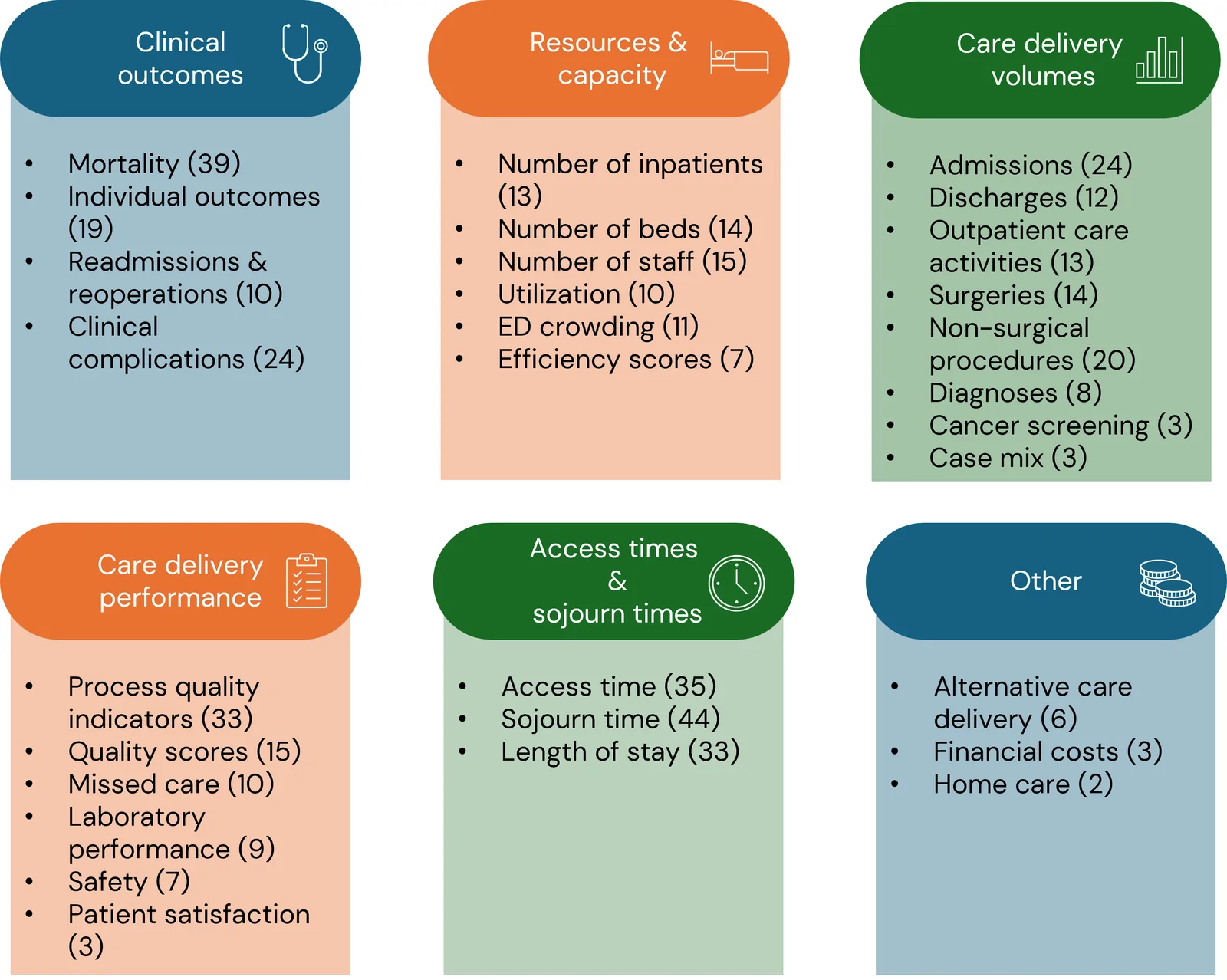
Overview of the six categories and their subcategories (with number of KPIs in that subcategory). ED, emergency department; KPIs, key performance indicators.

Figure 4 presents the frequency of study design and method(s) of measurement at the subcategory level. In the remainder of this subsection, we discuss the identified KPIs, along with their definitions and the clinical specialty within the hospital to which they were applied, in more detail, organised by subcategory.

**Figure 4:**
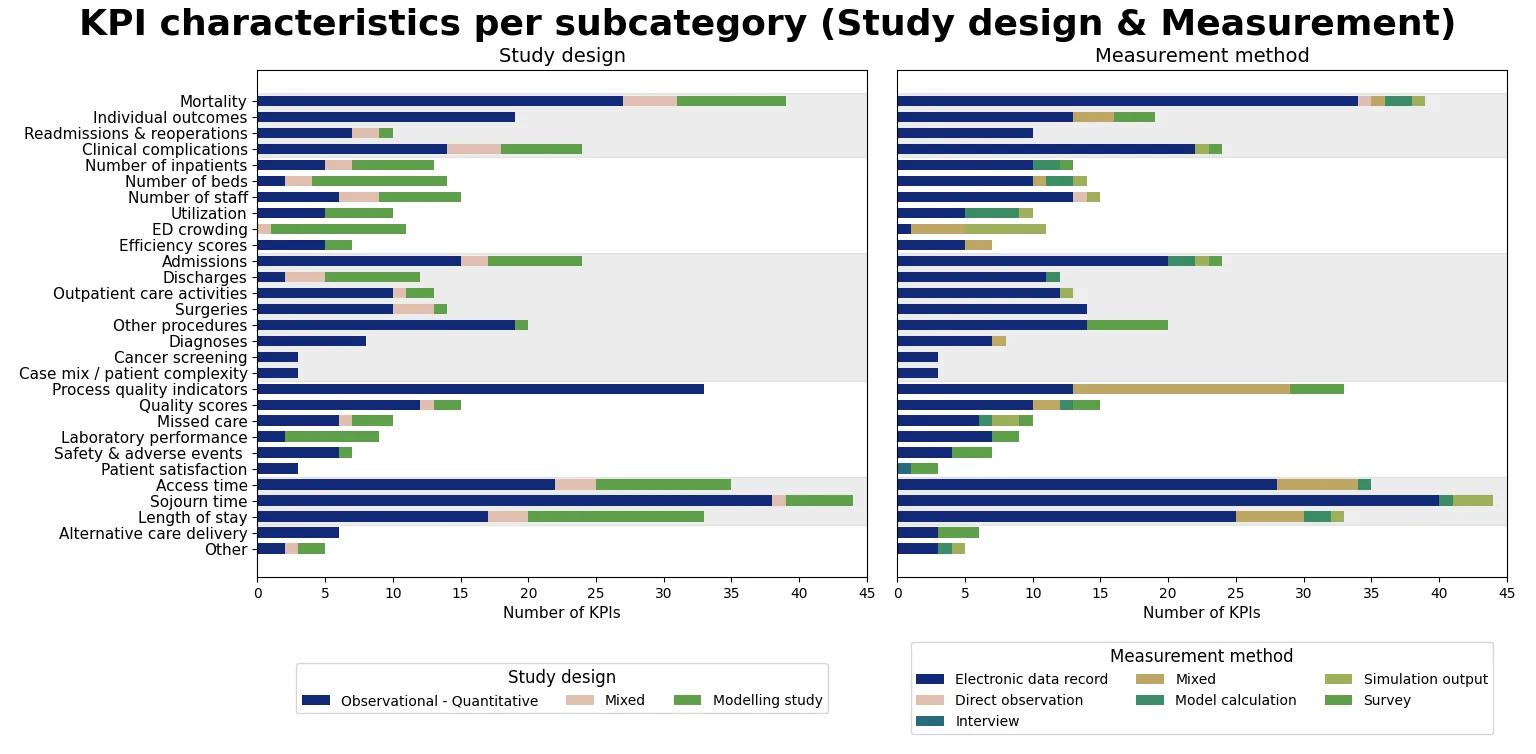
Study design and method of measurement for the 29 subcategories. The grey and white parts in the graphs indicate the six main categories. ED, emergency department; KPIs, key performance indicators.

#### Clinical outcomes

##### Mortality

Across 30 articles, 38 mortality KPIs were identified in 13 specialties, most often in hospital-wide evaluations and in specialties dealing with infectious diseases. Mortality indicators assessed patient survival via time-sensitive outcomes, such as overall or daily mortality,^25–32^ 30-day mortality,^33–37^ in-hospital mortality^25 34 36 38–43^ and, less commonly, 1-year mortality.^27^ Although definitions varied in timeframe, all served as objective and reliably measurable clinical safety endpoints.

In most studies, mortality was reported as counts or rates and was primarily used to evaluate clinical outcomes among patients with COVID-19, particularly regarding the severity and progression of illness.^25–29 31–42 44–52^ Many articles^26 29 31 33 34 36 37 39 40 42 44 45 47 51–53^ compared pre-COVID-19 and pandemic periods, with most reporting increased mortality during COVID-19,^26 31 33 37 44 45^ though several studies showed no change^34 36 39 40 42 47 51–53^ or even reduction^41^ in specific settings. One study distinguished between phases of the pandemic^36^ and demonstrated that mortality rates increased during infection surges and decreased during periods following lockdowns. Reported predictors of mortality encompassed advanced age, comorbidities and disease severity.^32 41 49^

##### Individual clinical outcomes

In 10 articles, 19 KPIs related to individual clinical outcomes were found across 6 specialties. These included obstetrics (Apgar scores, gestational age),^29^ stroke care (stroke severity),^35 47 53 54^ oncology (cancer stage, EUSOMA (European Society of Breast Cancer Specialist) indicators)^55 56^ and diabetes care.^57^ These indicators assess the performance of the hospital for certain diseases or patient groups, rather than for the entire patient population. Most indicators measured either the clinical severity at the time of presentation or condition-specific outcomes related to recovery, even though the definitions varied by specialty. Almost all of the studies looked at the time before and during COVID-19, and most of them reported minimal or no significant differences in these outcomes.^29 44 47 53–57^ Because these KPIs were specific to certain diseases, they were mostly used for assessments at the department or specialty level rather than for hospital-wide performance evaluations.

##### Readmissions and reoperations

10 KPIs were found in 6 specialties across 9 articles. These KPIs include unplanned hospital visits after discharge, such as 48-hour reoperations,^58^ early readmissions after outpatient surgery,^59^ 28-day,^40^ 30-day readmission rates,^60 61^ ICU readmissions^43^ and return visits to the ED within 72 hours.^51^ These indicators were primarily present in surgical and hospital-wide assessments. Some studies found no significant differences between the prepandemic and pandemic periods,^40 44 58 61^ while others noted minor increases or decreases^39 51 59^ based on the length of stay or the time frame examined. One study indicated variations during different phases of the pandemic.^51^

##### Clinical complications

In 14 articles, 24 KPIs were found in 7 specialties, with surgery and hospital-wide evaluations being the most common. Clinical complication KPIs show unintended outcomes that happen while a patient is in the hospital, such as infections after surgery,^52 60^ sepsis,^60 61^ venous thromboembolism,^60^ pressure ulcers^45 60^ or COVID-19^35 53 62^ that was acquired in the hospital. These indicators show new care-related conditions that were not present when the patient was admitted, so they demonstrate the hospital’s ability to maintain clinical safety, especially during periods of strain.

Postoperative infections^52 60^ and hospital-acquired COVID-19^35 53 62^ were the two clusters that appeared most frequently. Most studies examining the period before and after the COVID-19 pandemic found no significant changes in complication rates.^29 44 52–54 63^ However, some studies found that rates of infection-related complications went up during the pandemic.^39 47 53 61^

### Resource utilisation and capacity

#### Number of inpatients

Across 11 articles, 13 KPIs were identified. Among the involved medical specialties and hospital departments, infectious diseases and the ICU occur the most (3), followed by the ED (2). The number of inpatients is the KPI category that captures all variants measuring the patients who are or need to be hospitalised in the hospital. In the literature, it is also referred to as bed occupancy (level)^41 64^ or might also be labelled as bed census. We emphasise that it reflects the absolute number of patients, expressed as an integer value, hospitalised or awaiting hospitalisation over a given period. Most interest is in the number of COVID-19 confirmed inpatients, separated by care unit: ward and ICU (eg, see ^21 22^). This number of COVID-19 confirmed inpatients is commonly used to monitor the pressure exerted by COVID-19 patients on hospital bed capacity^22^ or to model potential future inpatient demand.^20 21^

#### Number of (available) beds

Across 11 articles, 13 KPIs were identified. For two of the KPIs the medical domain is not specified, one relates to bed availability in the ED, three to bed capacity for the infectious patient group and the remaining eight to hospital-wide bed availability. The number of available beds is the KPI category to capture hospital capacity in terms of physical bed resources. By the number of available beds, we consider the number of beds that could be staffed, also called the number of operational beds. This is because a bed without the required nurse or physical resource (eg, a mechanical ventilator) is inadequate for the patient. Similar to what we observed about the previous KPI category, the literature on the number of available beds focuses on the pressure that COVID-19 placed on hospital bed capacity. Of interest is the number of beds converted from non-COVID-19 use to (isolated) COVID-19 beds in dedicated wards (eg^23^) as well as the number of equipped beds in the ICU (eg^24^). Modelling studies examine scenarios aimed at increasing bed availability for infectious patients, for example, through the cancellation of routine surgical procedures.^25^ One study explores simulation outputs to assess the number of beds that could remain available for maintaining elective care.^26^

#### Number of staff

Across 8 articles, 15 KPIs were identified. Three of the KPIs related to staff availability for infectious patient care, three to the medical specialty of cardiology and for the remaining the medical domain is either not specified or are called ‘hospital-wide’. The availability of staff captures hospital capacity in terms of human resources. Across the reviewed literature, nurses, doctors (specified into junior and senior) and physicians are named as healthcare professionals. Support staff is not discussed in the included literature.

#### Utilisation

Across 6 articles, 10 KPIs were identified. The indicators covered four medical domains, most frequently infectious diseases (5), followed by cardiovascular care (3).

Utilisation in a hospital setting refers to the percentage of a given resource that is actively used compared with the total amount available, serving as a key indicator of operational efficiency and capacity management. The reviewed literature reported a variety of utilisation contexts, though most occurring were operating room (OR) utilisation and bed utilisation. Some studies used simulated measures to estimate the percentage of time resources used under modelled scenarios. Results from the included articles highlighted notable trends: total bed (hospital-wide) occupancy did not change during the pandemic, though the type of patients did—elective care patients vs infectious (COVID-19) patients.

#### Emergency department crowding

Across 4 articles, 11 KPIs were identified. KPIs in this category capture the extent of congestion within emergency care services. These indicators can be distinguished by several aspects: the number of patients present in the ED during a defined time window, the peak number of patients, the timing and duration of this peak, the times and duration during which all emergency rooms are fully occupied (so that additional patients are effectively blocked) and the counting of COVID-19 patients, including both suspected and confirmed cases. Terning *et al*^65^ note about the latter two measures (more often that the ED is fully occupied during the COVID-19 pandemic) were particularly impacted during the pandemic, as patient blockages occurred more frequently due to increased caution, with patients being kept longer in isolation in the ED while awaiting test results before further steps could be taken.

#### Efficiency scores

Across 3 articles, 7 KPIs were identified. Across the reviewed studies, efficiency scores emerged as a small category of indicators, typically designed as domain-specific or tailor-made composite measures to assess how effectively particular hospital processes operated during the pandemic. These metrics often integrate multiple time components or workflow elements into a single performance value, offering a more nuanced picture than isolated KPIs. One example is the anaesthetics efficiency score, defined as the sum of induction time, preparation time and sign-out time expressed as a fraction of the total OR time. This composite indicator captures how efficiently anaesthetic teams manage their procedural stages relative to overall surgical workflow. Notably, the study reporting this measure found that anaesthetics efficiency increased during the COVID-19 period, suggesting that adaptations made during the pandemic supported delivery of anaesthetic care^66^

### Access times and sojourn times

#### Access time

Across 17 articles, 35 KPIs were identified, mostly in oncology (11) and hospital-wide evaluations (10). Access-time KPIs measure delays occurring before patients enter hospital care, that is, the time spent outside the hospital setting prior to the initiation of care. Two broad types can be distinguished: measures expressed in time units (months, days, minutes) and counts, percentages, or binary indicators assessing whether patients receive care within or beyond a predefined timeframe. Most comparative studies reported significant changes in access time during the pandemic. Most articles did not discuss the validity or interpretability of these KPIs. One study noted a significant reduction in average access time from referral to surgery during the pandemic, coinciding with a marked decrease in elective surgeries performed.^27^

#### Length of stay

We found 33 KPIs across 24 articles. They were reported in 17 specialties, most often infectious diseases (5), hospital-wide evaluations (4), neurology (4) and emergency care (4). Length of stay (LOS) indicators measure the duration of hospitalisation. Reported measures include the total length of stay in the hospital, the length of stay on a ward, in the ICU or in the ED. These indicators were most often expressed in days, occasionally in hours. In some studies,^28–30^ length of stay was defined as the number or proportion of patients staying shorter or longer than a specified period. Most studies^27 29 31–36^ reported a decrease in length of stay during the COVID-19 pandemic, which can be explained by earlier discharge in order to reduce the risk of hospital-acquired COVID-19 infection.^37^ LOS was associated with other KPIs and characteristics, such as ward of discharge, year of discharge, type of procedure, complications during surgery and case fatality rate.^38 39^

#### Sojourn time

We found 44 KPIs in 20 articles across 11 different medical specialties, most frequently in cardiology and cardiovascular surgery (13), followed by neurology (9) and the ED (9). Sojourn time indicators describe time intervals after entry into the hospital, capturing the time patients spend within the hospital care process. This includes both clinical activities (eg, procedure duration) and in-hospital waiting times (eg, waiting in the ED). As length of stay (total duration of hospitalisation) is treated as a separate category, it is not included here. Time-based KPIs are strong indicators for quality of acute care, for example, stroke care.^40^ Reported measures include time from ED to admission, time from admission to surgery, stroke-specific KPIs such as door-to-CT time and door-to-needle time, waiting times and procedure times. Most KPIs were expressed in time units. For hip surgeries,^28 41^ door-to-physician time^30^ and reneging patients,^42^ the indicators were expressed as proportion, percentage or number of patients that received treatment within or beyond a specific timeframe. Changes in sojourn time during COVID-19 were explained by (reduced) OR capacity and personal protective equipment dressing.^43^

### Care delivery volumes

#### Admissions

We found 24 KPIs in 23 articles across 15 medical specialties, with hospital-wide evaluations (4) and the ED (3) reported most frequently. KPIs in this category describe volumes of patients entering the hospital, for different departments of the hospitals and different time frames: daily or weekly, although for some KPIs the time frame is not specified. Although closely linked to hospital capacity, admissions were classified as care delivery volumes because they describe the inflow of patients, rather than the level of occupied capacity at a given time. Most KPIs are measured in absolute numbers of patients.

#### Discharges

We found 12 KPIs in 9 articles in 7 different specialties, most often hospital-wide (3) and the ED (2). The KPIs are related to the outflow of both inpatients and outpatients from the hospital, mostly providing information on activity volume of the hospital or departments such as ED. They can also provide information on the number of diagnoses^43^ or in-hospital mortality,^26 27^ using the number of admissions.

#### Outpatient care activities

13 KPIs were found in 9 articles, in 7 different specialties, most frequently cardiology (3) and oncology (3). The subcategory includes KPIs describing volumes of outpatient care. Reported measures include the number of consultations, referrals and appointments. In all comparative studies including outpatient care KPIs, reductions during the pandemic were observed.^31 51 67–70^

#### Surgeries

14 KPIs were identified in 11 articles, distributed across 9 specialties, with hospital-wide evaluations (3) and orthopaedics (4, including surgical/trauma) most frequently represented. Indicators in this subcategory refer to the number of scheduled or performed surgical procedures, including waiting list volumes for surgery. Studies that compared surgery volumes pre-COVID-19 and during COVID-19 all found reduced numbers,^58 59 66–68 71 72^ although the ambulatory surgery rate (the fraction of surgeries that was performed in an ambulatory setting)^58^ and number of patients operated in specialised centres^73^ increased.

#### Non-surgical procedures

20 KPIs were reported in 11 articles, in 11 different specialties, with cardiology (5) and endocrinology (5) occurring most frequently. Reported KPIs include volumes of non-surgical procedures performed in the hospital, such as number of exams and number of patients receiving a specific therapy. Most comparative studies showed reductions during COVID-19,^47 53 54 67 74 75^ which raises concerns about the possible impact of the pandemic on regular care.^67^

#### Diagnoses

We found 8 KPIs in 7 articles, distributed over 6 specialties, with cardiology (2) and the ED (2) reported most frequently. The indicators reflect volumes related to specific diagnoses or diagnostic activity, such as the number of hospitalised patients with a specific diagnosis or newly diagnosed cases.

#### Case mix/patient complexity

In total, 3 KPIs were identified across 2 articles, reported in cardiology and internal medicine (2), and gynaecology/obstetrics (1). These KPIs capture the complexity and diversity of patient groups in the hospital and are related to the need for (number of/type of) resources.

#### Cancer screening volumes

We identified 3 KPIs across 2 articles. This subcategory covers indicators that evaluate population-based cancer screening activity and detection. Cancer screening was affected by the pandemic: a sharp increase was observed in detection rate in colonoscopy during COVID-19^76^ and the invitation coverage for breast cancer screening was markedly lower in 2020 compared with 2018 and 2019.^77^

### Care delivery performance

#### Process quality indicators

Altogether, 33 KPIs were identified across 8 articles. They are spread in an identical number of medical specialties or departments (8), where oncology is most frequently appearing (16), followed by gastroenterology (5) and the combined field of neurology and geriatrics (5). This subcategory comprises indicators that assess whether specific clinical actions or checks are performed by healthcare professionals as part of a patient’s treatment or care pathway.

#### Quality scores

In this subcategory, 15 KPIs were identified across 13 articles. KPIs were associated with several specialties, including hospital-wide assessments (3), gynaecology/obstetrics (including reproductive care) (2) and orthopaedics (2). By definition, all indicators in this subcategory were calculated scores; the input data were derived from electronic data records in all but two cases, which used survey data. As the name indicates, this category includes compound quality scores, which differ from process quality indicators as they represent calculated numerical measures summarising several individual quality checks. For example, in Böing *et al*,^78^ the total quality of care for patients treated for *Staphylococcus aureus* bloodstream infections was assessed through a scoring system in which the presence of each of six yes/no quality indicators contributed one point to a total score ranging from 0 to 6.

#### Missed care

Across 9 articles, 10 KPIs were identified. Missed care indicators capture instances where planned healthcare activities in a patient’s care pathway were not completed, either due to patient-related causes such as no-shows or inability to attend (eg, COVID-19 infection), or hospital-related causes, for example, cancellations resulting from staff shortages. The indicators covered a wide range of medical specialties, including hospital-wide assessments (1), intensive care (1), oncology (1) and the ED (1).

#### Laboratory performance (processed tests, turnaround times)

Ten KPIs were identified across three articles. Performance indicators related to clinical laboratories reflect the efficiency and productivity of these hospital support units, where patients are not physically present. These departments analyse biological materials such as blood, urine and tissue to assist in diagnosis, treatment decisions and disease monitoring. Typical indicators include turnaround times and the number of tests performed within a given time period. The performances of pathology departments were also considered within this category. 10 KPIs were identified across three articles. Two studies applied observational quantitative designs, and one was a modelling study that developed a data dashboard for monitoring COVID-19-related laboratory performance. The modelling study covered six KPIs referring to general, non-specified clinical laboratory operations, while two KPIs were specific to haematology laboratory performance and one to pathology and oncology. KPIs in this category are typically observed periodically and if there are sudden changes, it might be an indication that something within the process goes wrong.

#### Patient satisfaction

Three KPIs were identified across three articles. Patient satisfaction in hospital care refers to the patient’s perceived quality of their healthcare experience, encompassing both clinical and non-clinical aspects of care. It reflects how well healthcare services meet a patient’s expectations, needs and preferences. Typical indicators include overall satisfaction with the hospital stay or treatment, satisfaction with communication and information provided by healthcare professionals and comfort and cleanliness of the hospital environment. Patient satisfaction was investigated in four specialties: neurosurgery, endocrinology, gastroenterology and paediatric surgery.

#### Safety and adverse events

These KPIs covered a diverse range of measures, including inpatient falls, overnight stay rates following surgery, perceived patient safety, infection control staffing levels, alcohol-based hand rub usage, the proportion of patients without adverse events and hospitalisation rates following ambulatory procedures. In total, seven safety and adverse event KPIs were identified across six studies, making this one of the less frequently reported indicator subcategories. Despite their limited use, these indicators provided valuable insight into aspects of patient safety and the institutional environment that clinical complication KPIs alone may not capture. By focusing on preventable events, safety KPIs highlighted areas where structural resources, staff adherence and safety culture directly influenced patient outcomes during the pandemic. Electronic data recording and surveys are used as the two measurement methods, and almost all safety KPIs are used to compare pre-COVID-19 and COVID-19 periods.

### Other KPIs

#### Alternative care delivery

Six KPIs were found in 3 medical specialties across 3 articles. These KPIs show how hospital care has changed from being done in person to being done remotely or in an outpatient setting during the pandemic. Some indicators looked at how telemedicine or telehealth was used,^68 79^ and these studies showed that its use was growing steadily and significantly. One study showed that there was more than a 200% increase in phone clinics for people with heart failure and those who were recovering from heart surgery.^68^

The patient portal KPIs (waiting time, percentage of responses within target time and maximum acceptable waiting time)^79^ showed how important digital communication is becoming. The results showed a target-response rate of 71.6% and that portal users were more willing to wait than non-users. Surgical indicators like the substitution rate and the ambulatory rate showed that outpatient procedures were being done at a steady or slightly higher rate, with no major changes from before the pandemic.^59^

In general, these KPIs show that telehealth and other forms of care are being adopted quickly. Studies show that quality and efficiency stay the same even though people are moving away from in-person care.^58 67 78^

#### Other

Financial costs and home care were also defined as other KPIs in five articles. Three KPIs were described in terms of costs: operational costs,^60^ infection prevention cost^62^ and total healthcare expenditure.^49^ Two KPIs pointed out the number of home visits for care^68^ and the number of patients who received care at home.^80^

### Distribution of KPIs by reason for use across KPI categories and subcategories

Figures 5 and 6 demonstrate the number of KPIs for each category and subcategory, categorised by reason of use. A comparative analysis of KPIs revealed that the most prevalent categories were access and sojourn times (27.7%, n=74) and care delivery volumes (23.6%, n=63) when assessing the time period before and during the pandemic. The distribution of KPI categories varied based on the reason for use, thereby demonstrating that temporal comparisons were not uniformly implemented across KPI categories.

Care delivery performance indicators were the most common KPIs used to look into the quality of care during the COVID-19 pandemic, accounting for up to 30.7% (n=27) of all KPIs in this category. The distribution of KPI categories for this purpose also varied, suggesting a clear preference for process-oriented measures when assessing care quality under pandemic conditions.

In regard to the utilisation of KPIs to optimise decisions, the predominant categories were identified as resource utilisation and capacity (40.0%, n=32) and access and sojourn times (23.8%, n=19). Furthermore, the difference in the frequency with which KPI categories shows how closely operational decision-making is linked to capacity-related and time-related indicators. An analysis of the reasons for the application of KPIs reveals that the most common subcategories include sojourn time, mortality and process quality indicators, which are used for a wide range of purposes (figure 6). This shows that they can be used in a wide range of evaluative, comparative and decision-support settings.

**Figure 5:**
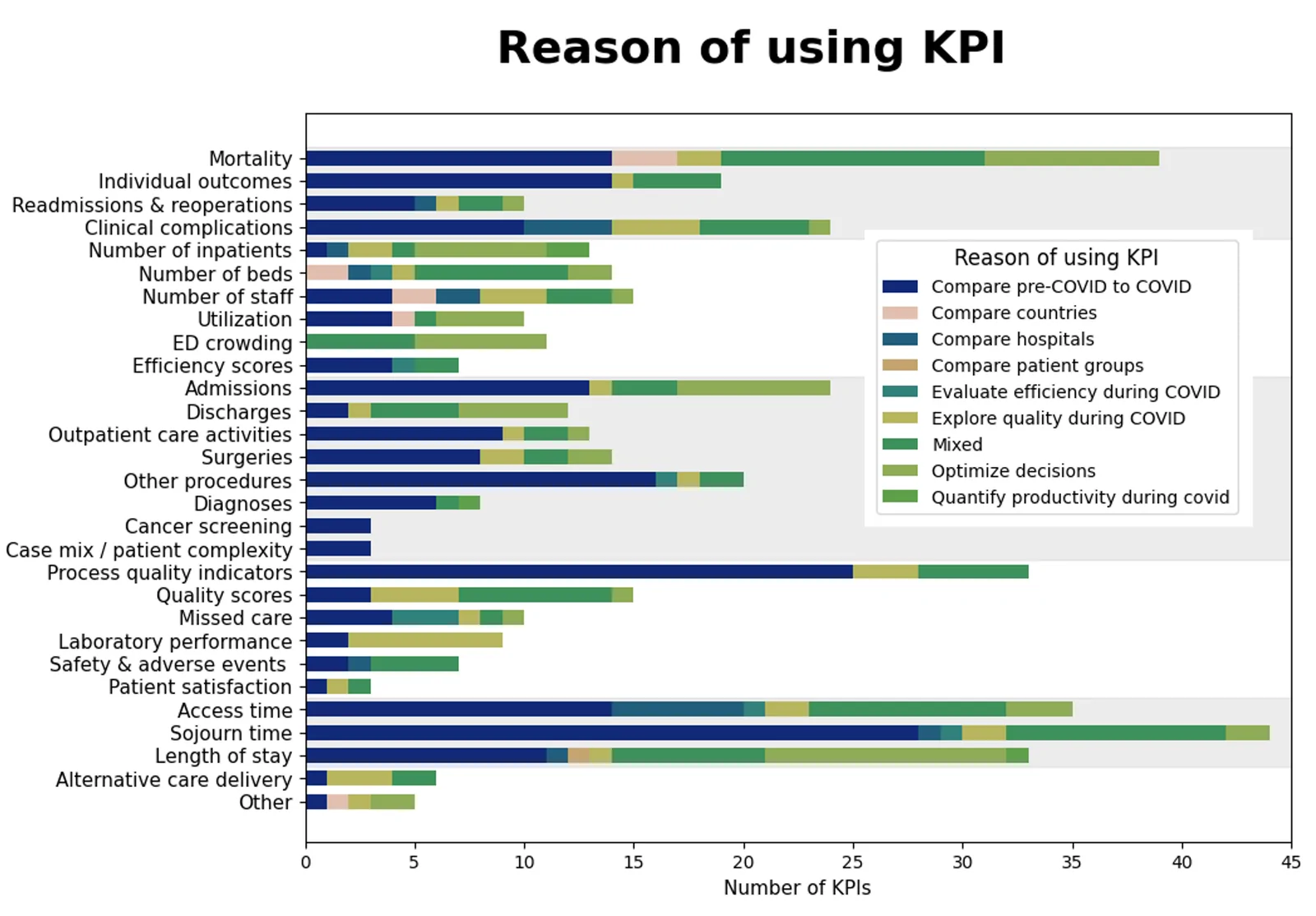
Distribution of numbers in KPI subcategories, categorised by reason of using KPI. ED, emergency department; KPI, key performance indicator.

**Figure 6:**
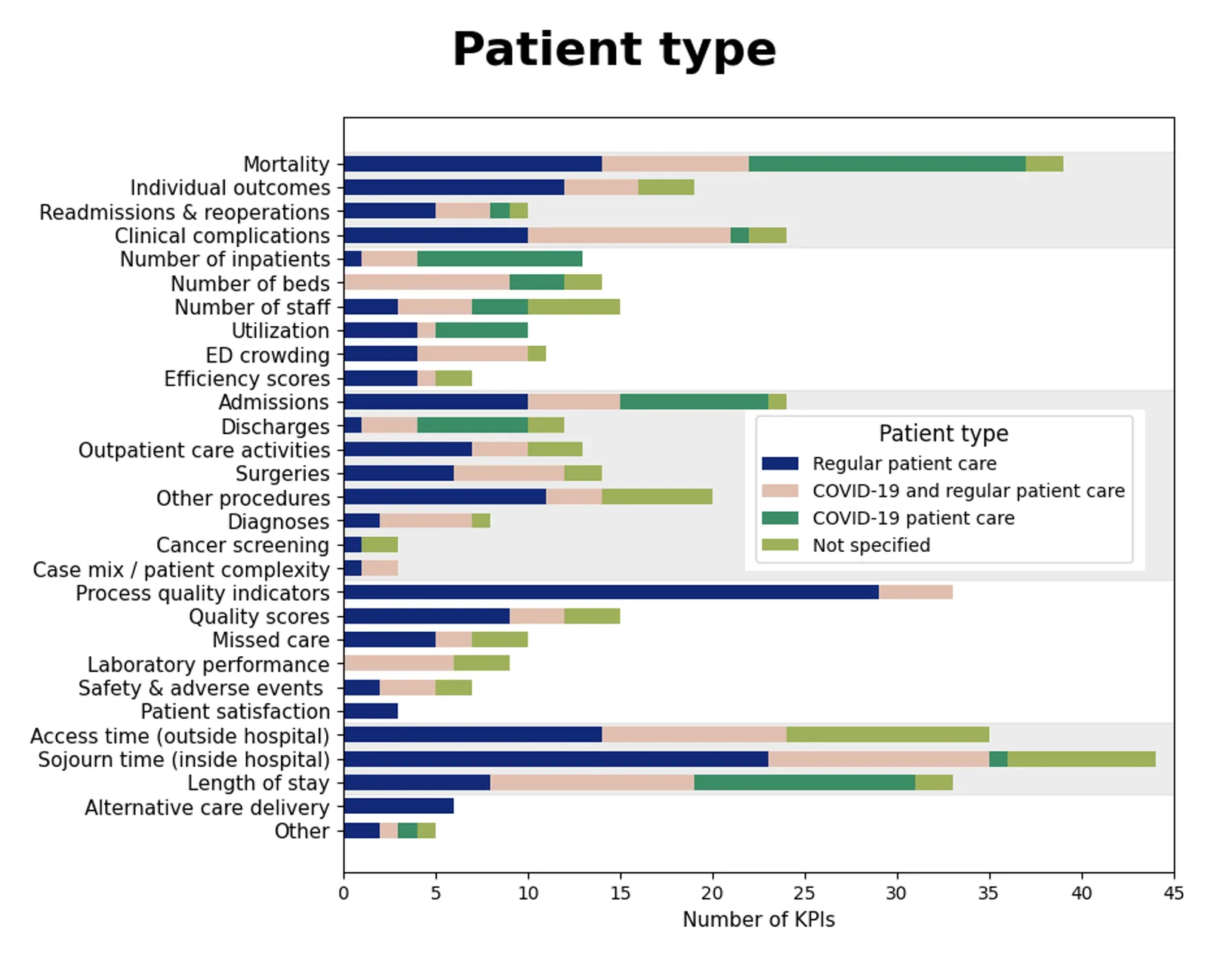
Distribution of KPIs by KPI subcategory across patient care groups. ED, emergency department; KPIs, key performance indicators.

### Distribution of KPIs by KPI categories and subcategory across patient care categories

Within KPIs assessing non-pandemic patient care, the *care delivery performance indicators* category was the most frequently represented, accounting for 25.3% (n=49) of all KPI categories. Among KPIs evaluating COVID-19 patient care, the most commonly used category was *resource utilisation and capacity* (31.7%, n=20). Studies assessing both COVID-19 and non-pandemic patient care showed the highest overall frequency compared with other categories, with *access and sojourn times* being the most frequently used KPI category within this category (26.0%, n=33).

When analysing each KPI category individually, *clinical outcomes* (28.9%, n=26), *access and sojourn times* (39.1%, n=43), *care delivery volumes* (40.9%, n=38) and *process quality indicators* (63.9%, n=49) were most frequently applied to evaluate non-pandemic patient care. In contrast, the *resource utilisation and capacity* category was most often used to assess both COVID-19 and non-pandemic patient care simultaneously (35.7%, n=25).

## Discussion

This study provides an overview of the KPIs used to evaluate hospital care services during COVID-19, integrating clinical and logistical perspectives. We also identified the reasons for using these KPIs, the method of measurement, the medical specialty, the study design in which KPIs were used and how KPIs were defined. We analysed 459 KPIs, found in 94 articles. Our findings show that there is a large variety of KPIs reported in the literature, expressed in diverse formats: volumes, scores, scales, percentages, characteristics and binary (yes/no) outcomes. The primary reasons for using KPIs were to compare pre-COVID-19 to COVID-19 periods (ie, specifically comparing two or more data sets), followed by supporting optimisation of decision making and exploring quality of care during COVID-19 (without explicit comparison with data from before the pandemic).

One of the main conclusions of our review is that clear and consistent KPI definitions are often lacking. Many articles that reported KPI measurements did not provide explicit definitions or sufficient detail regarding how the indicators were calculated. In several cases, even the expected outcome format (eg, percentage vs binary yes/no) remained unspecified. This may be due to several factors, such as the assumption that the KPI is self-explanatory or widely understood within the field. However, this lack of clarity makes it difficult to interpret results, compare findings across studies that use the ‘same’ KPI, reproduce research or reuse the indicator in future work. On the other hand, some KPIs were given different definitions by different studies. The reverse also occurred, with KPIs measuring the same quantity but appearing under different names across studies, for example, access time and waiting time. These findings illustrate that, without clear definitions and uniform terminology, it becomes difficult to compare performance between hospitals or specialties. This shows the need for transparent KPI descriptions when the goal is to assess and compare hospital quality.

Assessing performance during pandemics likely requires consideration of both patient outcomes and organisational processes to obtain a comprehensive view. However, our findings show that studies rarely combine clinical and logistical perspectives within the same performance assessment. While our review identified KPIs across both domains, most individual studies tended to emphasise either clinical outcomes (eg, mortality, complications) or operational KPIs (eg, activity volumes, access times), rather than considering these jointly. This may be explained by the different perspectives and data requirements underlying clinical and logistical performance, which make integration challenging. In addition, we found that most KPIs used during COVID-19 were not specific to the pandemic but were indicators already used in routine performance monitoring. Only a limited number of measures, such as numbers of hospital-acquired COVID-19 infections, COVID-19 mortality and length of stay of COVID-19 patients seem to be unique to the pandemic context. This suggests that many of the KPIs needed to evaluate care during future health emergencies are already available and can be anticipated in advance. Future research could explicitly compare KPIs used during pandemics with those applied in non-crisis settings, to better understand how performance indicators used during pandemics differ from those applied in routine hospital care.

This review has several strengths. One of the main strengths of this review is its broad, hospital-wide perspective. We did not focus on a single specialty, but mapped KPIs across many (clinical) areas. In addition, we included studies from medical, organisational and operations research perspectives. This allowed us to capture different ways in which hospital performance was evaluated during the pandemic. The multidisciplinary composition of the research team, combining clinical and operations research expertise, supported the interpretation and contextualisation of findings across these domains. However, several limitations should be noted. We restricted our search to European countries and English-language publications, which may limit the representativeness of our findings and excludes relevant work from other regions. In addition, this review focused exclusively on the COVID-19 pandemic. As a result, the identified KPIs largely reflect the characteristics and challenges specific to COVID-19 and may not fully capture performance aspects that would become relevant in future pandemics with different epidemiological features. We also excluded grey literature, which may have resulted in missing indicators used in practice, but not reported in peer-reviewed publications. Although we aimed to include a broad range of terms related to KPIs and performance evaluation in our search strategy, the list used in our search string may not have been exhaustive and relevant studies identifying or evaluating KPIs may therefore have been missed. Furthermore, as this was a scoping review, we did not perform a formal critical appraisal of the included studies, and therefore the quality and validity of the reported KPI measurements were not assessed. Finally, the inductive categorisation of KPIs involves interpretation by the reviewers and may therefore reflect some degree of subjectivity. Different approaches to grouping KPIs may lead to alternative categorisations. For example, some KPIs could reasonably fit into multiple categories depending on the perspective taken.

Overall, this scoping review brings together a broad overview of how hospital performance was measured during the COVID-19 pandemic and shows a substantial variation in how KPIs are defined and reported. Our findings highlight the need for clearer and more consistent KPI descriptions in both research and practice. In particular, specifying the KPI definition, the outcome format, the timeframe and the formula used, may improve the interpretation, comparability and reproducibility of reported results. For hospitals and policymakers, our overview may support discussions on which indicators could be considered when monitoring performance during crises. In particular, combining clinical and logistical KPIs could provide a broader perspective on hospital performance during crises, where clinical outcomes, capacity constraints and organisational considerations need to be considered together. Future work could examine how different KPIs relate to each other, as this may help identify groups of indicators that capture important aspects of hospital performance. It may also be valuable to explore more uniform definitions for commonly used KPIs, which would strengthen the comparability across studies and improve preparedness for future pandemics.

## Supplementary material

10.1136/bmjopen-2026-122362 online supplemental file 1

10.1136/bmjopen-2026-122362 online supplemental file 2

10.1136/bmjopen-2026-122362 online supplemental file 3

## Data Availability

Data are available upon reasonable request.
