# Supplementary material for: Performance indicators for evaluating and optimising hospital services during COVID-19: a scoping review
Source: BMJ Open. 2026 Sep 28;16(9):e122362. doi: 10.1136/bmjopen-2026-122362 (PMC13630113; doi:10.1136/bmjopen-2026-122362)
Supplement: Supplementary file 1 [file bmjopen-16-9-s002.pdf]

Appendix B: Key performance indicators for evaluating and optimising hospital services during COVID-19: overview of included studies

Table 1: Overview of included studies

Study title	Year	Country	Study design	Hospital type(s)	Specialty	Patient type
Quality of maternal and newborn care in Switzerland during the COVID-19 pandemic: A cross-sectional study based on WHO quality standards [1]	2022	Switzerland	Observational - Quantitative	Multiple hospitals - Mixed	Gynaecology/Obstet (incl. reproductive care)	Regular
COVID-19 Pandemic and Stroke Care in Patients with Dementia Compared to Other Stroke Patients [2]	2024	Sweden	Observational - Quantitative	Multiple hospitals - Mixed	Neurology,Geriatry	Regular
Dealing with uncertainty in healthcare performance assessment: a fuzzy network-DEA approach with undesirable outputs [3]	2024	Portugal	Modelling study,Observatic - Quantitative	Multiple hospitals - Mixed	Hospital-wide	Mixed
Quality of acute myocardial infarction care in England and Wales during the COVID- 19 pandemic: linked nationwide cohort study [4]	2021	England,Wales	Observational - Quantitative	Multiple hospitals - Mixed	Cardiology	Regular
Maintenance of Acute Stroke Care Service During the COVID-19 Pandemic Lockdown [5]	2021	Multiple European (>5)	Observational - Quantitative	Multiple hospitals - Mixed	Neurology	Regular

Continued on next page

Table 1: Overview of included studies

Study Title	Year	Country(ies)	Study design	Hospital type(s)	Medical specialty	Patient type
Operating room efficiency and timing during coronavirus disease 2019 outbreak in a referral orthopaedic hospital in Northern Italy [6]	2020	Italy	Observational - Quantitative	Single hospital - Specialty	Orthopedics (surgical),Trauma	Mixed
Assessing the Efficiency and Productivity of the Hospital Clinics on the Island of Rhodes during the COVID-19 Pandemic [7]	2022	Greece	Observational - Quantitative,Modelling study	Single hospital - General	Hospital-wide	Mixed
COVID-19 pandemic and hip fractures: impact and lessons learned [8]	2020	United Kingdom	Observational - Quantitative	Single hospital -Academic	Orthopedics (surgical),Trauma	Mixed
Did Ambulatory Surgical Units succeed in adapting to COVID-19 pandemic? The experience of a Portuguese Hospital with Ambulatory Surgery [9]	2021	Portugal	Observational - Quantitative	Single hospital - General	Hospital-wide	Regular
Impact of COVID- 19 pandemic on the quality of test output in haemostasis laboratories [10]	2022	The Netherlands	Observational - Quantitative	Multiple hospitals - Mixed	Haematology	Not specified
A Simulation-Based Cost-Effectiveness Analysis of Severe Acute Respiratory Syndrome Coronavirus 2 Infection Prevention Strategies for Visitors of Healthcare Institutions [11]	2022	Germany	Modelling study	Single hospital - General	Hospital-wide	Not specified
COVID-19 Pandemic: Huge Stress Test for Health System Could Be a Great Opportunity to Update the Workflow in a Modern Surgical Pathology [12]	2021	Italy	Observational - Quantitative	Single hospital - Specialty	Pathology,Oncology	Regular
Quality in stroke care during the early phases of the COVID-19 pandemic: A nationwide study [13]	2022	Denmark	Observational - Quantitative	Multiple hospitals - Mixed	Neurology	Regular

Continued on next page

Table 1: Overview of included studies

Study Title	Year	Country(ies)	Study design	Hospital type(s)	Medical specialty	Patient type
Impact of the COVID-19 Pandemic on the Management of Staphylococcus aureus Bloodstream Infections in a Tertiary Care Hospital [15]	2023	Germany	Observational - Quantitative	Single hospital -Academic	Infectious diseases	Regular
Effect of the COVID-19 pandemic and the lockdown measures on the local stroke network[14]	2021	Italy	Observational - Quantitative	Multiple hospitals - Mixed	Neurology	Regular
The Portuguese Public Hospitals Performance Evolution before and during the SARS-CoV-2 Pandemic (2017-2022) [16]	2023	Portugal	Modelling study	Multiple hospitals - Mixed	Hospital-wide	Not specified
Improving prediction of COVID-19 mortality using machine learning in the Spanish SEMI-COVID-19 registry [17]	2023	Spain	Modelling study	Single hospital - General	Infectious diseases	COVID-19
CNN Approach for Predicting Survival Outcome of Patients With COVID-19 [18]	2023	France	Modelling study	Single hospital - General	Infectious diseases	COVID-19
Readjustment of Capsule Endoscopy Protocols to the COVID-19 Pandemic in a Portuguese Tertiary Center [19]	2022	Portugal	Observational - Quantitative	Single hospital -Academic	Gastroenterology	Regular
Hip fracture care during Covid-19: a regional trauma centre's experience [20]	2021	Ireland	Observational - Quantitative	Single hospital - Specialty	Orthopedics (surgical)	Regular
Impact of COVID-19 Pandemic on Colorectal Cancer Screening Program [24]	2020	Italy	Observational - Quantitative	Single hospital - Unknown	Gastroenterology	Regular
Rhythmicity of patient flow in an acute medical unit: relationship to hospital occupancy, seven-day working, and the effect of COVID-19 [21]	2022	England	Observational - Quantitative	Single hospital - Unknown	Emergency department (ED),Hospital-wide	Mixed

Continued on next page

Table 1: Overview of included studies

Study Title	Year	Country(ies)	Study design	Hospital type(s)	Medical specialty	Patient type
The COVID-19 Impact on Oral Healthcare Demand and Performance: The Experience of a Clinical and Academic Centre in Portugal (EU) [22]	2023	Portugal	Observational - Quantitative	Single hospital -Academic	Dentistry	Regular
Impact of COVID-19 on Hospital Admissions and Healthcare Quality Indicators in Non-COVID Patients: A Retrospective Study of the First COVID-19 Year in a University Hospital in Spain [23]	2022	Spain	Observational - Quantitative	Single hospital -Academic	Hospital-wide	Regular
Evaluating the impact of an enhanced triage process on the performance and diagnostic yield of oesophageal physiology studies post COVID- 19 [25]	2021	England	Observational - Quantitative	Single hospital -Academic	Gastroenterology	Regular
A Comparison of Time-Series Predictions for Healthcare Emergency Department Indicators and the Impact of COVID-19 [26]	2021	England	Modelling study	Single hospital - Unknown	Emergency department (ED)	Regular
Impact of the COVID-19 pandemic on the quality of diabetes care: Insights from longitudinal data in Switzerland [27]	2023	Switzerland	Observational - Quantitative		Endocrinology	Not specified
Real-time prediction of COVID-19 patients health situations using Artificial Neural Networks and Fuzzy Interval Mathematical modeling [28]	2021	Portugal	Modelling study	Single hospital - Unknown	Infectious diseases	COVID-19

Continued on next page

Table 1: Overview of included studies

Study Title	Year	Country(ies)	Study design	Hospital type(s)	Medical specialty	Patient type
Impact of COVID-19 pandemic on key performance indicators in pancreatobiliary endoscopy: prioritise, minimise risk, keep scoping and training [29]	2021	United Kingdom	Observational - Quantitative	Multiple hospitals - Mixed	Gastroenterology	Regular
Missed nursing care in the critical care unit, before and during the COVID-19 pandemic: A comparative cross-sectional study [30]	2022	Sweden	Observational - Quantitative	Single hospital -Academic	ICU	Mixed
Physician resilience and perceived quality of care among medical doctors with training in psychosomatic medicine during the COVID-19 pandemic: a quantitative and qualitative analysis [31]	2024	Austria	Observational - Quantitative	Multiple hospitals - Mixed	Hospital-wide	Mixed
The impact of the COVID-19 pandemic on cardiology services [32]	2020	Scotland	Observational - Quantitative	Single hospital - General	Cardiology	Regular
Does the length of stay in hospital affect healthcare outcomes of patients without COVID-19 who were admitted during the pandemic? A retrospective monocentric study [33]	2022	England	Observational - Quantitative	Single hospital - General	Hospital-wide	Regular
Effect of lockdown on the management of ischemic stroke: an Italian experience from a COVID hospital [34]	2020	Italy	Observational - Quantitative	Single hospital -Academic	Neurology	Mixed
The impact of the COVID-19 pandemic on the performance of the Rapid Access Lung Cancer Clinic [35]	2024	Ireland	Observational - Quantitative	Single hospital -Academic	Oncology	Regular

Continued on next page

Table 1: Overview of included studies

Study Title	Year	Country(ies)	Study design	Hospital type(s)	Medical specialty	Patient type
Maximizing hospital capacity to serve pandemic patient surge in hot spots via queueing theory and microsimulation [36]	2022		Modelling study	Single hospital - Unknown	Infectious diseases,ICU	COVID-19
Economic impact of avoidable cancer deaths caused by diagnostic delay during the COVID-19 pandemic: A national population-based modelling study in England, UK [37]	2021	England	Modelling study	Multiple hospitals - Mixed	Oncology	Regular
The management of emergency spinal surgery during the COVID-19 pandemic in Italy [38]	2020	Italy	Observational - Quantitative	Single hospital - General	General surgery	Regular
Hybridizing adaptive large neighborhood search with kernel search: a new solution approach for the nurse routing problem with incompatible services and minimum demand [39]	2022	Italy	Modelling study	Single hospital - Unknown	Hospital-wide	Regular
A systematized approach for reduction of medical appointment waiting list [41]	2022	Portugal	Modelling study,Observatic - Quantitative	Single hospital - General	Orthopedics	Regular
Model-informed health system reorganization during emergencies [42]	2023	Switzerland	Modelling study	Multiple hospitals - type unknown	Infectious diseases	COVID-19
Machine-learning-derived predictive score for early estimation of COVID-19 mortality risk in hospitalized patients [40]	2022	Spain	Modelling study,Observatic - Quantitative	Multiple hospitals - Mixed	Infectious diseases	COVID-19
Quality in Clinical Consultations: A Cross-Sectional Study [43]	2022	England	Observational - Quantitative	Single hospital -Academic	Neurosurgery,Endocr	Regular

Continued on next page

Table 1: Overview of included studies

Study Title	Year	Country(ies)	Study design	Hospital type(s)	Medical specialty	Patient type
Chronological Development of Cardiovascular Disease in Times of COVID-19: A Retrospective Analysis of Hospitalized Diseases of the Circulatory System and COVID-19 Patients of a German University Hospital [44]	2022	Germany	Observational - Quantitative	Single hospital -Academic	Cardiology,Internal medicine	Mixed
Impact of the COVID-19 Pandemic on Delivery of Gynecology and Obstetrics Services at a Maximum Care University Hospital in Germany [45]	2022	Germany	Observational - Quantitative	Single hospital -Academic	Gynaecology/Obstet (incl. reproductive care)	Regular
Indirect effect of the COVID-19 pandemic on hospital mortality in patients with hip fracture: A competing risk survival analysis using linked administrative data [46]	2023	United Kingdom	Observational - Quantitative	Multiple hospitals - Mixed	Orthopedics	Regular
Preliminary Analysis of the Impact of the Coronavirus Disease 2019 Outbreak on Italian Liver Transplant Programs [47]	2020	Italy	Observational - Quantitative	Multiple hospitals - all Specialty	Transplantation	COVID-19
Palliative care need screening and specialised referrals fell during the COVID-19 pandemic: a nationwide register-based study [48]	2023	Denmark	Observational - Quantitative	Multiple hospitals - all Specialty	Palliative care/end-of-life	Mixed
Assessing the impact of COVID-19 on the efficiency of Portuguese state-owned enterprise hospitals [49]	2022	Portugal	Modelling study	Multiple hospitals - type unknown	Not specified	Not specified
Neurotrauma admissions and COVID-19: a National Centre experience [50]	2021	Ireland	Observational - Quantitative	Multiple hospitals - all Specialty	Trauma	Regular

Continued on next page

Table 1: Overview of included studies

Study Title	Year	Country(ies)	Study design	Hospital type(s)	Medical specialty	Patient type
Comparing length of hospital stay during COVID-19 pandemic in the USA, Italy and Germany [51]	2022	Italy,Germany	Modelling study	Multiple hospitals - type unknown	Not specified	COVID-19
Flemish population-based cancer screening programs: impact of COVID-19 related shutdown on short-term key performance indicators [52]	2022	Belgium	Observational - Quantitative	Multiple hospitals - type unknown	Cancer screening	Not specified
Safety and Operational Efficiency of Restructuring and Redeploying a Transcatheter Aortic Valve Replacement Service During the COVID-19 Pandemic: The Oxford Experience [53]	2020	United Kingdom	Observational - Quantitative	Single hospital -Academic	Cardiovascular surgery	Mixed
Factors affecting portal usage among chronically ill patients during the COVID-19 pandemic in the Netherlands: Cross-sectional study [54]	2021	The Netherlands	Observational - Quantitative	Multiple hospitals - Mixed	Not specified	Regular
Performance analysis of English hospitals during the first and second waves of the coronavirus pandemic [55]	2023	England	Observational - Quantitative,Modelling study	Multiple hospitals - Mixed	Infectious diseases	COVID-19
Covid-19 mortality is negatively associated with test number and government effectiveness [56]	2020	Multiple European (>5)	Observational - Quantitative	Multiple hospitals - Mixed	Infectious diseases	COVID-19
Antimicrobial stewardship programs in acute-care hospitals: A multicenter assessment of structure, process, and outcome indicators in Italy and Spain [57]	2024	Italy,Spain	Observational - Quantitative	Multiple hospitals - type unknown	Infectious diseases	Not specified

Continued on next page

Table 1: Overview of included studies

Study Title	Year	Country(ies)	Study design	Hospital type(s)	Medical specialty	Patient type
Estimating lengths-of-stay of hospitalised COVID-19 patients using a non-parametric model: a case study in Galicia (Spain) [60]	2021	Spain	Modelling study	Multiple hospitals - type unknown	Infectious diseases	COVID-19
Highly specialized Breast Centers did not experience delay of care during COVID-19 pandemic in Italy: the Senonetwork experience [58]	2022	Italy	Observational - Quantitative	Multiple hospitals - Mixed	Oncology	Regular
Impact of COVID-19 on operation room utilization efficiency and cardiac surgery care pathway: single centre experience [59]	2023	United Kingdom	Observational - Quantitative	Single hospital -Academic	Cardiovascular surgery	Regular
Worldwide and Country-Specific Impact of the COVID-19 Pandemic on Heart Transplantation Volumes: A Longitudinal Analysis of 2020 and 2021 [61]	2023	Multiple European (>5)	Observational - Quantitative	Multiple hospitals - Mixed	Cardiology,Transplan	Regular
Healthcare indicators associated with COVID-19 death rates in the European Union [62]	2021	Multiple European (>5)	Observational - Quantitative	Multiple hospitals - type unknown	Infectious diseases	COVID-19
An Automated Dashboard to Improve Laboratory COVID-19 Diagnostics Management [63]	2021	Switzerland	Modelling study	Single hospital -Academic	Laboratory	Mixed
Quantifying the impact of hospital catchment area definitions on hospital admissions forecasts: COVID-19 in England, September 2020–April 2021 [64]	2024	England	Modelling study	Multiple hospitals - type unknown	Not specified	COVID-19
Balancing scarce hospital resources during the COVID-19 pandemic using discrete-event simulation [65]	2021	United Kingdom,The Netherlands	Modelling study,Observatic - Quantitative	Single hospital -Academic	Hospital-wide	Mixed

Continued on next page

Table 1: Overview of included studies

Study Title	Year	Country(ies)	Study design	Hospital type(s)	Medical specialty	Patient type
Prevalence and impact of SARS-CoV-2 infection among patients with acute ischaemic stroke: a nationwide register-based cohort study in Denmark [66]	2024	Denmark	Observational - Quantitative	Multiple hospitals - Mixed	Stroke specific	Regular
Performance status: A key factor in predicting mortality in the first wave of COVID-19 in South-East Scotland [67]	2022	Scotland	Modelling study	Multiple hospitals - type unknown	Not specified	COVID-19
Strategies to convert hospital beds for COVID-19 patients to minimize emergency department overcrowding [68]	2023	Italy	Observational - Quantitative,Modelling study	Multiple hospitals - Mixed	Infectious diseases	Mixed
Evaluating Portuguese Public Hospitals Performance: Any Difference before and during COVID-19? [69]	2023	Portugal	Modelling study	Multiple hospitals - Mixed	Hospital-wide	Mixed
Detecting Regional Differences in Italian Health Services during Five COVID-19 Waves [70]	2023	Italy	Observational - Quantitative	Multiple hospitals - type unknown	ICU	COVID-19
Impact of COVID-19 pandemic on hip fractures: the central London experience COVID-related urgent geriatric hip trauma (COUGH) study COVERT (COVid Emergency-Related Trauma and orthopaedics) collaborative [71]	2022	England	Observational - Quantitative	Single hospital - Unknown	Orthopedics,Orthope (surgical),Trauma	Regular
Incidence, Quality of Care, and Mortality in Hip Fracture Patients in the Course of the COVID Pandemic: A Population-Based Cohort Study [72]	2024	Denmark	Observational - Quantitative	Multiple hospitals - Mixed	Orthopedics	Mixed

Continued on next page

Table 1: Overview of included studies

Study Title	Year	Country(ies)	Study design	Hospital type(s)	Medical specialty	Patient type
Impact of the coronavirus disease 2019 pandemic on sarcoma management in France: a 2019 and 2020 comparison [73]	2023	France	Observational - Quantitative	Multiple hospitals - Mixed	Orthopedics (surgical)	Not specified
A Finite-State Machine Approach to Study Patients Dropout From Medical Examinations [74]	2021	Italy	Modelling study	Not specified	Emergency department (ED)	Not specified
Impact of COVID-19-Related Lockdown on Delivery and Perinatal Outcomes: A Retrospective Cohort Study [75]	2022	France	Observational - Quantitative	Multiple hospitals - all Academic	Obstetrics	Regular
Effect of cancer waiting time standards in the English National Health Service: a threshold analysis [76]	2024	England	Observational - Quantitative	Multiple hospitals - type unknown	Oncology	Not specified
Assessment of quality indicators in pediatric major outpatient surgery. Influence of the COVID-19 pandemic [77]	2023	Spain	Observational - Quantitative	Single hospital - Specialty	Pediatric Surgery	Regular
SARS-CoV-2 Testing and Patient Waiting Times in the Emergency Department [78]	2022	Ireland	Observational - Quantitative	Single hospital -Academic	Emergency department (ED)	Not specified
Flexibility and Bed Margins of the Community of Madrid's Hospitals during the First Wave of the SARS-CoV-2 Pandemic [81]	2021	Spain	Modelling study	Multiple hospitals - Mixed	Hospital-wide	Mixed
Impact of COVID-19 outbreak on regional STEMI care in Germany [79]	2020	Germany	Observational - Quantitative	Multiple hospitals - type unknown	Cardiology	Regular

Continued on next page

Table 1: Overview of included studies

Study Title	Year	Country(ies)	Study design	Hospital type(s)	Medical specialty	Patient type
Delayed Comprehensive Stroke Unit Care Attributable to the Evolution of Infection Protection Measures across Two Consecutive Waves of the COVID-19 Pandemic [80]	2021	Germany	Observational - Quantitative	Single hospital - Specialty	Emergency department (ED)	Mixed
Modeling Patient Flow in an Emergency Department under COVID-19 Pandemic Conditions: A Hybrid Modeling Approach [82]	2022	Norway	Modelling study	Single hospital - Unknown	Emergency department (ED)	Mixed
Convolutional Neural Network-Based Automatic Analysis of Chest Radiographs for the Detection of COVID-19 Pneumonia: A Prioritizing Tool in the Emergency Department, Phase I Study and Preliminary "Real Life" Results [83]	2022	Italy	Modelling study	Single hospital -Academic	Emergency department (ED)	Mixed
Flow Analysis of Mastectomy Patients Using Length of Stay: A Single-Center Study [84]	2024	Italy	Observational - Quantitative	Single hospital -Academic	General surgery	Mixed
The lung ultrasound "Rule of 7" in the prognosis of COVID-19 patients: Results from a prospective multicentric study [85]	2021	Spain	Modelling study	Multiple hospitals - all Academic	Emergency department (ED)	COVID-19
Can we reliably automate clinical prognostic modelling? A retrospective cohort study for ICU triage prediction of in-hospital mortality of COVID-19 patients in the Netherlands [86]	2022	The Netherlands	Modelling study,Observatic - Quantitative	Multiple hospitals - Mixed	ICU	COVID-19
Emergency Department Visits among Cancer Patients during SARS-CoV-2 Pandemic [87]	2023	Italy	Observational - Quantitative	Single hospital -Academic	Emergency department (ED)	Mixed

Continued on next page

Table 1: Overview of included studies

Study Title	Year	Country(ies)	Study design	Hospital type(s)	Medical specialty	Patient type
The impact of the SARS-COV-2 pandemic on the quality of breast cancer care in EUSOMA-certified breast centres [88]	2022	Multiple European (>5)	Observational - Quantitative	Multiple hospitals - Mixed	Oncology	
The presentations/physician ratio predicts door-to-physician time but not global length of stay in the emergency department: an Italian multicenter study during the SARS-CoV-2 pandemic [89]	2022	Italy	Observational - Quantitative	Multiple hospitals - Mixed	Emergency department (ED)	Not specified
Impact of COVID-19 on Key Performance Indicators of the National Hip Fracture Database and the Management of Hip Fracture Patients [90]	2021	Wales	Observational - Quantitative	Single hospital - Specialty	Orthopedics (surgical)	
Hospitalization forecast to inform COVID-19 pandemic planning and resource allocation using mathematical models [91]	2024	Spain	Modelling study	Multiple hospitals - all Academic	Not specified	COVID-19
Modeling the Recovery of Elective Waiting Lists Following COVID-19: Scenario Projections for England [92]	2022	England	Modelling study	Not specified	Not specified	Not specified
COVID-19 scenario modelling for the mitigation of capacity-dependent deaths in intensive care [93]	2020	England	Modelling study	Single hospital - General	ICU	COVID-19
Improving ICSI success rates following root cause analysis and use of system behaviour charts: the devil is in the detail [94]	2022	United Kingdom	Observational - Quantitative	Single hospital - Specialty	Gynaecology/Obstet (incl. reproductive care)	Regular

References

[1] A. Abderhalden-Zellweger, S. Grylka-Baeschlin, A. N. Mueller, E. P. Valente, A. Cerimagic, D. Drandic, M. Kurbanovic, R. Virginie, E. de La Rochebrochard, K. Lofgren, C. Miani, S. Batram-Zantvoort, L. Wandschneider, M. Lazzerini, B. Covi, I. Mariani, S. Morano, I. Chertok, E. Hefer, R. Artzi-Medvedik, E. Pumpure, D. Rezeberga, G. Jansone-Santare, D. Jakovicka, A. R. Knoka, K. P. Vilcane, A. Liepinaitiene, A. Kondrakova, M. Mizgaitiene, S. Juciute, M. Arendt, B. Tasch, I. H. Nedberg, S. Kongslien, E. S. Vik, B. Baranowska, U. Tataj-Puzyna, M. Wegrzynowska, R. Costa, C. Barata, T. Santos, C. Rodrigues, H. Dias, M. R. Otelea, J. Radetic, J. Ruzicic, Z. Drglin, B. M. Ponikvar, A. Bohinec, S. Brigidi, L. M. Castaneda, H. Elden, V. Sengpiel, K. Linden, M. Zaigham, C. De Labrusse, A. Abderhalden, A. Pfund, H. Thorn, S. Grylka, M. Gemperle, and A. Mueller. Quality of maternal and newborn care in Switzerland during the COVID-19 pandemic: A cross-sectional study based on WHO quality standards. *International Journal of Gynecology and Obstetrics*, 159(S1):70–84, 2022. Place: Ireland.

[2] T. Abzhandadze, M. T. Hoang, M. Mo, S. Mostafaei, P. G. Jurado, H. Xu, K. Johnell, M. Von Euler, M. Eriksdotter, and S. Garcia-Ptacek. COVID-19 Pandemic and Stroke Care in Patients with Dementia Compared to Other Stroke Patients. *Journal of the American Medical Directors Association*, 25(7):105011, 2024. Place: United States.

[3] G. P. Afonso, J. R. Figueira, and D. C. Ferreira. Dealing with uncertainty in healthcare performance assessment: a fuzzy network-DEA approach with undesirable outputs. *INTERNATIONAL TRANSACTIONS IN OPERATIONAL RESEARCH*, 2024.

[4] Suleman Aktaa, Mohammad E. Yadegarfar, Wu Jianhua, Muhammad Rashid, Mark de Belder, John Deanfield, Francois Schiele, Mark Minchin, Mamas Mamas, and Chris P. Gale. Quality of acute myocardial infarction care in England and Wales during the COVID-19 pandemic: linked nationwide cohort study. *BMJ Quality & Safety*, 31(2):116–122, 2022.

[5] V. L. Altersberger, L. J. Stolze, M. R. Heldner, H. Henon, N. Martinez-Majander, C. Hametner, A. Nordanstig, A. Zini, S. Nannoni, B. Gonçalves, C. H. Nolte, P. Baumgartner, A. Kastrup, P. Papanagiotou, G. Kägi, R. R. Leker, M. Zedde, A. Padovani, A. Pezzini, V. Padjen, C. W. Cereda, G. Ntaios, L. H. Bonati, L. A. Rinkel, U. Fischer, J. F. Scheitz, S. Wegener, G. Turc, P. Michel, M. Gentile, A. Rentzos, P. A. Ringleb, S. Curtze, C. Cordonnier, M. Arnold, P. J. Nederkoorn, S. T. Engelter, and H. Gensicke. Maintenance of Acute Stroke Care Service during the COVID-19 Pandemic Lockdown. *Stroke*, 52(5), 2021.

[6] M. Andreata, M. Faraldi, E. Bucci, G. Lombardi, and L. Zagra. Operating room efficiency and timing during coronavirus disease 2019 outbreak in a referral orthopaedic hospital in Northern Italy. *International Orthopaedics*, 44(12):2499–2504, 2020.

[7] L. Androutsou, M. Kokkinos, D. Latsou, and M. Geitona. Assessing the Efficiency and Productivity of the Hospital Clinics on the Island of Rhodes during the COVID-19 Pandemic. *Int J Environ Res Public Health*, 19(23), 2022.

[8] M. Arafa, S. Nesar, H. Abu-Jabeh, M. O. R. Jayme, and Y. Kalairajah. COVID-19 pandemic and hip fractures: impact and lessons learned. *Bone and Joint Open*, 1(9):530–540, 2020.

[9] A. R. Arantes and V. Vieira. Did Ambulatory Surgical Units succeed in adapting to COVID-19 pandemic? The experience of a Portuguese Hospital with Ambulatory Surgery. *Ambulatory Surgery*, 27(2):39–43, 2021.

[10] R. A. Arisz, P. Meijer, N. C. V. Péquériaux, S. J. van de Leur, M. V. Lukens, M. P. M. de Maat, and M. J. Hollestelle. Impact of COVID-19 pandemic on the quality of test output in haemostasis laboratories. *International Journal of Laboratory Hematology*, 44(2):407–413, 2022.

[11] C. C. Bartenschlager, S. Temizel, A. Ebigbo, V. Gruenherz, P. Gastmeier, H. Messmann, J. O. Brunner, and C. Römmele. A Simulation-Based Cost-Effectiveness Analysis of Severe Acute Respiratory Syndrome Coronavirus 2 Infection Prevention Strategies for Visitors of Healthcare Institutions. *Value in Health*, 25(11):1846–1852, 2022.

[12] A. Belfiore, G. Centonze, P. Maisonneuve, C. Riva, D. Morelli, A. Mangogna, G. Sabella, G. Pruneri, and M. Milione. Covid-19 pandemic: Huge stress test for health system could be a great opportunity to update the workflow in a modern surgical pathology. *Cancers*, 13(13), 2021.

[13] R. A. Blauenfeldt, J. N. Hedegaard, C. Kruuse, D. Gaist, T. Wienecke, B. Modrau, D. Damgaard, S. P. Johnsen, G. Andersen, and C. Z. Simonsen. Quality in stroke care during the early phases of the COVID-19 pandemic: A nationwide study. *European Stroke Journal*, 8(1):268–274, 2023.

[14] V. Brunetti, A. Broccolini, P. Caliandro, R. Di Iorio, M. Monforte, R. Morosetti, C. Piano, F. Pilato, S. Bellavia, J. Marotta, I. Scala, A. Pedicelli, M. A. Pennisi, A. Caricato, C. Roberti, M. C. Altavista, A. Valenza, M. Distefano, E. Cecconi, M. Fanella, S. Roncacci, M. Tasillo, P. Calabresi, G. Frisullo, and G. D. Marca. Effect of the COVID-19 pandemic and the lockdown measures on the local stroke network. *Neurological Sciences*, 42(4):1237–1245, 2021.

[15] C. W. Böing, N. J. Froböse, F. Schaumburg, and S. Kampmeier. Impact of the COVID-19 Pandemic on the Management of Staphylococcus aureus Bloodstream Infections in a Tertiary Care Hospital. *Pathogens*, 12(4), 2023.

[16] P. Caldas and M. Varela. The Portuguese Public Hospitals Performance Evolution before and during the SARS-CoV-2 Pandemic (2017-2022). *SUSTAINABILITY*, 15(15), 2023.

[17] J. M. Casas-Rojo, P. S. Ventura, J. M. Antón Santos, A. O. de Latierro, J. C. Arévalo-Lorido, M. Mauri, M. Rubio-Rivas, R. González-Vega, V. Giner-Galvañ, B. Otero Perpiñá, E. Fonseca-Aizpuru, A. Muiño, E. Del Corral-Beamonte, R. Gómez-Huelgas, F. Arnalich-Fernández, M. Llorente Barrio, A. Sancha-Lloret, I. Rábago Lorite, J. Loureiro-Amigo, S. Pintos-Martínez, E. García-Sardón, A. Montaño-Martínez, M. G. Rojano-Rivero, J. M. Ramos-Rincón, and A. López-Escobar. Improving prediction of COVID-19 mortality using machine learning in the Spanish SEMI-COVID-19 registry. *Internal and emergency medicine*, 18(6):1711–1722, 2023.

[18] A. Chaddad and C. Tanougast. CNN Approach for Predicting Survival Outcome of Patients With COVID-19. *IEEE INTERNET OF THINGS JOURNAL*, 10(15):13742–13753, 2023.

[19] J. Correia, A. Ponte, C. Gomes, E. Afecto, M. M. Estevinho, A. Rodrigues, R. Pinho, and J. Carvalho. Readjustment of Capsule Endoscopy Protocols to the COVID-19 Pandemic in a Portuguese Tertiary Center. *Turkish Journal of Gastroenterology*, 33(7):570–575, 2022.

[20] G. Crozier-Shaw, A. J. Hughes, B. Conlon, E. Sheehan, and K. Merghani. Hip fracture care during Covid-19: a regional trauma centre's experience. *Irish Journal of Medical Science*, 190(4):1275–1280, 2021.

[21] S. J. Dauncey, P. A. Kelly, D. Baykov, A. C. Skeldon, and M. B. Whyte. Rhythmicity of patient flow in an acute medical unit: relationship to hospital occupancy, 7-day working and the effect of COVID-19. *Qjm*, 114(11):773–779, 2022.

[22] J. M. De Abreu, I. Cabo, M. I. Borges, A. Quiterio, T. Nunes, F. Matos, F. Vale, and J. Figueiredo. The COVID-19 Impact on Oral Healthcare Demand and Performance: The Experience of a Clinical and Academic Centre in Portugal (EU). *Acta Medica Portuguesa*, 36(1):15–24, 2023. Place: Portugal.

[23] L. Domingo, M. Comas, A. Jansana, J. Louro, H. Tizón-Marcos, M. L. Cos, J. Roquer, J. J. Chillarón, I. Cirera, S. Pascual-Guàrdia, M. Sala, and X. Castells. Impact of COVID-19 on Hospital Admissions and Healthcare Quality Indicators in Non-COVID Patients: A Retrospective Study of the First COVID-19 Year in a University Hospital in Spain. *Journal of Clinical Medicine*, 11(7), 2022.

[24] V. D'Ovidio, C. Lucidi, G. Bruno, D. Lisi, L. Miglioresi, and M. E. Bazuro. Impact of COVID-19 Pandemic on Colorectal Cancer Screening Program. *CLINICAL COLORECTAL CANCER*, 20(1):E5–E11, 2021.

[25] R. Doyle, S. Bate, J. Devenney, S. Agwaonye, M. Hastings, J. Wych, S. Archbold, and D. H. Vasant. Evaluating the impact of an enhanced triage process on the performance and diagnostic yield of oesophageal physiology studies post COVID-19. *BMJ Open Gastroenterology*, 8(1), 2021.

[26] D. Duarte, C. Walshaw, and N. Ramesh. A comparison of time-series predictions for healthcare emergency department indicators and the impact of COVID-19. *Applied Sciences (Switzerland)*, 11(8), 2021.

[27] J. Dupraz, E. Zuercher, and I. Peytremann-Bridevaux. Impact of the COVID-19 pandemic on the quality of diabetes care: Insights from longitudinal data in Switzerland. *Primary Care Diabetes*, 17(4):408–410, 2023.

[28] M. A. Elleuch, A. Ben Hassena, M. Abdelhedi, and F. S. Pinto. Real-time prediction of COVID-19 patients health situations using Artificial Neural Networks and Fuzzy Interval Mathematical modeling. *APPLIED SOFT COMPUTING*, 110, 2021.

[29] S. Esmaily, C. C. Yau, D. Dwarakanath, J. Hancock, and V. Mitra. Impact of COVID-19 pandemic on key performance indicators in pancreatobiliary endoscopy: Prioritise, minimise risk, keep scoping and training. *Frontline Gastroenterology*, 12(4):288–292, 2021.

[30] Ann-Charlotte Falk, Carolin Nymark, Katarina E. Göransson, and Ann-Christin von Vogelsang. Missed nursing care in the critical care unit, before and during the COVID-19 pandemic: A comparative cross-sectional study. *Intensive & Critical Care Nursing*, 72:N.PAG–N.PAG, 2022. Place: Philadelphia, Pennsylvania.

[31] C. Fazekas, M. Zieser, B. Hanfstingl, J. Saretzki, E. Kunschitz, L. Zieser-Stelzhammer, D. Linder, and F. Matzer. Physician resilience and perceived quality of care among medical doctors with training in psychosomatic medicine during the COVID-19 pandemic: a quantitative and qualitative analysis. *BMC Health Services Research*, 24(1), 2024.

[32] O. Fersia, S. Bryant, R. Nicholson, K. McMeeken, C. Brown, B. Donaldson, A. Jardine, V. Grierson, V. Whalen, and A. MacKay. The impact of the COVID-19 pandemic on cardiology services. *Open Heart*, 7(2), 2020.

[33] D. Fluck, C. H. Fry, S. Rankin, A. Lewis, J. Robin, J. Rees, J. Finch, Y. Jones, G. Jones, J. Tudose, L. Taylor, and T. S. Han. Does the length of stay in hospital affect healthcare outcomes of patients without COVID-19 who were admitted during the pandemic? A retrospective monocentric study. *Internal and emergency medicine*, 17(5):1385–1393, 2022.

[34] G. Frisullo, V. Brunetti, R. Di Iorio, A. Broccolini, P. Caliandro, M. Monforte, R. Morosetti, C. Piano, F. Pilato, P. Calabresi, and G. Della Marca. Effect of lockdown on the management of ischemic stroke: an Italian experience from a COVID hospital. *Neurol Sci*, 41(9):2309–2313, 2020.

[35] M. J. Ghassemi-Rad, C. Dennehy, N. Lyons, M. T. Henry, M. P. Kennedy, ÉJ O'Reilly, and R. M. Connolly. The impact of the COVID-19 pandemic on the performance of the Rapid Access Lung Cancer Clinic. *Irish Journal of Medical Science*, 2024.

[36] H. Ghayoomi, E. Miller-Hooks, M. Tariverdi, J. Shortle, and T. D. Kirsch. Maximizing hospital capacity to serve pandemic patient surge in hot spots via queueing theory and microsimulation. *IISE Transactions on Healthcare Systems Engineering*, 13(4):314–332, 2023.

[37] Adrian Gheorghe, Camille Maringe, James Spice, Arnie Purushotham, Kalipso Chalkidou, Bernard Rachet, Richard Sullivan, and Ajay Aggarwal. Economic impact of avoidable cancer deaths caused by diagnostic delay during the COVID-19 pandemic: A national population-based modelling study in England, UK. *European Journal of Cancer*, 152:233–242, 2021.

[38] P. D. Giorgi, F. Villa, E. Gallazzi, A. Debernardi, G. R. Schirò, F. M. Crisà, G. Talamonti, and G. D'Aliberti. The management of emergency spinal surgery during the COVID-19 pandemic in Italy. *Bone Joint J*, 102-b(6):671–676, 2020.

[39] A. Gobbi, D. Manerba, R. Mansini, and R. Zanotti. Hybridizing adaptive large neighborhood search with kernel search: a new solution approach for the nurse routing problem with incompatible services and minimum demand. *INTERNATIONAL TRANSACTIONS IN OPERATIONAL RESEARCH*, 30(1):8–38, 2023.

[40] A. González-Cebrián, J. Borràs-Ferrís, J. P. Ordovás-Baines, M. Hermenegildo-Caudevilla, M. Climente-Marti, S. Tarazona, R. Vitale, D. Palací-López, J. F. Sierra-Sánchez, J. S. de la Fuente, and A. Ferrer. Machine-learning-derived predictive score for early estimation of COVID-19 mortality risk in hospitalized patients. *PLoS One*, 17(9 September), 2022.

[41] B. S. Gonçalves, E. Vieira, R. M. Lima, and J. Dinis-Carvalho. A systematized approach for reduction of medical appointment waiting list. *Production*, 32, 2022.

[42] P. Gonçalves, P. Ferrari, L. Crivelli, and E. Albanese. Model-informed health system reorganization during emergencies. *PRODUCTION AND OPERATIONS MANAGEMENT*, 32(5):1323–1344, 2023.

[43] A. Graf, C. H. Koh, G. Caldwell, J. Grieve, M. Tan, J. Hassan, K. Bakaya, H. J. Marcus, and S. E. Baldeweg. Quality in Clinical Consultations: A Cross-Sectional Study. *Clinics and Practice*, 12(4):545–556, 2022.

[44] S. Griewing, N. Gremke, J. Kreutz, B. Schieffer, L. Timmermann, and B. Markus. Chronological Development of Cardiovascular Disease in Times of COVID-19: A Retrospective Analysis of Hospitalized Diseases of the Circulatory System and COVID-19 Patients of a German University Hospital. *Journal of Cardiovascular Development and Disease*, 9(10), 2022.

[45] S. Griewing, U. Wagner, M. Lingenfelder, S. Heinis, B. Schieffer, and B. Markus. Impact of the COVID-19 Pandemic on Delivery of Gynecology and Obstetrics Services at a Maximum Care University Hospital in Germany. *Geburtshilfe und Frauenheilkunde*, 82(4):427–440, 2022.

[46] F. Grimm, A. Johansen, H. Knight, R. Brine, and S. R. Deeny. Indirect effect of the COVID-19 pandemic on hospital mortality in patients with hip fracture: A competing risk survival analysis using linked administrative data. *BMJ Quality and Safety*, 32(5):264–273, 2023.

[47] S. Gruttadauria. Preliminary Analysis of the Impact of the Coronavirus Disease 2019 Outbreak on Italian Liver Transplant Programs. *Liver Transplantation*, 26(7):941–944, 2020.

[48] M. B. B. Hansen, M. Groenvold, M. Adsersen, H. Jensen, E. H. Ibfelt, M. A. Petersen, M. A. Neergaard, H. Møller, and T. B. Olesen. Palliative care need screening and specialised referrals fell during the COVID-19 pandemic: a nationwide register-based study. *BMJ Supportive and Palliative Care*, 14(e1):E1380–E1387, 2024.

[49] C. O. Henriques and M. C. Gouveia. Assessing the impact of COVID-19 on the efficiency of Portuguese state-owned enterprise hospitals. *SOCIO-ECONOMIC PLANNING SCIENCES*, 84, 2022.

[50] J. Horan, J. C. Duddy, B. Gilmartin, M. Amoo, D. Nolan, P. Corr, M. B. Husien, and C. Bolger. Neurotrauma admissions and COVID-19: a National Centre experience. *Irish Journal of Medical Science*, 191(4):1871–1876, 2022.

[51] B. Jamshidi, S. J. Zargaran, H. Bekrizadeh, M. Rezaei, and F. Najafi. Comparing length of hospital stay during COVID-19 pandemic in the USA, Italy and Germany. *International Journal for Quality in Health Care*, 33(1), 2021.

[52] S. Jidkova, S. Hoeck, E. Kellen, S. le Cessie, and M. C. Goossens. Flemish population-based cancer screening programs: impact of COVID-19 related shutdown on short-term key performance indicators. *BMC Cancer*, 22(1):183, 2022.

[53] J. Joseph, R. A. Kotronias, T. Estrin-Serlui, T. J. Cahill, R. K. Kharbanda, J. D. Newton, C. Grebenik, S. Dawkins, and A. P. Banning. Safety and Operational Efficiency of Restructuring and Redeploying a Transcatheter Aortic Valve Replacement Service During the COVID-19 Pandemic: The Oxford Experience. *Cardiovascular Revascularization Medicine*, 31:26–31, 2021.

[54] Q. Kong, D. Riedewald, and M. Askari. Factors affecting portal usage among chronically ill patients during the COVID-19 pandemic in the Netherlands: Cross-sectional study. *JMIR HUMAN FACTORS*, 8(3), 2021.

[55] T. Kuosmanen, Y. Tan, and S. Dai. Performance analysis of English hospitals during the first and second waves of the coronavirus pandemic. *Health Care Management Science*, 26(3):447–460, 2023.

[56] L. L. Liang, C. H. Tseng, H. J. Ho, and C. Y. Wu. Covid-19 mortality is negatively associated with test number and government effectiveness. *Scientific reports*, 10(1), 2020.

[57] G. Lo Moro, N. Marengo, A. Mara, J. R. Paño Pardo, S. Hernandez, E. Fusté, M. Pujol, C. M. Zotti, E. Limón, and C. Vicentini. Antimicrobial stewardship programs in acute-care hospitals: A multicenter assessment of structure, process, and outcome indicators in Italy and Spain. *Journal of Infection and Public Health*, 17(7), 2024.

[58] A. Losurdo, A. V. E. Lisa, M. Tomatis, A. Ponti, S. Montemezzi, E. Bonzano, and L. Fortunato. Highly specialized Breast Centers did not experience delay of care during COVID-19 pandemic in Italy: the Senonetwork experience. *Breast Cancer Res Treat*, 196(1):87–95, 2022.

[59] S. Luthra, G. Hunduma, M. Navaratnarajah, P. G. Malvindi, N. Goddard, S. Miskolczi, and T. Velissaris. Impact of COVID-19 on operation room utilization efficiency and cardiac surgery care pathway: single centre experience. *JOURNAL OF HOSPITAL MANAGEMENT AND HEALTH POLICY*, 7, 2023.

[60] A. López-Cheda, M. A. Jácome, R. Cao, and P. M. De Salazar. Estimating lengths-of-stay of hospitalised COVID-19 patients using a non-parametric model: a case study in Galicia (Spain). *Epidemiology and Infection*, 149, 2021.

[61] Y. Manla, F. A. Badarin, N. Bader, T. Lee-St John, M. R. Mehra, and F. Bader. Worldwide and Country-Specific Impact of the COVID-19 Pandemic on Heart Transplantation Volumes: A Longitudinal Analysis of 2020 and 2021. *Curr Probl Cardiol*, 48(11):101870, 2023.

[62] C. Mattiuzzi, G. Lippi, and B. M. Henry. Healthcare indicators associated with COVID-19 death rates in the European Union. *Public Health*, 193:41–42, 2021.

[63] E. Maury, M. O. Boldi, G. Greub, V. Chavez, K. Jaton, and O. Opota. An Automated Dashboard to Improve Laboratory COVID-19 Diagnostics Management. *Frontiers in Digital Health*, 3, 2021.

[64] S. Meakin and S. Funk. Quantifying the impact of hospital catchment area definitions on hospital admissions forecasts: COVID-19 in England, September 2020-April 2021. *BMC Medicine*, 22(1), 2024.

[65] G. J. Melman, A. K. Parlikad, and E. A. B. Cameron. Balancing scarce hospital resources during the COVID-19 pandemic using discrete-event simulation. *Health Care Management Science*, 24(2):356–374, 2021.

[66] J. K. Mortensen, R. A. Blauenfeldt, J. N. Hedegaard, C. M. Wejse, S. P. Johnsen, G. Andersen, and C. Z. Simonsen. Prevalence and impact of SARS-CoV-2 infection among patients with acute ischaemic stroke: a nationwide register-based cohort study in Denmark. *BMJ Open*, 14(3), 2024.

[67] C. P. Mutch, D. A. Ross, A. Bularga, R. N. R. Cave, M. E. Chase-Topping, A. Anand, N. L. Mills, O. Koch, C. L. Mackintosh, and M. R. Perry. Performance status: A key factor in predicting mortality in the first wave of COVID-19 in South-East Scotland. *Journal of the Royal College of Physicians of Edinburgh*, 52(3):204–212, 2022.

[68] G. Nattino, M. M. Paganuzzi, G. I. Ghilardi, G. Costantino, C. Rossi, F. Cortellaro, R. Cosentini, S. Paglia, M. Migliori, and G. Bertolini. Strategies to convert hospital beds for COVID-19 patients to minimize emergency department overcrowding. *Health Services Management Research*, 2023.

[69] A. M. Nunes and D. F. D. Ferreira. Evaluating Portuguese Public Hospitals Performance: Any Difference before and during COVID-19? *SUSTAINABILITY*, 15(1), 2023.

[70] L. Palazzo and R. Ievoli. Detecting Regional Differences in Italian Health Services during Five COVID-19 Waves. *STATS*, 6(2):506–518, 2023.

[71] C. Park, K. Sugand, A. Aframian, C. Morgan, N. Pakroo, C. Gibbons, M. Fertleman, D. Nathwani, R. Bhattacharya, and K. M. Sarraf. Impact of COVID-19 pandemic on hip fractures: the central London experience COVID-related urgent geriatric hip trauma (COUGH) study COVERT (COVid Emergency-Related Trauma and orthopaedics) collaborative. *Irish Journal of Medical Science*, 191(3):1005–1012, 2022.

[72] A. B. Pedersen, N. Risbo, B. L. Viberg, H. Palm, and N. Dieter Röck. Incidence, Quality of Care, and Mortality in Hip Fracture Patients in the Course of the COVID Pandemic: A Population-Based Cohort Study. *Journals of Gerontology - Series A Biological Sciences and Medical Sciences*, 79(4), 2024.

[73] N. Penel, C. Cantarel, C. Chemin-Airiau, F. Ducimetiere, F. Gouin, F. Le Loarer, M. Toulmonde, S. Piperno-Neumann, C. Bellera, C. Honore, J. Y. Blay, and S. Mathoulin-Pelissier. Impact of the coronavirus disease 2019 pandemic on sarcoma management in France: a 2019 and 2020 comparison. *Therapeutic Advances in Medical Oncology*, 15, 2023.

[74] A. M. Ponsiglione, M. Romano, and F. Amato. A Finite-State Machine Approach to Study Patients Dropout from Medical Examinations. *6th International Forum on Research and Technology for Society and Industry, RTSI 2021 - Proceedings*, pages 289–294, 2021.

[75] T. Quibel, N. Winer, L. Bussieres, C. Vayssiere, P. Deruelle, M. Defrance, P. Rozenberg, J. Bouyer, N. Dupuis, B. Renaudin, L. Dugave, N. Banaszkiewicz, C. Garabedian, and Y. Ville. Impact of COVID-19-Related Lockdown on Delivery and Perinatal Outcomes: A Retrospective Cohort Study. *Journal of Clinical Medicine*, 11(3), 2022.

[76] L. Quinn, P. Bird, and R. Lilford. Effect of cancer waiting time standards in the English National Health Service: a threshold analysis. *BMC Health Services Research*, 24(1), 2024.

[77] A. Ramírez Calazans, R. M. Paredes Esteban, O. B. Grijalva Estrada, and M. R. Ibarra Rodríguez. Assessment of quality indicators in pediatric major outpatient surgery. Influence of the COVID-19 pandemic. *Cirugia pediatrica : organo oficial de la Sociedad Espanola de Cirugia Pediatrica*, 36(1):17–21, 2023.

[78] N. Reidy, H. Coetzee, C. Roche, E. Brazil, L. O'Sullivan, D. Brady, and M. Lynch. SARS-CoV-2 Testing and Patient Waiting Times in the Emergency Department. *Irish medical journal*, 115(7):633, 2022.

[79] K. H. Scholz, B. Lengenfelder, C. Thilo, A. Jeron, S. Stefanow, U. Janssens, J. Bauersachs, P. C. Schulze, K. D. Winter, J. Schröder, J. vom Dahl, N. von Beckerath, K. Seidl, T. Friede, and T. Meyer. Impact of COVID-19 outbreak on regional STEMI care in Germany. *Clinical Research in Cardiology*, 109(12):1511–1521, 2020.

[80] A. Sedghi, T. Siepmann, L. P. Pallesen, H. Reichmann, V. Puetz, J. Barlinn, and K. Barlinn. Delayed comprehensive stroke unit care attributable to the evolution of infection protection measures across two consecutive waves of the covid-19 pandemic. *Life*, 11(7), 2021.

[81] E. F. Sánchez-Ubeda, P. Sánchez-Martín, M. Torrego-Ellacuría, A. Del Rey-Mejías, M. F. Morales-Contreras, and J. L. Puerta. Flexibility and Bed Margins of the Community of Madrid's Hospitals during the First Wave of the SARS-CoV-2 Pandemic. *International Journal of Environmental Research and Public Health*, 18(7), 2021.

[82] G. Terning, E. C. Brun, and I. El-Thalji. Modeling Patient Flow in an Emergency Department under COVID-19 Pandemic Conditions: A Hybrid Modeling Approach. *Healthcare (Switzerland)*, 10(5), 2022.

[83] D. Tricarico, M. Calandri, M. Barba, C. Piatti, C. Geninatti, D. Basile, M. Gatti, M. Melis, and A. Veltri. Convolutional Neural Network-Based Automatic Analysis of Chest Radiographs for the Detection of COVID-19 Pneumonia: A Prioritizing Tool in the Emergency Department, Phase I Study and Preliminary "Real Life" Results. *Diagnostics*, 12(3), 2022.

[84] T. A. Trunfio and G. Improta. Flow Analysis of Mastectomy Patients Using Length of Stay: A Single-Center Study. *BioMedInformatics*, 4(3):1725–1744, 2024.

[85] Y. Tung-Chen, A. Gil-Rodrigo, A. Algora-Martín, R. Llamas-Fuentes, P. Rodríguez-Fuertes, R. Marín-Baselga, B. Alonso-Martínez, E. S. Rodríguez, P. L. Soriano, and J. M. Ramos-Rincón. The lung ultrasound ”Rule of 7” in the prognosis of COVID-19 patients: Results from a prospective multicentric study. *Medicina Clinica*, 159(1):19–26, 2022.

[86] I. Vagliano, S. Brinkman, A. Abu-Hanna, M. S. Arbous, D. A. Dongelmans, P. W. G. Elbers, D. W. de Lange, M. van der Schaar, N. F. de Keizer, and M. C. Schut. Can we reliably automate clinical prognostic modelling? A retrospective cohort study for ICU triage prediction of in-hospital mortality of COVID-19 patients in the Netherlands. *International Journal of Medical Informatics*, 160:N.PAG–N.PAG, 2022. Place: New York, New York.

[87] Davide Valsecchi, Luca Porcu, Abdelrahman Khater, Rosa Alessia Battista, Leone Giordano, Stefano Cascinu, Andrea Assanelli, Chiara Lazzari, Vanesa Gregorc, and Aurora Mirabile. Emergency Department Visits among Cancer Patients during SARS-CoV-2 Pandemic. *Cancers*, 15(4):1240, 2023.

[88] P. van Dam, M. Tomatis, A. Ponti, L. Marotti, C. Aristei, L. Biganzoli, M. J. Cardoso, K. L. Cheung, G. Curigliano, J. De Vries, D. Santini, F. Sardanelli, and I. T. Rubio. The impact of the SARS-COV-2 pandemic on the quality of breast cancer care in EUSOMA-certified breast centres. *Eur J Cancer*, 177:72–79, 2022.

[89] S. Vanni, P. Bartalucci, U. Gargano, A. Coppa, G. Giannasi, P. Nazerian, B. Tonietti, R. Vannini, M. Lanigra, F. Daviddi, A. Baldini, S. Grifoni, and S. Magazzini. The presentations/physician ratio predicts door-to-physician time but not global length of stay in the emergency department: an Italian multicenter study during the SARS-CoV-2 pandemic. *Internal and emergency medicine*, 17(3):829–837, 2022.

[90] J. L. Waterman, U. Jayaraju, J. K. Nadimi, and D. Morgan. Impact of COVID-19 on Key Performance Indicators of the National Hip Fracture Database and the Management of Hip Fracture Patients. *CUREUS JOURNAL OF MEDICAL SCIENCE*, 13(12), 2021.

[91] P. E. Wikman-Jorgensen, A. Ruiz, V. Giner-Galvan, J. Llenas-Garcia, J. M. Segui-Ripoll, J. M. Salinas Serrano, E. Borrajo, J. M. Ibarra Sanchez, J. P. Garcia-Sabater, and J. A. Marin-Garcia. Hospitalization forecast to inform COVID-19 pandemic planning and resource allocation using mathematical models. *medRxiv*, 2022. Place: United States.

[92] R. M. Wood. Modelling the impact of COVID-19 on elective waiting times. *JOURNAL OF SIMULATION*, 16(1):101–109, 2022.

[93] R. M. Wood, C. J. McWilliams, M. J. Thomas, C. P. Bourdeaux, and C. Vasilakis. COVID-19 scenario modelling for the mitigation of capacity-dependent deaths in intensive care. *Health Care Management Science*, 23(3):315–324, 2020.

[94] E. Woodland and M. Carroll. Improving ICSI success rates following root cause analysis and use of system behaviour charts: the devil is in the detail! *BMJ Open Quality*, 11(4), 2022.
