# Supplementary material for: Performance indicators for evaluating and optimising hospital services during COVID-19: a scoping review
Source: BMJ Open. 2026 Sep 28;16(9):e122362. doi: 10.1136/bmjopen-2026-122362 (PMC13630113; doi:10.1136/bmjopen-2026-122362)
Supplement: Supplementary file 2 [file bmjopen-16-9-s003.pdf]

Appendix C: Key performance indicators for evaluating and optimising hospital services during COVID-19: complete list of KPIs

* Empty cells indicate that no definition or description of the KPI was provided in the article.

KPI	Definition of KPI*	Medical specialty	Study design
**A1. Mortality**			
30-day mortality rate [8]		Orthopedics (surgical), Trauma	Observational
Death [14]		Neurology	Observational
In-hospital mortality [17]		Infectious diseases	Modelling
Survival outcome [18]		Infectious diseases	Modelling
30-day mortality [20]		Orthopedics (surgical)	Observational
In-hospital mortality rate [23]	Percentage of deaths among hospitalised patients	Hospital-wide	Observational
Survival rate [28]		Infectious diseases	Modelling
30 day after procedure-mortality [29]	Measured in percentage	Gastroenterology	Observational
Mortality within 30 days of discharge [33]		Hospital-wide	Observational
Mortality in hospital [33]		Hospital-wide	Observational
Death rate [34]	Deaths out of total number of admissions.	Neurology	Observational
Daily hospital deaths [42]	Daily total number of COVID-19-deaths.	Infectious diseases	Modelling
Overall inpatient mortality [46]	Number of patients that dies in the hospital divided by the total number of patients.	Orthopedics	Observational
Mortality patients without concomitant COVID-19 [46]		Orthopedics	Observational
Daily risk of hospital death [46]		Orthopedics	Observational
30-day mortality rate [50]		Trauma	Observational
30-day mortality or readmission [53]	30-day mortality in TAVR (Transcatheter Aortic Valve Replacement) patients after 30 days following TAVR.	Cardiovascular surgery	Observational
In-hospital mortality rate [53]	In-hospital deaths of patients following TAVR.	Cardiovascular surgery	Observational

KPI	Definition of KPI*	Medical specialty	Study design
Inpatient mortality [55]	Definition is not explicit, but it is calculated using the average number of beds occupied by the confirmed COVID-19 patients during a week; the number of COVID-19 discharges during a week; and the number of COVID-19 deaths during a week. Is also split up in 3 age-categories.	Infectious diseases	Observational,Modelling
Mortality [56]	Total number of COVID-19 cases divided by total number of COVID-19 deaths.	Infectious diseases	Observational
COVID-19 mortality rate [62]	In %	Infectious diseases	Observational
Mortality [40]	Binary variable 'mortality' with levels 'alive' (numerically as zero) or 'deceased' (numerically as one). Baseline was the date of hospital admission. The follow-up censoring date was July 15th, 2020; if a patient had not reached the outcome (death) by the time the data were obtained, their outcome was considered as null.	Infectious diseases	Modelling, Observational
Deaths [65]	Including non-COVID-19 deaths; specifically surgical patients not surviving their hospital stay.	Hospital-wide	Modelling, Observational
30 day mortality [66]	Mortality within 30 days after stroke admission	Not specified	Observational
Mortality rate [67]	In-patient mortality	Not specified	Modelling
Number of patients died at hospital [67]	Number of COVID patients died at the hospital	Hospital-wide	Modelling
1-year mortality rate [67]	Mortality rate of COVID patients after 1 year of hospital admission.	Hospital-wide	Modelling
Number of COVID-19 patients deceased in hospital [70]	Daily number of COVID-19 confirmed patients that died in the hospital.	ICU	Observational
30-day mortality [72]	The number of people who died within 30 days of surgery. Date of death was obtained from the Civil Registration System and updated on daily basis. Also, 30-day mortality was divided into in-hospital and posthospital mortality.	Emergency department (ED)	Observational
In-hospital mortality [72]		Emergency department (ED)	Observational
Neonatal mortality [75]	Number of newborn babes died	Obstetrics	Observational
Number of stillbirths [75]		Obstetrics	Observational
Hospital mortality [79]		Cardiology	Observational
Mortality of COVID-19 patient [85]		Emergency department (ED)	Modelling

KPI	Definition of KPI*	Medical specialty	Study design
In-hospital mortality [86]	Percentage of COVID-19 patients that die in the hospital	ICU	Modelling, Observational
Deaths [87]	Number of ED visits by oncology patients with outcome 'death'.	Emergency department (ED)	Observational
Mortality at ED [89]	Number of patients died in the ED	Emergency department (ED)	Observational
30-day mortality [90]	30-day postoperative patient mortality	Orthopedics (surgical)	Observational
**A2. Individual outcomes**			
NIHSS score 24 hours after admission [5]	Quantifies stroke severity based on weighted evaluation findings.	Neurology	Observational
Return to original residence by 120 days [8]	Percentage of patients known to have returned to their original residence by 120 days after their hip fracture.	Orthopedics (surgical), Trauma	Observational
NIHSS at the onset and discharge [14]		Neurology	Observational
Modified Rankin scale at discharge [14]		Neurology	Observational
Severity rate [23]	Percentage of episodes with maximum severity (4). Severity levels were obtained from the APR-DRG classification, consisting of values between 1 and 4, with one being the least severe category. Severity comparisons are allowed within the same APR-DRG only.	Hospital-wide	Observational
HbA1c value [27]		Endocrinology	Observational
PACIC score [27]		Endocrinology	Observational
ADDQoL score [27]		Endocrinology	Observational
NIHSS at admission [34]	Quantifies stroke severity based on weighted evaluation findings.	Neurology	Observational
NIHSS at discharge [34]	Quantifies stroke severity based on weighted evaluation findings.	Neurology	Observational
Record of histological type, grading, hormonal status, HER2 status, margins, vascular invasion and size for invasive forms [58]		Oncology	Observational
Record of histological type, grading, hormonal status, margins and size for non-invasive forms [58]		Oncology	Observational
Stroke severity (score based) [66]	The scale ranges from 0 to 58, where 58 corresponds to normal neurological function. Severe stroke was defined as an SSS score < 30.	Not specified	Observational
Gestational age at delivery [75]	The number of weeks a pregnancy has lasted when a baby is born, calculated from the first day of the mother's last menstrual period.	Obstetrics	Observational

KPI	Definition of KPI*	Medical specialty	Study design
Mode of labor onset [75]	Percentage of patients that had spontaneous labor onset, induction, or prelabor Caesarean delivery.	Obstetrics	Observational
Delivery mode [75]	Percentage of patients that had spontaneous vaginal deliverry, operative vaginal, or Caesarean delivery.	Obstetrics	Observational
1- and 5-min Apgar score [75]		Obstetrics	Observational
Umbilical artery pH score [75]		Obstetrics	Observational
Breast cancer EUSOMA quality indicators [88]	EUSOMA quality indicators that were assessed in the present analysis: 1) Cancers with a pre-operative diagnosis (B5 or C5); 2) Invasive ca with histological type; grading; ER/Her2; pN; margins; vascular invasion and size recorded; 3) Non-invasive ca with histological pattern; grading; size; margins and ER recorded; 4) M0 invasive ca receiving postoperative RT after BCT; 5) Invasive ca 3 cm (incl. DCIS component) treated with BCT; 6) Non-invasive ca 2 cm treated with BCT; 7) DCIS with no axillary clearance; 8) Endocrine sensitive invasive ca receiving HT; 9) ER- (T > 1 cm or N) invasive ca receiving CT; 10) Invasive ca receiving just 1 operation (excl. reconstruction); 11) DCIS receiving just 1 operation (excl. reconstruction); 12 SLNB in cN0 invasive ca (without neoadjuvant); 13) Immediate reconstruction after mastectomy; 14) No more than 5 nodes excised in invasive ca with SLNB; 15) Invasive Her2+ (T > 1 cm or N+) with adjuvant chemotherapy who received adjuvant biological drug.	Oncology	Observational
**A3. Readmissions and reoperations**			
Inpatient readmission [3]	Number of inpatients not being unexpectedly readmitted within 30 days since discharge.	Hospital-wide	Modelling, Observational
Reoperation rate [8]		Orthopedics (surgical), Trauma	Observational
Reintervention rate [9]	Number of ambulatory patients who underwent reintervention within the first 48h, as ambulatory patient again.	General surgery	Observational
Readmission rate [9]	Number of patients operated at the ambulatory surgery unit that were admitted to the hospital in the first 48h after discharge.	General surgery	Observational

KPI	Definition of KPI*	Medical specialty	Study design
Readmission rate [23]	Percentage of readmissions among hospitalised patients. Readmissions were defined as episodes with urgent admission within 30 days of discharge from an earlier clinically related hospitalisation and could be due to disease recurrence, worsening of a chronic condition, or complications from the previous episode.	Hospital-wide	Observational
Readmissions within 28 days of discharge [33]		Hospital-wide	Observational
Number of readmitted cases after 30 days of inpatient discharge [69]	Number of readmitted cases after 30 days of inpatient discharge. The inpatient was inappropriately discharged or was not correctly informed about home care. Generally, the inpatient is in a more severe and complex clinical scenario when readmitted than was during the first admission and, for that reason, demands more resources.	Hospital-wide	Modelling
Readmission rate [77]	Measures the number of patients who were discharged after surgery under the MOS system and who required readmission in the hours or days following surgery due to some post-surgical complication.	Pediatric surgery	Observational
Readmission to the ICU [86]		ICU	Modelling, Observational
Number of return-to-ED within 72 h [89]	Number of patients that returned to the ED for another visit within 72 h. Return to visit within 72 h included unscheduled patients who returned to ED for any reason within 72 h after the index visit.	Emergency department (ED)	Observational
**A4. Clinical complications**			
Bloodstream infections [3]	Number of bloodstream infection provoked by a central venous catheter	Hospital-wide	Modelling, Observational
Decubitus ulcers [3]	Number of decubitus ulcers in admitted patients	Hospital-wide	Modelling, Observational
Postoperative sepsis [3]	Number of septicemia cases after surgery	Hospital-wide	Modelling, Observational
Postoperative deep vein thrombosis of pulmonary embolisms [3]	Number of PE and DVT after surgery	Hospital-wide	Modelling, Observational
Occurrence of symptomatic intracranial hemorrhage (sICH) [5]	Defined by the Second European-Australasian Acute Stroke Study	Neurology	Observational
Wound infection rate [8]		Orthopedics (surgical), Trauma	Observational
Additional COVID-infections [11]	Additional in-hospital COVID-infections	Hospital-wide	Modelling

KPI	Definition of KPI*	Medical specialty	Study design
Pneunomia [14]	Number of stroke patients that got pneunomia	Neurology	Observational
COVID-19	Number of stroke patients that got COVID-19	Neurology	Observational
Rate of complications [19]		Gastroenterology	Observational
Pressure ulcers [20]	Percentage of patients that develops pressure ulcers	Orthopedics (surgical)	Observational
Complication rate [23]	Percentage of complications among hospitalised patients	Hospital-wide	Observational
Post-ERCP complications [29]	Post-ERCP sphincterotomy bleed, post-ERCP cholangitis, measured in percentages	Gastroenterology	Observational
Fever during hospitalization [34]		Neurology	Observational
Pneunomia during hospitalization [34]		Neurology	Observational
Percentage of MRSA over invasive isolates [57]		Infectious diseases	Observational
SARS-CoV-2 status [66]	Concurrent SARS-CoV-2 infection was defined as a positive PCR test within 31 days prior to stroke admission and until 1 day after admission.	Stroke specific	Observational
Number of avoidable bedsore cases [69]	Pressure ulcers are skin or underlying tissues injuries commonly found in low-mobility patients spending most of their in-hospital time laying on their beds. These injuries jeopardize the patient's safety.	Hospital-wide	Modelling
Number of avoidable bloodstream infections [69]	Bloodstream infections result from bacteremia present in not properly sterilized intravenous catheters, which is a leading cause of in-hospital mortality.	Hospital-wide	Modelling
Number of avoidable post-operative pulmonary embolisms [69]	Pulmonary embolism occurs mostly after a major surgery or a major injury, when a blood clot forms in a deep vein and breaks loose traveling through bloodstream to the lungs, resulting in several in-hospital preventable events including lack of oxygen in organs and tissues, and ultimately death. The literature suggests an increase of pulmonary embolism events and thromboembolisms because of the systemic coagulation activation and thrombotic complications resulting from COVID-19 infections.	Hospital-wide	Modelling

KPI	Definition of KPI*	Medical specialty	Study design
Number of septicemia cases [69]	Hospital-acquired infections by bacteria, viruses, or fungi, mostly because of poor materials or cleanliness, are called septicemia or nosocomial infections, and seriously compromise the patients' safety during their stay in the hospital ward. During the COVID-19 this type of infections may have increased because of ventilator-associated pneumonia and bacteremia.	Hospital-wide	Modelling
Occurrences of vaginal perineal tear [75]	Number of patients that had vaginal perineal tear	Obstetrics	Observational
Severe postpartum hemorrhage [75]	Number of patients that experienced severe postpartum hemorrhage, defined as blood loss exceeding 1L.	Obstetrics	Observational
Maternal blood transfusion [75]	Number of patients that underwent maternal blood transfusion.	Obstetrics	Observational
**B1. Number of inpatients**			
Hospitalized ICU [42]	Daily total number of COVID-19-patients on ICUs in country.	Infectious diseases	Modelling
Hospitalized ward [42]	Daily total number of COVID-patients on wards (countrywide).	Infectious diseases	Modelling
Montly case number inpatients and outpatients [45]	Number of cases per month.	Gynaecology/Obstetrics (incl. reproductive care)	Observational
Number of hospitalization required (COVID-19) patients [47]	Required hospitalization COVID-19 infection in transplant recipients.	Transplantation	Observational
Average weekly bed occupancy during the busiest week [55]		Infectious diseases	Observational,Modelling
Number of COVID inpatients [67]	Hospital acquired COVID-19 defined as new symptoms and a SARS-CoV-2 RT-PCR positive $\geq$ 8days following admission.	Not specified	Modelling
Number of standard patients [69]	Total number of patients (both inpatients and outpatients) seen in the hospital, adjusted for their complexity and severity to become equivalent (standard). This variable can be seen as an aggregated hospital activity and production measure. It makes the comparison between hospitals under different conditions fairer as it is done on a common (national) basis.	Hospital-wide	Modelling
Number of patients treated in the ICU [70]	Daily number of COVID-19 confirmed patients that are hospitalized in the ICU.	ICU	Observational
Number of patients in the hospital wards [69]	Daily number of COVID-19 confirmed patients that are hospitalized in a ward at the hospital.	ICU	Observational

KPI	Definition of KPI*	Medical specialty	Study design
SARS-CoV-2 positive patients [80]	Number of SARS-CoV-2 positive patients	Emergency department (ED)	Observational
Number of COVID-19 patients [83]	Number of COVID-19 positive patients being confirmed by an RT-PCR test within 24 hours.	Emergency department (ED)	Modelling
Number of COVID-19 patients [86]	Patients were considered to have COVID-19 when the RT-PCR of their respiratory secretions was positive for SARS-CoV-2 or when their CT-scan was consistent with COVID-19.	ICU	Modelling, Observational
Bed occupancy [91]	Number of occupied beds.	Not specified	Modelling
**B2. Number of beds**			
Number of beds [3]	Number of inpatient's service beds	Hospital-wide	Modelling, Observational
Number of beds [49]	Number of beds at 37 state-owned enterprises (SOE) hospitals.	Not specified	Modelling
Hospital beds [62]	Per 100.000 inhabitants	Infectious diseases	Observational
Acute care beds [62]	Per 100.000 inhabitants	Infectious diseases	Observational
Number of converted beds [68]	Number of beds that are converted from non-COVID-19 to COVID-19 beds.	Infectious diseases	Observational,Modelling
Beds [69]	Total beds used in all hospital wards. This variable is a standard input in most studies and can reflect the hospital size and investment in other equipment.	Hospital-wide	Modelling
ICU beds [81]	Number of total occupied ICU beds	Hospital-wide	Modelling
COVID-19 ICU beds [81]	Number of COVID-19-occupied ICU beds	Hospital-wide	Modelling
Non-ICU beds [81]	Number of total occupied non-ICU beds	Hospital-wide	Modelling
COVID-19 non-ICU beds [81]	Number of COVID-19-occupied non-ICU beds	Hospital-wide	Modelling
Bed margin [81]	Ratio of unoccupied beds to the total number of available beds, where the number of unoccupied beds is the difference between the number of available beds and the occupied beds.	Hospital-wide	Modelling
Bed flexibility indicators [81]	Estimation of the flexibility of the available beds in hospitals, which describes the ability to adapt to the growing demand for beds. Three new flexibility indicators were defined: -the Average Ramp Rate Until the Peak (ARRUP) (beds/day), -the Ramp Duration Until the Peak (RDUP) (days), and -the Ramp Growth Until the Peak (RGUP) (beds).	Hospital-wide	Modelling
Elective capacity [92]	Elective capacity relative to the number of treatments performed in 12 months (in %)	Not specified	Modelling

KPI	Definition of KPI*	Medical specialty	Study design
ICU beds [93]	Number of ICU beds at hospital	Emergency department (ED)	Modelling
**B3. Number of staff**			
Number of doctors [3]	Number of doctors' monthly regular and overtime hours	Hospital-wide	Modelling, Observational
Number of nurses [3]	Number of nurses' monthly regular and overtime hours	Hospital-wide	Modelling, Observational
Number of senior doctors [32]		Cardiology	Observational
Number of junior doctors [32]		Cardiology	Observational
Number of nursing staff [32]		Cardiology	Observational
Number of physicians [49]	Number of physicians at 37 state-owned enterprises (SOE) hospitals.	Not specified	Modelling
Number of nursing staff [49]	Number of nursing staff at 37 state-owned enterprises (SOE) hospitals.	Not specified	Modelling
Number of operational staff [49]	Number of operational staff at 37 state-owned enterprises (SOE) hospitals.	Not specified	Modelling
Staff absence [55]	Number of absent staff members divided by total number of staff members.	Infectious diseases	Observational,Modelling
Physicians [62]	Per 100.000 inhabitants	Infectious diseases	Observational
Nurses [62]	Per 100.000 inhabitants	Infectious diseases	Observational
Doctors working time [69]	Total number of hours (ordinary and extraordinary) worked by all medical doctors.	Hospital-wide	Modelling
Nurses working time [69]	Total number of hours (ordinary and extraordinary) worked by all nurses.	Hospital-wide	Modelling
Number of services [74]	Number of health services	Emergency department (ED)	Modelling
Presentations/physician ratio [89]	The number of daily presentations normalized for the number of emergency physicians on duty.	Emergency department (ED)	Observational
**B4. Utilization**			
Occupancy rate [6]	Percentage of time that OR is actively used for surgical procedures during its scheduled operating hours	Orthopedics (surgical), Trauma	Observational
Utilization [36]	Number of patients that flow in divided by number of patients that flow out.	Infectious diseases,ICU	Modelling
ICU occupancy [42]	Ratio between occupied and total available beds.	Infectious diseases	Modelling
Bed shortage [42]	Computed by subtracting the data on patients hospitalized (actual demand) from the 1-week delayed bed capacity.	Infectious diseases	Modelling
Ward occupancy [42]	Ratio between occupied and total available beds	Infectious diseases.	Modelling

KPI	Definition of KPI*	Medical specialty	Study design
Number of days with no operating (0 operations/day) [59]		Cardiovascular surgery	Observational
OR utilization capacity [59]		Cardiovascular surgery	Observational
Number of days with < 50% operating (< 3 operations per day) [59]		Cardiovascular surgery	Observational
Acute care bed occupancy [62]	In %	Infectious diseases	Observational
Time in use [82]	Percentage of (simulated) time resources are used.	Emergency department (ED)	Modelling
**B5. ED crowding**			
Patients in department [26]	Hourly average number of patients waiting to be seen in the ED.	Emergency department (ED)	Modelling
Unallocated patients with decision to admit [26]	Number of patients that have been attended, not discharged, but waiting to be admitted to the hospital, thus occupying resources.	Emergency department (ED)	Modelling
Patient attendances [26]	Hourly total number of patients with major injuries attending the ED.	Emergency department (ED)	Modelling
Medically fit for discharge [26]	Total number of patients who have attended ED, and do not need further treatment.	Emergency department (ED)	Modelling
ED crowding of COVID-19 patients [68]	Daily number of COVID-19 patients waiting for hospitalization at noon. This number was retrospectively computed by counting, for each hospital, the patients who were present in the ED at 12:00 p.m.and have been eventually hospitalized after the visit. Patients staying in the ED more than 36h from the start of the visit were considered hospitalized regardless of the ED outcome, to correctly classify the patients discharged at home after the treatments provided in the ED while waiting for a hospital bed.	Infectious diseases	Observational,Modelling

KPI	Definition of KPI*	Medical specialty	Study design
Patients' tolerance threshold [74]	Patient's tolerance threshold was defined based on the level of crowding calculated when the patient resides in the state q_0, i.e. when he/she is waiting for the medical examination. The following 3 different values of patient's tolerance threshold have been hypothesized and tested: 1) high tolerance threshold = 30: the number of patients waiting in the queue equals the triple of the maximum number of people allowed in the waiting room, which is assumed to be 10; 2) medium tolerance threshold = 20: the number of patients waiting in the queue equals the double of the maximum number of people allowed in the waiting room, which is assumed to be 10;3) low tolerance threshold = 10: the number of patients waiting in the queue equals the maximum number of people allowed in the waiting room, which is assumed to be 10.	Emergency department (ED)	Modelling
Crowding [82]	The number of patients simultaneously staying within the ED facility, i.e., prevalence. This measure tracks how long the crowding in the ED is above certain predefined levels. Measured in percentage per time.	Emergency department (ED)	Modelling
Peak crowding [82]	Number of patients present in the ED at the peak of the day.	Emergency department (ED)	Modelling
Time of peak [82]	Time of the day the max peak number of patients present in the ED is observed.	Emergency department (ED)	Modelling
Time full [82]	This measure keeps track of when the resource first reached its full capacity (i.e., there was no more left of the resource for a new patient for the first time).	Emergency department (ED)	Modelling
Number of times treatment room is blocked for contaminated patients [82]	Counter that keeps track of the number of times a patient with virus suspicion is blocked from going directly to a treatment room after undergoing the pre-triage screening.	Emergency department (ED)	Modelling
**B6. Efficiency scores**			

KPI	Definition of KPI*	Medical specialty	Study design
Composite indicator for efficiency and productivity [16]	Linear programming model that takes inputs (measured a certain point in time) and variables (weights) to maximize the sum of the products of those, which is then defined as the composite indicator (CI). $\text{CI} = \sum_i \text{weight}_i * \text{input}_i$. Input factors for efficiency and productivity are bed occupancy rate, number of patients per Full-Time Equivalent (FTE) doctor and number of patients per FTE nurse.	Hospital-wide	Modelling
Total Factor Productivity (TFP) for efficiency and productivity [16]	Calculated factor that relates the outcome of two CI measuring moments. It takes the optimal variables and the inputs of both CI linear programming models as input. Score > 1 indicates the hospital improved. Input factors for efficiency and productivity are bed occupancy rate, number of patients per Full-Time Equivalent (FTE) doctor and number of patients per FTE nurse.	Hospital-wide	Modelling
Hospital stress [21]	Two variables were considered each day: total medical inpatient occupancy; and the number of medical outliers at midnight at the beginning of the day in question.	Emergency department (ED),Hospital-wide	Observational
Index of operational efficiency (InOE) [59]	InOE (%) = 100–[(total OR time240)100]/240	Cardiovascular surgery	Observational
Anaesthetic efficiency (AE) [59]	AE (%) = 100–[(induction time + preparation time + sign out time)/total OR time]100	Cardiovascular surgery	Observational
Surgical efficiency (SE) [59]	SE (%) = (surgical time/total OR time)100	Cardiovascular surgery	Observational
Surgical index of operational efficiency (sInOE) [59]	sInOE (%) = 100–[(total OR time surgical time)100]/240	Cardiovascular surgery	Observational
**C1. Admissions**			
Emergency episodes [3]	Number of patients admitted to the emergency room	Hospital-wide	Modelling, Observational
Unexpected admission rate [9]	Number of unpredicted inpatient hospitalization (in a hospital ward, so not the ambulatory unit itself) from the ambulatory surgery unit.	General surgery	Observational
Total number of stroke patients admitted [14]		Neurology	Observational
Number of patients admitted through the ER [22]		Dentistry	Observational
Weekly number of admissions [23]	Hospital admissions	Hospital-wide	Observational
Number of ward admissions [32]	Admissions to cardiology ward/CCU, chest pain admissions, myocardial infarction admissions	Cardiology	Observational

KPI	Definition of KPI*	Medical specialty	Study design
Admission rate per specialty [33]	Number of admissions for a specialty divided by total number of admissions.	Hospital-wide	Observational
Number of COVID-admissions per day [36]		Infectious diseases,ICU	Modelling
Daily hospital admissions [42]	Number of COVID-19-admissions per day (countrywide).	Infectious diseases	Modelling
Number of short-term admissions [45]	Number of admissions with duration above the threshold length for admission but below the average length of admission.	Gynaecology/Obstetrics (incl. reproductive care)	Observational
Number of long-term admissions [45]	Number of admissions with duration above the average length of admission but below the upper threshold for length of admission.	Gynaecology/Obstetrics (incl. reproductive care)	Observational
Number of ICU admission required (COVID) patients [47]	Required ICU admission COVID-19 infection in transplant recipients.	Cardiovascular surgery	Observational
Number of admitted patients [48]	Number of admittances to specialised palliative care.	Observational	
ICU admissions [50]	Number of ICU admissions	Trauma	Observational
Admission rate [53]	Number of hospital visits	Cardiovascular surgery	Observational
Number of COVID admissions [64]		Not specified	Modelling
COVID-19 admissions [65]		Hospital-wide	Modelling, Observational
Number of ICU admission [67]	Requirement for intensive care admission (ICU) during the index episode and 1-year all-cause mortality.	Not specified	Modelling
Admission to neonatal intensive care [75]	Number of newborn babes admitted to neonatal intensive care unit.	Obstetrics	Observational
Hospitalization of COVID-19 patient [85]		Emergency department (ED)	Modelling
Number of admissions [87]	Number of patients admitted to hospital	Emergency department (ED)	Observational
Number of hospital admissions [89]	Number of hospital admissions (to hospital and to ED)	Emergency department (ED)	Observational
Total COVID-19 hospital admissions [91]	Number of total COVID-19 hospital admissions	Not specified	Modelling
COVID-19 ICU admissions [92]	Number of COVID-19 patients that had been admitted to intensive care at the hospital.	ICU	Modelling
**C2. Discharges**			
Inpatients treated [3]	Number of inpatient discharges	Hospital-wide	Modelling, Observational
Number of inpatient discharges [7]		Hospital-wide	Observational,Modelling
Daily hospital discharges [42]	Number of COVID-19-discharges per day (countrywide).	Infectious diseases	Modelling

KPI	Definition of KPI*	Medical specialty	Study design
Daily chance of discharge [46]	Unclear, but associated with shorter length of stay. Likely based on in-hospital mortality and length of stay.	Orthopedics	Observational
Number of inpatient discharges [49]	Number of inpatient discharges (until November 2020) at 37 state-owned enterprises (SOE) hospitals.	Not specified	Modelling
Number of outpatient discharges [49]	Number of outpatient discharges (until November 2020) at 37 state-owned enterprises (SOE) hospitals.	Not specified	Modelling
Number of discharged COVID-19 patients [67]	Number of discharged alive COVID patients	Hospital-wide	Modelling
Number of discharged patients with POC [67]	POC: package of care	Hospital-wide	Modelling
Number of discharged patients with no POC [67]	POC: package of care	Hospital-wide	Modelling
Discharge COVID-19 patient after ED visit [85]		Emergency department (ED)	Modelling
Number of discharged COVID patients [86]	Number of patients discharged from hospital with a diagnosis of COVID-19.	ICU	Modelling, Observational
Number of discharged patients [87]	Number of patients discharged from the hospital	Emergency department (ED)	Observational
**C3. Outpatient care activities**			
Outpatient visits [3]	Number of outpatient appointments	Hospital-wide	Modelling, Observational
Total number of appointments [22]	Number of first and follow-up appointments performed in all areas and specialties of dentistry	Dentistry	Observational
Referrals from primary healthcare [22]		Dentistry	Observational
Number of face to face visits [32]	For outpatients, heart failure service and cardic rehabilitation	Cardiology	Observational
Cardiology referrals [32]		Cardiology	Observational
New referrals [32]	For outpatients, heart failure service and cardic rehabilitation	Cardiology	Observational
Number of referrals [35]		Oncology	Observational
Number of consultations [35]		Oncology	Observational
Number of referred patients [48]	Total number of referrals to specialised palliative care.	Palliative care/end-of-life	Observational
Number of emergency visits [49]	Number of emergency visits (until November 2020) at 37 state-owned enterprises (SOE) hospitals.	Not specified	Modelling
Arrival rate [74]	Number of patients arriving per hour	Emergency department (ED)	Modelling
Number of ED visits [87]	Number of oncological patients' ED visits.	Emergency department (ED)	Observational

KPI	Definition of KPI*	Medical specialty	Study design
Number of presentations [89]	Number of presentations at ED. This number included all patients registered at the triage, including patients who left without being seen.	Emergency department (ED)	Observational
**C4. Surgeries**			
Surgeries [3]	Number of nonurgent surgeries	Hospital-wide	Modelling, Observational
Outpatient surgeries [3]	Number of minor surgeries performed in the outpatient service to prevent nosocomial infection	Hospital-wide	Modelling, Observational
Number of surgeries performed [6]		Orthopedics (surgical), Trauma	Observational
Ambulatory surgery rate [9]	The number of ambulatory procedures divided by the total number of nonurgent elective surgeries.	General surgery	Observational
Number of elective surgeries [9]		General surgery	Observational
Number of surgeries performed in the operating room (OR) [22]		Dentistry	Observational
Number of procedures [32]	PPM implants/generator replacements, DC cardioversion, elective coronary angiography, urgent coronary angiography, primary PCI, EP procedures, TAVI, cardiac surgery	Cardiology	Observational
Cases per day at OR [59]		Cardiovascular surgery	Observational
Elective surgeries performed [65]		Hospital-wide	Modelling, Observational
Number of neck of femur fractures surgeries [71]		Orthopedics, Orthopedics (surgical), Trauma	Observational
Operated patients [73]	Number of operated sarcoma patients	Orthopedics (surgical)	Observational
Number of scheduled operations [77]	The total number of scheduled operations performed in our unit.	Pediatric surgery	Observational
Elective waiting list size [92]	number of people on waiting list	Not specified	Modelling
**C5. Other procedures**			
Number of patients received acute reperfusion therapy [5]	Splitted in 3 categories: intravenous thrombolysis, bridging and endovascular therapy.	Neurology	Observational
Laboratory activity [10]	Number of tests for: D-dimer levels, fibrinogen levels (Clauss), anti- Xa activity and factor VIII activity.	Haematology	Observational
Number of exams performed by pathology department [12]	Grouped into different classes: surgical, biopsy, second opinion, molecular, cytology, and PAP test.	Pathology, Oncology	Observational
Thrombolysis performed [14]		Neurology	Observational
Orthogeriatrician review [20]	Percentage of patients reviewed by the regional hospital orthogeriatric service	Orthopedics (surgical)	Observational

KPI	Definition of KPI*	Medical specialty	Study design
Number of biopsies [22]		Dentistry	Observational
Number of oesophageal physiology investigations [25]		Gastroenterology	Observational
Annual HbA1c checks [27]	Percentage of patients that get 2 or more HbA1c checks during a year	Endocrinology	Observational
Annual eye examination [27]	Percentage of (diabetes) patients that get 2 an eye examination during a year	Endocrinology	Observational
Annual microalbuminuria test [27]	Percentage of (diabetes) patients that get microalbuminuria test during a year	Endocrinology	Observational
Annual feet examination [27]	Percentage of (diabetes) patients that get feet examination during a year	Endocrinology	Observational
Annual lipid test [27]	Percentage of (diabetes) patients that get lipid test during a year	Endocrinology	Observational
Thrombolysis [34]	Number of patients received treatment	Neurology	Observational
Thrombectomy [34]	Number of patients received treatment	Neurology	Observational
Population adjusted number of solid organ transplants [61]	Total number of SOT's in a country in a year per one million population.	Cardiology, Transplantation	Observational
Population adjusted number of heart transplants [61]	Total number of HT's in a country in a year per one million population.	Cardiology, Transplantation	Observational
Within-country change in heart transplants (HT) [61]	%, from 2019 to 2020	Cardiology, Transplantation	Observational
Number of tests performed for the selected virus [63]		Laboratory	Modelling
**C6. Diagnoses**			
Diagnostic activity [32]	Number of investigations and ambulatory ECG monitors	Cardiology	Observational
Main diagnoses of hospitalized patients [44]	Counting the number of hospitalized patients with a certain diagnose.	Cardiology, Internal medicine	Observational
Number of patients with specific diagnosis (primary diagnosis) [45]		Gynaecology/Obstetrics (incl. reproductive care)	Observational
DRG code [45]	Counting occurence of DRG code.	Gynaecology/Obstetrics (incl. reproductive care)	Observational
Newly diagnosed cases [73]	Number of new diagnosed sarcoma	Orthopedics (surgical)	Observational
Number of stroke patients [80]		Emergency department (ED)	Observational
Number of COVID-19 positive patients [87]		Emergency department (ED)	Observational
Number of patients [88]	Number of breast cancer patients registered at EUSOMA database.	Oncology	Observational
**C7. Cancer screening**			

KPI	Definition of KPI*	Medical specialty	Study design
Cancer detection rate in colonoscopy [24]	Number of detections divided by number of scans	Gastroenterology, Cancer screening	Observational
Invitation coverage breast cancer [52]	The number of people who received an invitation, as a proportion of the population to be invited year xxxx.	Cancer screening	Observational
Invitation coverage cervical cancer [52]	The number of people who received an invitation, as a proportion of the population to be invited year xxxx.	Cancer screening	Observational
**C8. Case mix / patient complexity**			
Case mix index [44]		Cardiology, Internal medicine	Observational
Patient complexity level [44]		Cardiology, Internal medicine	Observational
Case mix index [45]		Gynaecology/Obstetrics (incl. reproductive care)	Observational
**D1. Process quality indicators**			
Stroke admission site [2]	Yes/no: either a stroke unit (including intensive care units and neurosurgery departments) ('Yes') or other admission sites (like observation units within the hospital) ('No')	Neurology, Geriatry	Observational
Reperfusion treatment [2]	Yes: receiving thrombolysis, thrombectomy, or both. No otherwise.	Neurology, Geriatry	Observational
Swallowing assessment [2]	Either "yes," indicating that the patient underwent an assessment or that no assessment was necessary, or "no" due to limited availability of speech therapists.	Neurology, Geriatry	Observational
Assessment for rehabilitation [2]	Yes: the initial evaluation conducted by an occupational therapist, a physiotherapist, or both. No otherwise.	Neurology, Geriatry	Observational
Early supported discharge (ESD) [2]	Yes/no, no further definition given.	Neurology, Geriatry	Observational
Rate of exams with significant lesions [19]	The following were considered significant lesions: P2 and P1 lesions according to the Saurin classification for intestinal bleeding; ulceration; erosions or strictures in the context of suspected/established Crohn's disease; small-bowel tumors and smallbowel polyps.	Gastroenterology	Observational
Rate of adequately prepared exams [19]	An exam was considered adequately prepared if both small-bowel and colon were adequately visualized. For small-bowel visualization, the MiroCam definition was used; for colonic mucosa visualization, a subjective definition was used by the CE viewer (adequate or inadequate visualization).	Gastroenterology	Observational

KPI	Definition of KPI*	Medical specialty	Study design
Rate of complete exams [19]	An exam was considered complete if the capsule reached the cecum/colon or stoma bag (in patients who have had ileocolonic resection or other relevant surgery) during recording time.	Gastroenterology	Observational
Completion of procedure [29]	Percentage of completed endoscopic ultrasounds, of all performed endoscopic ultrasounds	Gastroenterology	Observational
Diagnostic accuracy for endoscopic ultrasound fine needle aspiration [29]	Percentage of accurate diagnoses in all diagnoses after procedure	Gastroenterology	Observational
Perceived quality of care [30]	Good/poor/neutral, as perceived by nurses	ICU	Observational
Perceived quality of care [31]	Likert scale, 1 to 5	Hospital-wide	Observational
Professional autonomy [31]	Likert scale, 1 to 5	Hospital-wide	Observational
Adequate time for patient care [31]	Likert scale, 1 to 5	Hospital-wide	Observational
X-ray of surgical specimen in cases treated with conservative surgery with microcalcification only [58]	Desirable outcome. Yes/no.	Oncology	Observational
MRI before surgery for invasive cases [58]	Desirable outcome. Yes/no.	Oncology	Observational
Only one surgical operation for invasive cancer treatment [58]	Desirable outcome. Yes/no.	Oncology	Observational
At least 10 lymph nodes removed for axillary dissection (sampling excluded) [58]	Desirable outcome. Yes/no.	Oncology	Observational
Only one surgical operation for non-invasive cancer treatment [58]	Desirable outcome. Yes/no.	Oncology	Observational
No axillary dissection for non-invasive cases [58]	Desirable outcome. Yes/no.	Oncology	Observational
Only sentinel lymph nodes examination in pN0 cases [58]	Desirable outcome. Yes/no.	Oncology	Observational
Maximum 3 lymph nodes removed as sentinel lymph nodes [58]	Desirable outcome. Yes/no.	Oncology	Observational
Conservative surgery for non-invasive cases up to 2 cm [58]	Desirable outcome. Yes/no.	Oncology	Observational

KPI	Definition of KPI*	Medical specialty	Study design
Conservative surgery for invasive cases up to 3 cm (non-invasive component included) [58]	Desirable outcome. Yes/no.	Oncology	Observational
Radiotherapy after conservative surgical treatment [58]	Desirable outcome. Yes/no.	Oncology	Observational
Post-mastectomy radiotherapy for pN2a cases [58]	Desirable outcome. Yes/no.	Oncology	Observational
Endocrine therapy indication for endocrine-sensitive invasive cases [58]	Desirable outcome. Yes/no.	Oncology	Observational
Chemotherapy indication for invasive, hormone receptor negative cases if pT > 1 cm or pN +. [58]	Desirable outcome. Yes/no.	Oncology	Observational
Chemotherapy and trastuzumab indication for invasive HER2 + cases [58]	Desirable outcome. Yes/no.	Oncology	Observational
Primary chemotherapy indication for inflammatory cancer [58]	Desirable outcome. Yes/no.	Oncology	Observational
Cross clamp times [59]		Cardiovascular surgery	Observational
ECG before arrival at the hospital [79]	Number of patients that got an ECG before arrival at the hospital.	Cardiology	Observational
Emergency department bypass [79]	Number of patients that got direct transport to the catheterization laboratory bypassing the emergency department.	Cardiology	Observational

D2. Quality scores

KPI	Definition of KPI*	Medical specialty	Study design
Quality of maternity and newborn care index (score) [1]	Score ranging from 0 to 400 based on questionnaire answers regarding 40 questions in 4 categories (100 points per category):<br>Cat 1: Provision of care-score 1. No pain relief 2. Mode of birth (vaginal, planned cesarean, emergency cesarean) 3. Episiotomy/funal pressure/no pain relief after cesarean 4. No skin to skin 5. No early breastfeeding 6. Inadequate breastfeeding support 7. No rooming-in 8. Not allowed to stay with the baby as wished 9. No exclusive breastfeeding at discharge 10. No immediate attention when needed<br>Cat 2: Experience of care-score 1a. No freedom of movements during labor 1b. No consent requested for vaginal examination in prelabor cesarean 2a. No choice of birth position (in SVB) 2b. No consent requested (for IVB) 2c. No information on newborn after cesarean 3. No clear/effective communication from 4. No involvement in choices 5. Companionship not allowed 6. Not treated with dignity 7. No emotional support 8. No privacy 9. Abuse (physical/verbal/emotional 10. Informal payment<br>Cat 3: Availability of physical and human resources-score 1. No timely care by HCP at facility arrival 2. No information on maternal danger signs 3. No information on newborn danger signs 4. Inadequate room comfort and equipment 5. Inadequate number of women per rooms 6. Inadequate room cleaning 7. Inadequate bathroom 8. Inadequate partner visiting hours 9. Inadequate HCP number 10. Inadequate HCP professionalism<br>Cat 4: Reorganizational changes due to COVID- 19-score 1 Difficulties in attending routine antenatal visits 2 Any barriers in accessing the facility 3 Inadequate infographics 4 Inadequate wards reorganization 5 Inadequate room reorganization 6 Lacking one functioning accessible hand-washing station 7 HCP not always using PPE 8 Insufficient HCP number 9 Communication inadequate to contain COVID-19-related stress 10 Reduction in QMNC (quality of maternal and newborn care) due to COVID-19<br>All measured in %, i.e., the number of respondents that answered "Yes" divided by the total number of responses.	Gynaecology/Obstetrics (incl. reproductive care)	Observational

KPI	Definition of KPI*	Medical specialty	Study design
Quality of acute myocardial infarction care [4]	Used the 2020 European Society of Cardiology (ESC) Association for Acute Cardiovascular Care (ACVC) QIs for AMI, which comprise 26 indicators. 16 could be assessed for this study, all expressed in percentages: hospital use of hs- cTn for NSTEMI; pre- hospital interpretation of ECG for STEMI; reperfusion among eligible for STEMI; timely reperfusion for STEMI; invasive coronary angiography within 24 hours for NSTEMI; radial access for invasive procedures; median time (min) from ECG to arterial access for STEMI, median (IQR) LVEF assessment before hospital discharge; in- hospital measurement of LDL- C+; adequate P2Y12 inhibition on discharge; parenteral anticoagulation; dual antiplatelet therapy on discharge; high- intensity statin on discharge; ACEi for patients with reduced LVEF; beta blockers for patients with reduced LVEF; composite all- or-none.	Cardiology	Observational
Opportunity-based stroke care quality score [13]	Calculated as the number of indicators fulfilled for a patient divided by the number of indicators estimated to be relevant for that patient. In the case of missing single indicators, the opportunity based score was calculated as all relevant available and fulfilled indicators divided by all relevant available indicators. The indicators: Acute treatment (admission to stroke unit <24 h from onset, scan < 6 h from admission, revascularization offered, timing of thrombolysis, and groin puncture in case of thrombectomy), prophylactic treatment, and the timing thereof (start of antiplatelet within the second day of admission, start of oral anticoagulant < 14 days for patients eligible), carotid vessel imaging, and early rehabilitation (physio- and occupational therapy evaluation, out-of-bed orders, nutrition, and swallow screening).	Stroke specific	Observational

KPI	Definition of KPI*	Medical specialty	Study design
SAB care quality point score [15]	The total quality of care for every patient was assessed by a score system: the presence of each quality indicator was included with one score point into the score system; hence, the minimum-total score was 0 and the maximum total score was 6 points. The quality indicators: correct choice of antibiotic; adequate dosage of antibiotic; sufficient duration of therapy; early start of therapy after receipt of findings; adequate focus search; taking control blood cultures 3–4 days after starting adequate antibiotic therapy.	Infectious diseases	Observational
Composite indicator for safety and care appropriateness [16]	Linear programming model that takes inputs (measured a certain point in time) and variables (weights) to maximize the sum of the products of those, which is then defined as the composite indicator (CI). Input factors for efficiency and productivity are: bedsore rate; rate of in-hospital-developed septicemia (postoperative); rate of catheter-related bloodstream infection events; rate of postoperative pulmonary embolism events and thromboembolisms; rate of performed minor surgeries out of potential minor surgeries; rate of readmissions within 30 days after discharging; rate of inpatients staying hospitalized for more than 30 days.	Hospital-wide	Modelling
Total Factor Productivity (TFP) for safety and care appropriateness [16]	Calculated factor that relates the outcome of two CI measuring moments. It takes the optimal variables and the inputs of both CI linear programming models as input. Score > 1 indicates the hospital improved.	Hospital-wide	Modelling
Treatment quality endoscopic retrograde cholangiopancreatography [29]	Measured using 4 KPIs, measured as percentage (number of patients the KPI applies to divided by total number of patients undergoing the treatment), measuring how often doctors succeed in different parts of the treatment: 1. Overall CBD cannulation; 2. CBD cannulation in naïve papilla; 3. Stone clearance rate at first ERCP; 4. Biliary stricture decompression. The guidelines include recommended percentages for each KPI, for example 2 must be $> 85\%$.	Gastroenterology	Observational
Consultation quality score [43]	The sum of 'yes' responses to the survey questions. This was a score out of 9 for patients and out of 10 for clinicians.	Neurosurgery, Endocrinology	Observational

KPI	Definition of KPI*	Medical specialty	Study design
Aggregated hospital performance measure [65]	Elective surgeries performed $*$ %lifesaving surgeries + COVID-19 admissions - critical care rejections - deaths	Hospital-wide	Modelling, Observational
Stroke care performance [66]	Patients were identified from the Danish Stroke Registry (DSR) and this data included stroke care performance measures relating to the quality of diagnosis, workup, treatment, care and early rehabilitation. Stroke care performance measures: opportunity-based score; all or no score; reperfusion therapy with EVT+IVT; reperfusion therapy with EVT; reperfusion therapy with IVT; door-to-needle $<$ 45 min (IVT); door-to-groin puncture $<$ 180min (EVT); admission to stroke unit on day of hospitalisation; platelet inhibitors (AIS without AFIB) $\leq$ 2days after hospitalisation; anticoagulation $\leq$ 14 days after hospitalisation (AIS/TIA with AFIB); CT/MRI $<$ 6 hours from admission; physiotherapy assessment $\leq$2days after hospitalisation; occupational therapy assessment $\leq$ 2 days after hospitalisation; out-of-bed orders on day of admission; nutritional screening $\leq$ 2 days after hospitalisation; swallowing screen (indirect) on day of admission; swallowing screen (direct) on day of admission; carotid vessel imaging $\leq$ 2 days after hospitalisation	Stroke specific	Observational
Quality of care (score) [66]	Quality of care is a opportunity-based score. It was obtained from the DSR using the registered stroke care performance measures covering acute and prophylactic treatment, carotid vessel imaging and early rehabilitation. For each patient, they calculated an opportunity-based score defined as the number of fulfilled performance measures divided by the number of relevant performance measures as well as an all or no score, with a score of 1 if all relevant performance measures were met and a score of 0 if not all relevant measures were met.	Stroke specific	Observational

KPI	Definition of KPI*	Medical specialty	Study design
Quality of hip fracture treatment [72]	Quality of in-hospital care is measured by the following quality indicators: preoperative optimization reached within 4 hours of admission for hip fracture; surgery delay (less than 24 and 36 hours); mobilization reached within 24 hours of surgery; assessment of nutrition status after surgery; assessment of the basic mobility scores preoperatively and at discharge; rehabilitation program sent to the community; and osteoporosis and fall prophylaxis after surgery.	Orthopedics	Observational
Surgical management indicators [73]	Indicators for surgical management of sarcoma: M0 cases newly diagnosed via surgery; Surgery center: Outside the labeled center / In the labeled center / Referred by the labeled center to a specialized service center; Surgery after biopsy: yes/no (%); MDB assessing the planned resection quality: missing/yes/no (%); neoadjuvant treatment: missing data/yes/no (%); quality of the first surgery: missing or unknown (%) / R0 (%) / R1 (%) / R2 (%); re-excision: missing/yes/no (%).	Orthopedics (surgical)	Observational
Stroke care performance indicators (acute stroke) [80]	Acute management indicators: SARS-CoV-2 positive at admission (%); delayed stroke unit admission (%); reperfusion therapy (%); intravenous thrombolysis in-house; intravenous thrombolysis (outside); endovascular therapy (%); revascularization therapy (%); carotid endarterectomy; carotid stenting. Process times indicators: onset-to-admission (min), median (IQR); door-to-imaging (min) (IVT), median (IQR); door-to-needle (min), median (IQR); onset-to-groin (min), median (IQR); door-to-groin (min), median (IQR). Case management indicators: admission modality (%); discharge modality (%); length of hospitalization (rehabilitation), days, median (IQR); length of hospitalization (excl. rehab), days, median (IQR); medication at discharge (%).	Emergency department (ED), Stroke specific	Observational

KPI	Definition of KPI*	Medical specialty	Study design
ICSI key performance indicators [94]	ICSI = intracytoplasmic sperm injection (ICSI) treatment to infertile couples. KPIs: ICSI Implantation rate 25 case average (combined cleavage and blastocyst embryos); ICSI damage rate (definition: number of degenerated eggs/number of metaphase II (MII) mature eggs injected); ICSI normal fertilisation rate (definition: number of degenerated eggs/number of metaphase II (MII) mature eggs injected); proportion of MII oocytes at ICSI (definition: number of MII eggs at ICSI/number of eggs collected for ICSI); average number of eggs collected for ICSI cases (definition: average number of eggs collected for ICSI); average maternal age ICSI cases (definition: average maternal age of ICSI patients).	Gynaecology/Obstetrics (incl. reproductive care)	Observational
**D3. Missed care**			
Same-day surgery cancellation rate [9]		General surgery	Observational
Missed appointments [22]		Dentistry	Observational
Non-attendance rate [25]		Gastroenterology	Observational

KPI	Definition of KPI*	Medical specialty	Study design
Missed nurse care [30]	Any aspect of required nursing care that is omitted (in part or in whole) or delayed. Aspects, could be indicated as 'missed' by nurse: Ambulation 3 times per day or as ordered. Turning patient every 2 h. Feeding patient when the food is still warm. Setting up meals for patient who feeds themselve. Medications administered within 30 min before or after scheduled time. Vital signs assessed as ordered. Monitoring intake/output. Full documentation of all necessary data. Patient teaching about procedures, tests and other diagnostic studies. Emotional support to patient and/or family. Patient bathing/skin care. Mouth care. Nursing staffs' hand washing. Patient discharge planning and teaching. Bedside glucose monitoring as ordered. Patient assessments performed each shift. Focused reassessments according to patient condition. IV/central line site care and assessments according to hospital policy. Response to call light is initiated within 5 min. PRN medication requests acted on within 15 min. Assess effectiveness of medications. Attend interdisciplinary care conference whenever held. Assist with toileting needs within 5 min of request.	ICU	Observational
Health losses due to avoidable cancer deaths caused by diagnostic delay [37]	Calculated using different models, taking the excess deaths as input.	Oncology	Modelling
Critical care rejections [65]	The number of patients not being able to capture the appropriate bed or staff required for their level of care on CC within a given time-frame.	Hospital-wide	Modelling, Observational
Suspension rate [77]	Suspension rate, which indicates the number of patients who were not admitted and were not operated for some reason.	Pediatric surgery	Observational
Left without being seen [89]	Number of patients that left the ED without being seen.	Emergency department (ED)	Observational
Missed referrals during the pandemic [92]	Estimate of number of referrals that did not take place.	Not specified	Modelling
Proportion of missed referrals returning [92]	Proportion of patients that return (present themselves) within 12 months after their 'actual' (compared to non-pandemic times) referral date.	Not specified	Modelling
**D4. Laboratory performance**			
Turnaround time [10]		Haematology	Observational

KPI	Definition of KPI*	Medical specialty	Study design
Quality of tests [10]	Expected quality from laboratory employees' perspective.	Haematology	Observational
Turaround time [12]	Turn-Around-Time (TAT) metrics released by the Lombardy Region for the pathology laboratories accredited with the national health system: this procedure establishes that at least 90% of the diagnoses must be carried out within a timeframe pre-specified for each category of exams. Turnaround time here is measured as the percentage of exams that meets the prespecified deadline.	Pathology, Oncology	Observational
Number of positive tests [62]		Laboratory	Modelling
Average number of tests per patient [62]		Laboratory	Modelling
Average test duration and median duration [62]		Laboratory	Modelling
Positivity rate [62]		Laboratory	Modelling
Percentage of patient having to take two or more tests [62]		Laboratory	Modelling
Proportion of tests marked as 'NOT VALID' [62]		Laboratory	Modelling
**D5. Safety and adverse events**			
Inpatient falls rate [8]	Percentage of patients that experienced any postoperatively fall during their hospital stay	Orthopedics (surgical), Trauma	Observational
Overnight-stay rate [9]	Number of patients that spent the first night after surgery at the ambulatory surgery unit but still were discharged before completing 24h after admission	General surgery	Observational
Perceived patient safety [30]	Good/poor/neutral, as perceived by nurses	ICU	Observational
Full time equivalent dedicated infection control nurses per 100 beds [57]		Infectious diseases	Observational
Alcohol-based hand rub usage [57]		Infectious diseases	Observational

KPI	Definition of KPI*	Medical specialty	Study design
Number of inpatients without adverse events [69]	This variable refers to the total number of patients seen that did not suffer any preventable adverse event during treatment (especially during their stay in hospital), including but not limited to septicemia, bloodstream infections, thromboembolisms, bedsores, and death. Although we could not assess whether the patients' quality of life has improved, this variable can capture whether the care process was well conducted, i.e., care was safe and appropriate, following evidence-based guidelines.	Hospital-wide	Modelling
Hospitalization rate (HR) [77]	Indicates the proportion of patients who required hospitalization because they could not be discharged after undergoing surgery under the MOS system.	Pediatric surgery	Observational
**D6. Patient satisfaction**			
Patient satisfaction [19]	Question 1 (Q1): Do you consider the steps of this medical procedure simple? Possible answers: yes or no. Question 2 (Q2): Concerning the place where the exam was held, what is your degree of satisfaction? Possible answers: very satisfied (VS), moderately satisfied (MS), unsatisfied (US), or very unsatisfied (VU). In order to simplify statistical analysis, the authors considered that the patient was satisfied if the answer for Q1 was "Yes" and the answer for Q2 was either "VS" or "MS." For different combinations of answers, the patient was considered not satisfied.	Gastroenterology	Observational
Consultation satisfaction [43]	Patient's satisfaction about consultation.	Neurosurgery, Endocrinology	Observational
Patient satisfaction [77]	Degree of family satisfaction (survey carried out among parents or legal guardians of the patients who underwent MOS).	Pediatric surgery	Observational
**E1. Access time (outside hospital)**			
First appointment time [3]	Number of first outpatient vists occurred outside the legal time for each medical situation	Hospital-wide	Modelling, Observational
Surgery waiting time [3]	Average number of days before having surgery	Hospital-wide	Modelling, Observational
Onset-to-door time (ODT) [14]		Neurology	Observational

KPI	Definition of KPI*	Medical specialty	Study design
Composite indicator for access [16]	Linear programming model that takes inputs (measured a certain point in time) and variables (weights) to maximize the sum of the products of those, which is then defined as the composite indicator (CI). Input factors for efficiency and productivity are: rate of first medical appointments within the legally fixed period; rate of enrolled patients on the waiting list for surgery within the legally pre- scribed time; average time before surgery.	Hospital-wide	Modelling
Total Factor Productivity (TFP) for access [16]	Calculated factor that relates the outcome of two CI measuring moments. It takes the optimal variables and the inputs of both CI linear programming models as input. Score >1 indicates the hospital improved.	Hospital-wide	Modelling
Time to ward admission $<$ 4h [20]	Percentage of patients that is admitted within 4 hours	Orthopedics (surgical)	Observational
Time to test [25]	Number of days between the date on which the referral was received and the date on which the patient attended their appointment. For all referrals received between March 18 2020 and September 29 2020, when service was halted, this was calculated as the days between the date on which service resumed in the department (September 29 2020) and the date on which the patient attended their appointment.	Gastroenterology	Observational
Onset to door time [34]	The time from stroke onset, or from the last moment the patient was known without symptoms, to emergency department (ED) arrival.	Neurology	Observational
Average wait time from referral to first consultation [35]		Oncology	Observational
Average wait time from referral to diagnosis [35]		Oncology	Observational
Average wait time from referral to acquistion of CT scan [35]		Oncology	Observational
Average wait time from referral to surgery [35]		Oncology	Observational
Waiting time [41]	Time in days from referral to first appointment	Orthopedics	Modelling, Observational
Transfer time [50]	Time from acceptance to admission.	Trauma	Observational
Percentage of screened people (breast cancer) [52]	The percentage of people that were screened within 40 days after their invitation.	Cancer screening	Observational

KPI	Definition of KPI*	Medical specialty	Study design
Screening interval (breast cancer) [52]	Was calculated for each person screened in 2019 and 2020 as the number of months between the current screening date, and his or her previous screening date. Mean screening intervals per week, with 95% CI were calculated.	Cancer screening	Observational
Screening interval (cervical cancer) [52]	Was calculated for each person screened in 2019 and 2020 as the number of months between the current screening date, and his or her previous screening date. Mean screening intervals per week, with 95% CI were calculated.	Cancer screening	Observational
Time from referral to TAVR clinic [53]	Time taken from referral to TAVR clinic.	Cardiovascular surgery	Observational
Surgery within 42 days from the first diagnostic exam [58]	Desirable outcome. Yes/no.	Oncology	Observational
Surgery within 30 days from indication to treatment [58]	Desirable outcome. Yes/no.	Oncology	Observational
Surgery within 60 days from screening mammography [58]	Desirable outcome. Yes/no.	Oncology	Observational
Radiotherapy within 12 weeks from surgical intervention (if adjuvant chemotherapy not indicated) [58]	Desirable outcome. Yes/no.	Oncology	Observational
Number of first medical appointments within the legally defined time [69]	Portuguese State has defined a maximum legal time such that hospitals must schedule the first medical appointment once the reference letter has been received. The closer the number of first medical appointments within the legally defined time is to the effective number of first medical appointments, the timelier the hospital is.	Hospital-wide	Modelling
Number of hip surgeries in the first 48h after fracture [69]	This variable measures the number of patients over 65 years old with hip surgery in the first 48 h after fracture.	Hospital-wide	Modelling
Number of registers on the surgery waiting list within the legally defined time [69]	As in the case of first medical appointments, there is a maximum legal time for registering and scheduling surgeries (either minor or major).	Hospital-wide	Modelling
Number of delayed first medical appointments [69]	This variable results from subtracting the number of timely first appointments from the total first appointments.	Hospital-wide	Modelling
Number of registers on surgery waiting list beyond the legally defined time [69]		Hospital-wide	Modelling

KPI	Definition of KPI*	Medical specialty	Study design
Number of hip surgeries beyond 48h after fracture [69]	Two days is the recommended time limit for patient presentation for hip surgery, especially when the patient is 65 years old or older.	Hospital-wide	Modelling
Time interval between diagnosis and surgery [73]	Diagnosis: date of first diagnostic procedure, such as biopsy or surgery; surgery: date of first curative surgery.	Orthopedics (surgical)	Observational
Waiting time TWW: 2-week wait standard [76]	Percentage of patients who see the cancer specialist within 2 weeks of an urgent referral by GP or screening programme referral for suspected cancer	Oncology	Observational
Waiting time DTT: 31-day decision to treatment standard [76]	Percentage of patients who have the definitive treatment of cancer within 31 days of having a decision to treat. Patients should have their first definitive treatment of cancer within 31 days of having a decision to treat: patients should have their subsequent drug treatment for cancer within 31 days; patients should have their subsequent surgery for cancer within 31 days; patients should have their subsequent radiotherapy for cancer within 31 days.	Oncology	Observational
Waiting time RTT: 62-day referral to treatment standard [76]	Percentage of patients who have their first definitive treatment within 62 days of referral.	Oncology	Observational
Symptom-to-contact time [79]	Measured in minutes.	Cardiology	Observational
Elective waiting time [92]	Referral-to-treatment time	Not specified	Modelling
Number of people waiting more than 52 weeks [92]		Not specified	Modelling
**E2. Sojourn time (inside hospital)**			
Hip surgeries [3]	Number of hip fracture surgeries performed under 48 hours after arrival	Hospital-wide	Modelling, Observational
Admission-to-treatment time [5]	Time of hospital arrival to time of treatment initiation. Treatment initiation was defined as the time of administration of intravenous alteplase in cases of IVT alone and bridging therapy or in case of EVT alone the time of groin puncture.	Neurology	Observational
Admission to-image time [5]	Time from hospital arrival to first image	Neurology	Observational
Image-to-treatment time [5]	Time from first image to treatment initiation	Neurology	Observational
First case delay [6]	Delay from the scheduled time for skin incision of the first patient of the day	Orthopedics (surgical), Trauma	Observational
Time to surgery [8]	Time from ED registration until arrival in operation theatre	Orthopedics (surgical), Trauma	Observational

KPI	Definition of KPI*	Medical specialty	Study design
Time in the operating theatre [8]	Time from the arrival of the patient in the operating room theatre holding bay until leaving the recovery room.	Orthopedics (surgical), Trauma	Observational
Door-to-CT time [14]		Neurology	Observational
Door-to-needle time (DNT) [14]		Neurology	Observational
Door-to-groin time (DGT) [14]		Neurology	Observational
Time to surgery $<$ 48 [20]	Percentage of patients that has surgery within 48 hours	Orthopedics (surgical)	Observational
Door to groin time [34]		Neurology	Observational
Door to needle time [34]		Neurology	Observational
Door to CT time [34]		Neurology	Observational
Number of reneging patients [36]	Number of COVID-patients that go to another hospital because the waiting time is too long.	Infectious diseases,ICU	Modelling
Time from admission to surgery [38]	Measured in hours	General surgery	Observational
Time from admission to time of surgery [50]	The mean time from admission to surgery.	Trauma	Observational
Time from TAVR clinic to TAVR [53]	Time from TAVR clinic to procedure.	Cardiovascular surgery	Observational
Procedure time [53]	Distribution of procedure length (in minutes)	Cardiovascular surgery	Observational
Bypass times [59]		Cardiovascular surgery	Observational
Induction times [59]		Cardiovascular surgery	Observational
Total intraoperative time [59]		Cardiovascular surgery	Observational
Days before surgery [69]	Total number of days all admitted patients stay in the hospital wards waiting for major scheduled surgery.	Hospital-wide	Modelling
Time to surgery for neck of femur fractures surgeries [71]	Number of hours between admission (via ED) and start of surgery.	Orthopedics, Orthopedics (surgical), Trauma	Observational
Time to be admitted to orthopaedic ward [71]	Number of hours between admission (via ED) and admission to the orthopaedic ward.	Orthopedics, Orthopedics (surgical), Trauma	Observational
Time until orthogeriatric review [71]	Number of hours between surgery completion and orthogeriatric review.	Orthopedics, Orthopedics (surgical), Trauma	Observational
Service time [74]	Time during the medical examination	Emergency department (ED)	Modelling
Patient experience times [78]	The length of time in hours patients wait in ED before they are either discharged or admitted to hospital.	Emergency department (ED)	Observational
Duration of emergency medical services at scene [79]	Measured in minutes.	Cardiology	Observational

KPI	Definition of KPI*	Medical specialty	Study design
Transport time by emergency medical service vehicle [79]	Measured in minutes.	Cardiology	Observational
Contact-to-door time [79]	Measured in minutes.	Cardiology	Observational
Door-to-cath time [79]	Measured in minutes.	Cardiology	Observational
Cath-to-puncture time [79]	Measured in minutes.	Cardiology	Observational
Puncture-to-balloon time [79]	Measured in minutes.	Cardiology	Observational
Door-to-balloon time [79]	Measured in minutes.	Cardiology	Observational
Contact-to-balloon time [79]	Measured in minutes.	Cardiology	Observational
Time to treatment [82]	The time spent on all the activities prior to the treatment, i.e., time from the patient entered the ED to start of the treatment. Measured in hours.	Emergency department (ED)	Modelling
Waiting time [82]	Amount of time patients wait in the ED.	Emergency department (ED)	Modelling
Door-to-physician times [89]	Proportion of patients visited within 60 min after the triage. Tuscany health system currently uses this measurement as benchmarking standard.	Emergency department (ED)	Observational
Time from diagnosis to theater [90]	Time from hospital presentation to theater, in hours.	Orthopedics (surgical)	Observational
Call for patient to anesthetic room [90]	In minutes.	Orthopedics (surgical)	Observational
Surgeon operating time [90]	In minutes.	Orthopedics (surgical)	Observational
Total time in theater (TTT) [90]	In minutes.	Orthopedics (surgical)	Observational
Time from ward to recovery [90]	In minutes.	Orthopedics (surgical)	Observational
**E3. Length of stay**			
Length of stay [2]	Number of days	Neurology, Geriatry	Observational
Inpatient duration [3]	Number of inpatients staying less than 30 days in the inpatient service	Hospital-wide	Modelling, Observational
Number of inpatient hospitalization days [7]		Hospital-wide	Observational,Modelling
Length of stay [8]		Orthopedics (surgical), Trauma	Observational
Length of stay in hospital [14]		Neurology	Observational
Length of stay in emergency room [14]		Neurology	Observational
Length of stay in hospital [33]	Measured in 3 categories: less than 7 days, 7-14 days, more than 14 days.	Hospital-wide	Observational

KPI	Definition of KPI*	Medical specialty	Study design
Length of stay in ED in minutes [34]		Neurology	Observational
Length of stay in hospital, in days [34]		Neurology	Observational
Length of stay [44]	Number of days patient is in hospital.	Cardiology, Internal medicine	Observational
Length of stay [46]	Number of days	Orthopedics	Observational
Length of stay [50]	Mean length of stay at hospital (days).	Trauma	Observational
Length of hospital stay [51]	Length of hospitalization.	Not specified	Modelling
Length of admission [53]		Cardiovascular surgery	Observational
Length of stay (LoS) in hospital ward (HW) until admission in ICU [60]		Infectious diseases	Modelling
LoS in HW until discharge from HW [60]		Infectious diseases	Modelling
LoS in ICU until discharge from ICU [60]		Infectious diseases	Modelling
LoS in HW until death in HW [60]		Infectious diseases	Modelling
LoS in ICU until death in ICU [60]		Infectious diseases	Modelling
Length of ICU stay [59]		Cardiovascular surgery	Observational
ICU admission duration [67]	The number of days spent by admitted patients in the ICU.	Hospital-wide	Modelling
Hospital days [69]	Total number of days spent by all inpatients in the hospital wards.	Hospital-wide	Modelling
Length of stay at orthopaedic ward after surgery [71]	Number of days to discharge from orthopaedic ward after surgery.	Orthopedics, Orthopedics (surgical), Trauma	Observational
Length of stay [82]	Time patients spend in the ED, i.e. time between entering and leaving ED. Measured in hours.	Emergency department (ED)	Modelling
Length of stay [84]	Length of stay at hospital of mastectomy patients	General surgery	Observational
Length of stay (LOS) [86]	Hospital length of stay prior to ICU admission.	ICU	Modelling, Observational
Duration of stay [87]	The number of days spent in the ED by oncology patients.	Emergency department (ED)	Observational
Door-to-final disposition (length-of-stay) times [89]	The proportion of patients with a length-of-stay under 4 hours. Tuscany health system currently uses this measurement as benchmarking standard.	Emergency department (ED)	Observational
Length of stay [90]	Time from presentation to hospital discharge (in days).	Orthopedics (surgical)	Observational
Length of hospital stay [91]		Not specified	Modelling

KPI	Definition of KPI*	Medical specialty	Study design
Time in normal ward before ICU [91]		Not specified	Modelling
Time in normal ward after ICU [91]		Not specified	Modelling
Time in ICU [91]		Not specified	Modelling
**F1. Alternative care delivery**			
Number of telephone clinics [32]	For outpatients, heart failure service and cardic rehabilitation	Cardiology	Observational
Patient portal waiting time [54]	The waiting time from sending a request until receiving a reply.	Not specified	Observational
Portal waiting time target rate [54]	Number of patients that got a response via the patient portal earlier than their maximum accepted waiting time.	Not specified	Observational
Maximum acceptable portal waiting time [54]	A patient's maximum acceptable waiting time from sending a request until receiving a reply (according to the patient).	Not specified	Observational
Substitution rate (SR) [77]	The proportion of potentially outpatient procedures performed under the MOS system with respect to the total number of such procedures schedule.	Pediatric surgery	Observational
Ambulatory rate [77]	Measures the proportion of surgical procedures performed on an outpatient basis in relation to the total number of surgical procedures performed.	Orthopedics	Observational
**F2. Other**			
Operational costs [3]	Monthly operational expenses, excluding capital and staff	Hospital-wide	Modelling, Observational
Infection prevention costs [11]	Costs for PPE, PCR tests and personnel	Hospital-wide	Modelling
Number of home visits [32]	For outpatients, heart failure service and cardic rehabilitation	Cardiology	Observational
Urgency-weighted number of patients that receive care at home from nurses that work at a hospital [39]	Per day	Hospital-wide	Modelling
Total health care expenditure [62]	US dollars per capita	Infectious diseases	Observational

References

[1] A. Abderhalden-Zellweger, S. Grylka-Baeschlin, A. N. Mueller, E. P. Valente, A. Cerimagic, D. Drandic, M. Kurbanovic, R. Virginie, E. de La Rochebrochard, K. Lofgren, C. Miani, S. Batram-Zantvoort, L. Wandschneider, M. Lazzerini, B. Covi, I. Mariani, S. Morano, I. Chertok, E. Hefer, R. Artzi-Medvedik, E. Pumpure, D. Rezeberga, G. Jansone-Santare, D. Jakovicka, A. R. Knoka, K. P. Vilcane, A. Liepinaitiene, A. Kondrakova, M. Mizgaitiene, S. Juciute, M. Arendt, B. Tasch, I. H. Nedberg, S. Kongslien, E. S. Vik, B. Baranowska, U. Tataj-Puzyna, M. Wegrzynowska, R. Costa, C. Barata, T. Santos, C. Rodrigues, H. Dias, M. R. Otelea, J. Radetic, J. Ruzicic, Z. Drglin, B. M. Ponikvar, A. Bohinec, S. Brigidi, L. M. Castaneda, H. Elden, V. Sengpiel, K. Linden, M. Zaigham, C. De Labrusse, A. Abderhalden, A. Pfund, H. Thorn, S. Grylka, M. Gemperle, and A. Mueller. Quality of ma-

ternal and newborn care in Switzerland during the COVID-19 pandemic: A cross-sectional study based on WHO quality standards. *International Journal of Gynecology and Obstetrics*, 159(S1):70–84, 2022. Place: Ireland.

[2] T. Abzhandadze, M. T. Hoang, M. Mo, S. Mostafaei, P. G. Jurado, H. Xu, K. Johnell, M. Von Euler, M. Eriksdotter, and S. Garcia-Ptacek. COVID-19 Pandemic and Stroke Care in Patients with Dementia Compared to Other Stroke Patients. *Journal of the American Medical Directors Association*, 25(7):105011, 2024. Place: United States.

[3] G. P. Afonso, J. R. Figueira, and D. C. Ferreira. Dealing with uncertainty in healthcare performance assessment: a fuzzy network-DEA approach with undesirable outputs. *INTERNATIONAL TRANSACTIONS IN OPERATIONAL RESEARCH*, 2024.

[4] Suleman Aktaa, Mohammad E. Yadegarfar, Wu Jianhua, Muhammad Rashid, Mark de Belder, John Deanfield, Francois Schiele, Mark Minchin, Mamas Mamas, and Chris P. Gale. Quality of acute myocardial infarction care in England and Wales during the COVID-19 pandemic: linked nationwide cohort study. *BMJ Quality & Safety*, 31(2):116–122, 2022.

[5] V. L. Altersberger, L. J. Stolze, M. R. Heldner, H. Henon, N. Martinez-Majander, C. Hametner, A. Nordanstig, A. Zini, S. Nannoni, B. Gonçalves, C. H. Nolte, P. Baumgartner, A. Kastrup, P. Papanagiotou, G. Kägi, R. R. Leker, M. Zedde, A. Padovani, A. Pezzini, V. Padjen, C. W. Cereda, G. Ntaios, L. H. Bonati, L. A. Rinkel, U. Fischer, J. F. Scheitz, S. Wegener, G. Turc, P. Michel, M. Gentile, A. Rentzos, P. A. Ringleb, S. Curtze, C. Cordonnier, M. Arnold, P. J. Nederkoorn, S. T. Engelter, and H. Gensicke. Maintenance of Acute Stroke Care Service during the COVID-19 Pandemic Lockdown. *Stroke*, 52(5), 2021.

[6] M. Andreata, M. Faraldi, E. Bucci, G. Lombardi, and L. Zagra. Operating room efficiency and timing during coronavirus disease 2019 outbreak in a referral orthopaedic hospital in Northern Italy. *International Orthopaedics*, 44(12):2499–2504, 2020.

[7] L. Androutsou, M. Kokkinos, D. Latsou, and M. Geitona. Assessing the Efficiency and Productivity of the Hospital Clinics on the Island of Rhodes during the COVID-19 Pandemic. *Int J Environ Res Public Health*, 19(23), 2022.

[8] M. Arafa, S. Nesar, H. Abu-Jabeh, M. O. R. Jayme, and Y. Kalairajah. COVID-19 pandemic and hip fractures: impact and lessons learned. *Bone and Joint Open*, 1(9):530–540, 2020.

[9] A. R. Arantes and V. Vieira. Did Ambulatory Surgical Units succeed in adapting to COVID-19 pandemic? The experience of a Portuguese Hospital with Ambulatory Surgery. *Ambulatory Surgery*, 27(2):39–43, 2021.

[10] R. A. Arisz, P. Meijer, N. C. V. Péquériaux, S. J. van de Leur, M. V. Lukens, M. P. M. de Maat, and M. J. Hollestelle. Impact of COVID-19 pandemic on the quality of test output in haemostasis laboratories. *International Journal of Laboratory Hematology*, 44(2):407–413, 2022.

[11] C. C. Bartenschlager, S. Temizel, A. Ebigbo, V. Gruenherz, P. Gastmeier, H. Messmann, J. O. Brunner, and C. Römmele. A Simulation-Based Cost-Effectiveness Analysis of Severe Acute Respiratory Syndrome Coronavirus 2 Infection Prevention Strategies for Visitors of Healthcare Institutions. *Value in Health*, 25(11):1846–1852, 2022.

[12] A. Belfiore, G. Centonze, P. Maisonneuve, C. Riva, D. Morelli, A. Mangogna, G. Sabella, G. Pruneri, and M. Milione. Covid-19 pandemic: Huge stress test for health system could be a great opportunity to update the workflow in a modern surgical pathology. *Cancers*, 13(13), 2021.

[13] R. A. Blauenfeldt, J. N. Hedegaard, C. Kruuse, D. Gaist, T. Wienecke, B. Modrau, D. Damgaard, S. P. Johnsen, G. Andersen, and C. Z. Simonsen. Quality in stroke care during the early phases of the COVID-19 pandemic: A nationwide study. *European Stroke Journal*, 8(1):268–274, 2023.

[14] V. Brunetti, A. Broccolini, P. Caliandro, R. Di Iorio, M. Monforte, R. Morosetti, C. Piano, F. Pilato, S. Bellavia, J. Marotta, I. Scala, A. Pedicelli, M. A. Pennisi, A. Caricato, C. Roberti, M. C. Altavista, A. Valenza, M. Distefano, E. Cecconi, M. Fanella, S. Roncacci, M. Tasillo, P. Calabresi, G. Frisullo, and G. D. Marca. Effect of the COVID-19 pandemic and the lockdown measures on the local stroke network. *Neurological Sciences*, 42(4):1237–1245, 2021.

[15] C. W. Böing, N. J. Froböse, F. Schaumburg, and S. Kampmeier. Impact of the COVID-19 Pandemic on the Management of Staphylococcus aureus Bloodstream Infections in a Tertiary Care Hospital. *Pathogens*, 12(4), 2023.

[16] P. Caldas and M. Varela. The Portuguese Public Hospitals Performance Evolution before and during the SARS-CoV-2 Pandemic (2017-2022). *SUSTAINABILITY*, 15(15), 2023.

[17] J. M. Casas-Rojo, P. S. Ventura, J. M. Antón Santos, A. O. de Latierro, J. C. Arévalo-Lorido, M. Mauri, M. Rubio-Rivas, R. González-Vega, V. Giner-Galvañ, B. Otero Perpiñá, E. Fonseca-Aizpuru, A. Muiño, E. Del Corral-Beamonte, R. Gómez-Huelgas, F. Arnalich-Fernández, M. Llorente Barrio, A. Sancha-Lloret, I. Rábago Lorite, J. Loureiro-Amigo, S. Pintos-Martínez, E. García-Sardón, A. Montaño-Martínez, M. G. Rojano-Rivero, J. M. Ramos-Rincón, and A. López-Escobar. Improving prediction of COVID-19 mortality using machine learning in the Spanish SEMI-COVID-19 registry. *Internal and emergency medicine*, 18(6):1711–1722, 2023.

[18] A. Chaddad and C. Tanougast. CNN Approach for Predicting Survival Outcome of Patients With COVID-19. *IEEE INTERNET OF THINGS JOURNAL*, 10(15):13742–13753, 2023.

[19] J. Correia, A. Ponte, C. Gomes, E. Afecto, M. M. Estevinho, A. Rodrigues, R. Pinho, and J. Carvalho. Readjustment of Capsule Endoscopy Protocols to the COVID-19 Pandemic in a Portuguese Tertiary Center. *Turkish Journal of Gastroenterology*, 33(7):570–575, 2022.

[20] G. Crozier-Shaw, A. J. Hughes, B. Conlon, E. Sheehan, and K. Merghani. Hip fracture care during Covid-19: a regional trauma centre's experience. *Irish Journal of Medical Science*, 190(4):1275–1280, 2021.

[21] S. J. Dauncey, P. A. Kelly, D. Baykov, A. C. Skeldon, and M. B. Whyte. Rhythmicity of patient flow in an acute medical unit: relationship to hospital occupancy, 7-day working and the effect of COVID-19. *Qjm*, 114(11):773–779, 2022.

[22] J. M. De Abreu, I. Cabo, M. I. Borges, A. Quiterio, T. Nunes, F. Matos, F. Vale, and J. Figueiredo. The COVID-19 Impact on Oral Healthcare Demand and Performance: The Experience of a Clinical and Academic Centre in Portugal (EU). *Acta Medica Portuguesa*, 36(1):15–24, 2023. Place: Portugal.

[23] L. Domingo, M. Comas, A. Jansana, J. Louro, H. Tizón-Marcos, M. L. Cos, J. Roquer, J. J. Chillarón, I. Cirera, S. Pascual-Guàrdia, M. Sala, and X. Castells. Impact of COVID-19 on Hospital Admissions and Healthcare Quality Indicators in Non-COVID Patients: A Retrospective Study of the First COVID-19 Year in a University Hospital in Spain. *Journal of Clinical Medicine*, 11(7), 2022.

[24] V. D'Ovidio, C. Lucidi, G. Bruno, D. Lisi, L. Miglioresi, and M. E. Bazuro. Impact of COVID-19 Pandemic on Colorectal Cancer Screening Program. *CLINICAL COLORECTAL CANCER*, 20(1):E5–E11, 2021.

[25] R. Doyle, S. Bate, J. Devenney, S. Agwaonye, M. Hastings, J. Wych, S. Archbold, and D. H. Vasant. Evaluating the impact of an enhanced triage process on the performance and diagnostic yield of oesophageal physiology studies post COVID-19. *BMJ Open Gastroenterology*, 8(1), 2021.

[26] D. Duarte, C. Walshaw, and N. Ramesh. A comparison of time-series predictions for healthcare emergency department indicators and the impact of COVID-19. *Applied Sciences (Switzerland)*, 11(8), 2021.

[27] J. Dupraz, E. Zuercher, and I. Peytremann-Bridevaux. Impact of the COVID-19 pandemic on the quality of diabetes care: Insights from longitudinal data in Switzerland. *Primary Care Diabetes*, 17(4):408–410, 2023.

[28] M. A. Elleuch, A. Ben Hassena, M. Abdelhedi, and F. S. Pinto. Real-time prediction of COVID-19 patients health situations using Artificial Neural Networks and Fuzzy Interval Mathematical modeling. *APPLIED SOFT COMPUTING*, 110, 2021.

[29] S. Esmaily, C. C. Yau, D. Dwarakanath, J. Hancock, and V. Mitra. Impact of COVID-19 pandemic on key performance indicators in pancreatobiliary endoscopy: Prioritise, minimise risk, keep scoping and training. *Frontline Gastroenterology*, 12(4):288–292, 2021.

[30] Ann-Charlotte Falk, Carolin Nymark, Katarina E. Göransson, and Ann-Christin von Vogelsang. Missed nursing care in the critical care unit, before and during the COVID-19 pandemic: A comparative cross-sectional study. *Intensive & Critical Care Nursing*, 72:N.PAG–N.PAG, 2022. Place: Philadelphia, Pennsylvania.

[31] C. Fazekas, M. Zieser, B. Hanfstingl, J. Saretzki, E. Kunschitz, L. Zieser-Stelzhammer, D. Linder, and F. Matzer. Physician resilience and perceived quality of care among medical doctors with training in psychosomatic medicine during the COVID-19 pandemic: a quantitative and qualitative analysis. *BMC Health Services Research*, 24(1), 2024.

[32] O. Fersia, S. Bryant, R. Nicholson, K. McMeeken, C. Brown, B. Donaldson, A. Jardine, V. Grierson, V. Whalen, and A. MacKay. The impact of the COVID-19 pandemic on cardiology services. *Open Heart*, 7(2), 2020.

[33] D. Fluck, C. H. Fry, S. Rankin, A. Lewis, J. Robin, J. Rees, J. Finch, Y. Jones, G. Jones, J. Tudose, L. Taylor, and T. S. Han. Does the length of stay in hospital affect healthcare outcomes of patients without COVID-19 who were admitted during the pandemic? A retrospective monocentric study. *Internal and emergency medicine*, 17(5):1385–1393, 2022.

[34] G. Frisullo, V. Brunetti, R. Di Iorio, A. Broccolini, P. Caliandro, M. Monforte, R. Morosetti, C. Piano, F. Pilato, P. Calabresi, and G. Della Marca. Effect of lockdown on the management of ischemic stroke: an Italian experience from a COVID hospital. *Neurol Sci*, 41(9):2309–2313, 2020.

[35] M. J. Ghassemi-Rad, C. Dennehy, N. Lyons, M. T. Henry, M. P. Kennedy, ÉJ O'Reilly, and R. M. Connolly. The impact of the COVID-19 pandemic on the performance of the Rapid Access Lung Cancer Clinic. *Irish Journal of Medical Science*, 2024.

[36] H. Ghayoomi, E. Miller-Hooks, M. Tariverdi, J. Shortle, and T. D. Kirsch. Maximizing hospital capacity to serve pandemic patient surge in hot spots via queueing theory and microsimulation. *IISE Transactions on Healthcare Systems Engineering*, 13(4):314–332, 2023.

[37] Adrian Gheorghe, Camille Maringe, James Spice, Arnie Purushotham, Kalipso Chalkidou, Bernard Rachet, Richard Sullivan, and Ajay Aggarwal. Economic impact of avoidable cancer deaths caused by diagnostic delay during the COVID-19 pandemic: A national population-based modelling study in England, UK. *European Journal of Cancer*, 152:233–242, 2021.

[38] P. D. Giorgi, F. Villa, E. Gallazzi, A. Debernardi, G. R. Schirò, F. M. Crisà, G. Talamonti, and G. D'Aliberti. The management of emergency spinal surgery during the COVID-19 pandemic in Italy. *Bone Joint J*, 102-b(6):671–676, 2020.

[39] A. Gobbi, D. Manerba, R. Mansini, and R. Zanotti. Hybridizing adaptive large neighborhood search with kernel search: a new solution approach for the nurse routing problem with incompatible services and minimum demand. *INTERNATIONAL TRANSACTIONS IN OPERATIONAL RESEARCH*, 30(1):8–38, 2023.

[40] A. González-Cebrián, J. Borràs-Ferrís, J. P. Ordovás-Baines, M. Hermenegildo-Caudevilla, M. Climente-Marti, S. Tarazona, R. Vitale, D. Palací-López, J. F. Sierra-Sánchez, J. S. de la Fuente, and A. Ferrer. Machine-learning-derived predictive score for early estimation of COVID-19 mortality risk in hospitalized patients. *PLoS One*, 17(9 September), 2022.

[41] B. S. Gonçalves, E. Vieira, R. M. Lima, and J. Dinis-Carvalho. A systematized approach for reduction of medical appointment waiting list. *Production*, 32, 2022.

[42] P. Gonçalves, P. Ferrari, L. Crivelli, and E. Albanese. Model-informed health system reorganization during emergencies. *PRODUCTION AND OPERATIONS MANAGEMENT*, 32(5):1323–1344, 2023.

[43] A. Graf, C. H. Koh, G. Caldwell, J. Grieve, M. Tan, J. Hassan, K. Bakaya, H. J. Marcus, and S. E. Baldeweg. Quality in Clinical Consultations: A Cross-Sectional Study. *Clinics and Practice*, 12(4):545–556, 2022.

[44] S. Griewing, N. Gremke, J. Kreutz, B. Schieffer, L. Timmermann, and B. Markus. Chronological Development of Cardiovascular Disease in Times of COVID-19: A Retrospective Analysis of Hospitalized Diseases of the Circulatory System and COVID-19 Patients of a German University Hospital. *Journal of Cardiovascular Development and Disease*, 9(10), 2022.

[45] S. Griewing, U. Wagner, M. Lingenfelder, S. Heinis, B. Schieffer, and B. Markus. Impact of the COVID-19 Pandemic on Delivery of Gynecology and Obstetrics Services at a Maximum Care University Hospital in Germany. *Geburtshilfe und Frauenheilkunde*, 82(4):427–440, 2022.

[46] F. Grimm, A. Johansen, H. Knight, R. Brine, and S. R. Deeny. Indirect effect of the COVID-19 pandemic on hospital mortality in patients with hip fracture: A competing risk survival analysis using linked administrative data. *BMJ Quality and Safety*, 32(5):264–273, 2023.

[47] S. Gruttadauria. Preliminary Analysis of the Impact of the Coronavirus Disease 2019 Outbreak on Italian Liver Transplant Programs. *Liver Transplantation*, 26(7):941–944, 2020.

[48] M. B. B. Hansen, M. Groenvold, M. Adsersen, H. Jensen, E. H. Ibfelt, M. A. Petersen, M. A. Neergaard, H. Møller, and T. B. Olesen. Palliative care need screening and specialised referrals fell during the COVID-19 pandemic: a nationwide register-based study. *BMJ Supportive and Palliative Care*, 14(e1):E1380–E1387, 2024.

[49] C. O. Henriques and M. C. Gouveia. Assessing the impact of COVID-19 on the efficiency of Portuguese state-owned enterprise hospitals. *SOCIO-ECONOMIC PLANNING SCIENCES*, 84, 2022.

[50] J. Horan, J. C. Duddy, B. Gilmartin, M. Amoo, D. Nolan, P. Corr, M. B. Husien, and C. Bolger. Neurotrauma admissions and COVID-19: a National Centre experience. *Irish Journal of Medical Science*, 191(4):1871–1876, 2022.

[51] B. Jamshidi, S. J. Zargaran, H. Bekrizadeh, M. Rezaei, and F. Najafi. Comparing length of hospital stay during COVID-19 pandemic in the USA, Italy and Germany. *International Journal for Quality in Health Care*, 33(1), 2021.

[52] S. Jidkova, S. Hoeck, E. Kellen, S. le Cessie, and M. C. Goossens. Flemish population-based cancer screening programs: impact of COVID-19 related shutdown on short-term key performance indicators. *BMC Cancer*, 22(1):183, 2022.

[53] J. Joseph, R. A. Kotronias, T. Estrin-Serlui, T. J. Cahill, R. K. Kharbanda, J. D. Newton, C. Grebenik, S. Dawkins, and A. P. Banning. Safety and Operational Efficiency of Restructuring and Redeploying a Transcatheter Aortic Valve Replacement Service During the COVID-19 Pandemic: The Oxford Experience. *Cardiovascular Revascularization Medicine*, 31:26–31, 2021.

[54] Q. Kong, D. Riedewald, and M. Askari. Factors affecting portal usage among chronically ill patients during the COVID-19 pandemic in the Netherlands: Cross-sectional study. *JMIR HUMAN FACTORS*, 8(3), 2021.

[55] T. Kuosmanen, Y. Tan, and S. Dai. Performance analysis of English hospitals during the first and second waves of the coronavirus pandemic. *Health Care Management Science*, 26(3):447–460, 2023.

[56] L. L. Liang, C. H. Tseng, H. J. Ho, and C. Y. Wu. Covid-19 mortality is negatively associated with test number and government effectiveness. *Scientific reports*, 10(1), 2020.

[57] G. Lo Moro, N. Marengo, A. Mara, J. R. Paño Pardo, S. Hernandez, E. Fusté, M. Pujol, C. M. Zotti, E. Limón, and C. Vicentini. Antimicrobial stewardship programs in acute-care hospitals: A multicenter assessment of structure, process, and outcome indicators in Italy and Spain. *Journal of Infection and Public Health*, 17(7), 2024.

[58] A. Losurdo, A. V. E. Lisa, M. Tomatis, A. Ponti, S. Montemezzi, E. Bonzano, and L. Fortunato. Highly specialized Breast Centers did not experience delay of care during COVID-19 pandemic in Italy: the Senonetwork experience. *Breast Cancer Res Treat*, 196(1):87–95, 2022.

[59] S. Luthra, G. Hunduma, M. Navaratnarajah, P. G. Malvindi, N. Goddard, S. Miskolczi, and T. Velissaris. Impact of COVID-19 on operation room utilization efficiency and cardiac surgery care pathway: single centre experience. *JOURNAL OF HOSPITAL MANAGEMENT AND HEALTH POLICY*, 7, 2023.

[60] A. López-Cheda, M. A. Jácome, R. Cao, and P. M. De Salazar. Estimating lengths-of-stay of hospitalised COVID-19 patients using a non-parametric model: a case study in Galicia (Spain). *Epidemiology and Infection*, 149, 2021.

[61] Y. Manla, F. A. Badarin, N. Bader, T. Lee-St John, M. R. Mehra, and F. Bader. Worldwide and Country-Specific Impact of the COVID-19 Pandemic on Heart Transplantation Volumes: A Longitudinal Analysis of 2020 and 2021. *Curr Probl Cardiol*, 48(11):101870, 2023.

[62] C. Mattiuzzi, G. Lippi, and B. M. Henry. Healthcare indicators associated with COVID-19 death rates in the European Union. *Public Health*, 193:41–42, 2021.

[63] E. Maury, M. O. Boldi, G. Greub, V. Chavez, K. Jaton, and O. Opota. An Automated Dashboard to Improve Laboratory COVID-19 Diagnostics Management. *Frontiers in Digital Health*, 3, 2021.

[64] S. Meakin and S. Funk. Quantifying the impact of hospital catchment area definitions on hospital admissions forecasts: COVID-19 in England, September 2020-April 2021. *BMC Medicine*, 22(1), 2024.

[65] G. J. Melman, A. K. Parlikad, and E. A. B. Cameron. Balancing scarce hospital resources during the COVID-19 pandemic using discrete-event simulation. *Health Care Management Science*, 24(2):356–374, 2021.

[66] J. K. Mortensen, R. A. Blauenfeldt, J. N. Hedegaard, C. M. Wejse, S. P. Johnsen, G. Andersen, and C. Z. Simonsen. Prevalence and impact of SARS-CoV-2 infection among patients with acute ischaemic stroke: a nationwide register-based cohort study in Denmark. *BMJ Open*, 14(3), 2024.

[67] C. P. Mutch, D. A. Ross, A. Bularga, R. N. R. Cave, M. E. Chase-Topping, A. Anand, N. L. Mills, O. Koch, C. L. Mackintosh, and M. R. Perry. Performance status: A key factor in predicting mortality in the first wave of COVID-19 in South-East Scotland. *Journal of the Royal College of Physicians of Edinburgh*, 52(3):204–212, 2022.

[68] G. Nattino, M. M. Paganuzzi, G. I. Ghilardi, G. Costantino, C. Rossi, F. Cortellaro, R. Cosentini, S. Paglia, M. Migliori, and G. Bertolini. Strategies to convert hospital beds for COVID-19 patients to minimize emergency department overcrowding. *Health Services Management Research*, 2023.

[69] A. M. Nunes and D. F. D. Ferreira. Evaluating Portuguese Public Hospitals Performance: Any Difference before and during COVID-19? *SUSTAINABILITY*, 15(1), 2023.

[70] L. Palazzo and R. Ievoli. Detecting Regional Differences in Italian Health Services during Five COVID-19 Waves. *STATS*, 6(2):506–518, 2023.

[71] C. Park, K. Sugand, A. Aframian, C. Morgan, N. Pakroo, C. Gibbons, M. Fertleman, D. Nathwani, R. Bhattacharya, and K. M. Sarraf. Impact of COVID-19 pandemic on hip fractures: the central London experience COVID-related urgent geriatric hip trauma (COUGH) study COVERT (COVid Emergency-Related Trauma and orthopaedics) collaborative. *Irish Journal of Medical Science*, 191(3):1005–1012, 2022.

[72] A. B. Pedersen, N. Risbo, B. L. Viberg, H. Palm, and N. Dieter Röck. Incidence, Quality of Care, and Mortality in Hip Fracture Patients in the Course of the COVID Pandemic: A Population-Based Cohort Study. *Journals of Gerontology - Series A Biological Sciences and Medical Sciences*, 79(4), 2024.

[73] N. Penel, C. Cantarel, C. Chemin-Airiau, F. Ducimetiere, F. Gouin, F. Le Loarer, M. Toulmonde, S. Piperno-Neumann, C. Bellera, C. Honore, J. Y. Blay, and S. Mathoulin-Pelissier. Impact of the coronavirus disease 2019 pandemic on sarcoma management in France: a 2019 and 2020 comparison. *Therapeutic Advances in Medical Oncology*, 15, 2023.

[74] A. M. Ponsiglione, M. Romano, and F. Amato. A Finite-State Machine Approach to Study Patients Dropout from Medical Examinations. *6th International Forum on Research and Technology for Society and Industry, RTSI 2021 - Proceedings*, pages 289–294, 2021.

[75] T. Quibel, N. Winer, L. Bussieres, C. Vayssiere, P. Deruelle, M. Defrance, P. Rozenberg, J. Bouyer, N. Dupuis, B. Renaudin, L. Dugave, N. Banaszkiewicz, C. Garabedian, and Y. Ville. Impact of COVID-19-Related Lockdown on Delivery and Perinatal Outcomes: A Retrospective Cohort Study. *Journal of Clinical Medicine*, 11(3), 2022.

[76] L. Quinn, P. Bird, and R. Lilford. Effect of cancer waiting time standards in the English National Health Service: a threshold analysis. *BMC Health Services Research*, 24(1), 2024.

[77] A. Ramírez Calazans, R. M. Paredes Esteban, O. B. Grijalva Estrada, and M. R. Ibarra Rodríguez. Assessment of quality indicators in pediatric major outpatient surgery. Influence of the COVID-19 pandemic. *Cirugia pediatrica : organo oficial de la Sociedad Espanola de Cirugia Pediatrica*, 36(1):17–21, 2023.

[78] N. Reidy, H. Coetzee, C. Roche, E. Brazil, L. O'Sullivan, D. Brady, and M. Lynch. SARS-CoV-2 Testing and Patient Waiting Times in the Emergency Department. *Irish medical journal*, 115(7):633, 2022.

[79] K. H. Scholz, B. Lengenfelder, C. Thilo, A. Jeron, S. Stefanow, U. Janssens, J. Bauersachs, P. C. Schulze, K. D. Winter, J. Schröder, J. vom Dahl, N. von Beckerath, K. Seidl, T. Friede, and T. Meyer. Impact of COVID-19 outbreak on regional STEMI care in Germany. *Clinical Research in Cardiology*, 109(12):1511–1521, 2020.

[80] A. Sedghi, T. Siepmann, L. P. Pallesen, H. Reichmann, V. Puetz, J. Barlinn, and K. Barlinn. Delayed comprehensive stroke unit care attributable to the evolution of infection protection measures across two consecutive waves of the covid-19 pandemic. *Life*, 11(7), 2021.

[81] E. F. Sánchez-Ubeda, P. Sánchez-Martín, M. Torrego-Ellacuría, A. Del Rey-Mejías, M. F. Morales-Contreras, and J. L. Puerta. Flexibility and Bed Margins of the Community of Madrid's Hospitals during the First Wave of the SARS-CoV-2 Pandemic. *International Journal of Environmental Research and Public Health*, 18(7), 2021.

[82] G. Terning, E. C. Brun, and I. El-Thalji. Modeling Patient Flow in an Emergency Department under COVID-19 Pandemic Conditions: A Hybrid Modeling Approach. *Healthcare (Switzerland)*, 10(5), 2022.

[83] D. Tricarico, M. Calandri, M. Barba, C. Piatti, C. Geninatti, D. Basile, M. Gatti, M. Melis, and A. Veltri. Convolutional Neural Network-Based Automatic Analysis of Chest Radiographs for the Detection of COVID-19 Pneumonia: A Prioritizing Tool in the Emergency Department, Phase I Study and Preliminary "Real Life" Results. *Diagnostics*, 12(3), 2022.

[84] T. A. Trunfio and G. Improta. Flow Analysis of Mastectomy Patients Using Length of Stay: A Single-Center Study. *BioMedInformatics*, 4(3):1725–1744, 2024.

[85] Y. Tung-Chen, A. Gil-Rodrigo, A. Algora-Martín, R. Llamas-Fuentes, P. Rodríguez-Fuertes, R. Marín-Baselga, B. Alonso-Martínez, E. S. Rodríguez, P. L. Soriano, and J. M. Ramos-Rincón. The lung ultrasound "Rule of 7" in the prognosis of COVID-19 patients: Results from a prospective multicentric study. *Medicina Clinica*, 159(1):19–26, 2022.

[86] I. Vagliano, S. Brinkman, A. Abu-Hanna, M. S. Arbous, D. A. Dongelmans, P. W. G. Elbers, D. W. de Lange, M. van der Schaar, N. F. de Keizer, and M. C. Schut. Can we reliably automate clinical prognostic modelling? A retrospective cohort study for ICU triage prediction of in-hospital mortality of COVID-19 patients in the Netherlands. *International Journal of Medical Informatics*, 160:N.PAG–N.PAG, 2022. Place: New York, New York.

[87] Davide Valsecchi, Luca Porcu, Abdelrahman Khater, Rosa Alessia Battista, Leone Giordano, Stefano Cascinu, Andrea Assanelli, Chiara Lazzari, Vanesa Gregorc, and Aurora Mirabile. Emergency Department Visits among Cancer Patients during SARS-CoV-2 Pandemic. *Cancers*, 15(4):1240, 2023.

[88] P. van Dam, M. Tomatis, A. Ponti, L. Marotti, C. Aristei, L. Biganzoli, M. J. Cardoso, K. L. Cheung, G. Curigliano, J. De Vries, D. Santini, F. Sardanelli, and I. T. Rubio. The impact of the SARS-COV-2 pandemic on the quality of breast cancer care in EUSOMA-certified breast centres. *Eur J Cancer*, 177:72–79, 2022.

[89] S. Vanni, P. Bartalucci, U. Gargano, A. Coppa, G. Giannasi, P. Nazerian, B. Tonietti, R. Vannini, M. Lanigra, F. Daviddi, A. Baldini, S. Grifoni, and S. Magazzini. The presentations/physician ratio predicts door-to-physician time but not global length of stay in the emergency department: an Italian multicenter study during the SARS-CoV-2 pandemic. *Internal and emergency medicine*, 17(3):829–837, 2022.

[90] J. L. Waterman, U. Jayaraju, J. K. Nadimi, and D. Morgan. Impact of COVID-19 on Key Performance Indicators of the National Hip Fracture Database and the Management of Hip Fracture Patients. *CUREUS JOURNAL OF MEDICAL SCIENCE*, 13(12), 2021.

[91] P. E. Wikman-Jorgensen, A. Ruiz, V. Giner-Galvan, J. Llenas-Garcia, J. M. Segui-Ripoll, J. M. Salinas Serrano, E. Borrajo, J. M. Ibarra Sanchez, J. P. Garcia-Sabater, and J. A. Marin-Garcia. Hospitalization forecast to inform COVID-19 pandemic planning and resource allocation using mathematical models. *medRxiv*, 2022. Place: United States.

[92] R. M. Wood. Modelling the impact of COVID-19 on elective waiting times. *JOURNAL OF SIMULATION*, 16(1):101–109, 2022.

[93] R. M. Wood, C. J. McWilliams, M. J. Thomas, C. P. Bourdeaux, and C. Vasilakis. COVID-19 scenario modelling for the mitigation of capacity-dependent deaths in intensive care. *Health Care Management Science*, 23(3):315–324, 2020.

[94] E. Woodland and M. Carroll. Improving ICSI success rates following root cause analysis and use of system behaviour charts: the devil is in the detail! *BMJ Open Quality*, 11(4), 2022.
