# Supplementary material for: Performance indicators for evaluating and optimising hospital services during COVID-19: a scoping review
Source: BMJ Open. 2026 Sep 28;16(9):e122362. doi: 10.1136/bmjopen-2026-122362 (PMC13630113; doi:10.1136/bmjopen-2026-122362)
Supplement: Supplementary file 3 [file bmjopen-2026-122362.reviewer_comments.pdf]

PEER REVIEW HISTORY

BMJ Open publishes all reviews undertaken for accepted manuscripts. Reviewers are asked to complete a checklist review form and are provided with free text boxes to elaborate on their assessment. These free text comments are reproduced below.

ARTICLE DETAILS

Title (Provisional)

Performance indicators for evaluating and optimising hospital services during COVID-19: a scoping review

Authors

Dankers, Anna; Yildirim, Dilek; Dijkstra, Sander; Ngongalah, Lem; Connolly, Máire A.; Duggan, Jim; Zander, Anne; van Loenen, Tessa; Boucherie, Richard J.; Timen, Aura

---

VERSION 1 - REVIEW

**Reviewer**	**1**
**Name**	**Wiblishauser, Michael**
**Affiliation**	**University of Houston Victoria**
**Date**	**07-Jul-2026**
**COI**	**None**

A good and extensive scoping review....

One question: Did the studies cover the composition of the hospitals (generalists vs. specialists)?

**Reviewer**	**2**
**Name**	**Malambo, Warren**
**Affiliation**	**University of Zambia**
**Date**	**05-Aug-2026**
**COI**	**None**

---

1. Methods section is missing protocol registration information. Per PRISMA-ScR item number 5, authors should indicate whether a review protocol exists; state if and where it can be accessed (e.g., a Web address); and if available, provide registration information, including the registration number. This has not been done.

2. We'd recommend that authors show search results per each of the databases searched in the PRISMA diagram that yielded a total off 3300 records. This is to ensure that search strategy for at least one database can be validated.

3. Authors may wise to consider whether other scoping-review limitations (page 22), including exclusion of gray literature, no formal critical appraisal, possible missed studies because terminology for KPIs varies, and limitations of inductive categorization apply to their study.

4. Conclusion (page22): The conclusion matches the scoping-review aim. However, I'd suggest that authors temper action-oriented language slightly and avoid implying effectiveness or causal priority among KPI sets, because the review mapped indicators rather than evaluating their predictive or operational performance. For example, avoid using directive language (... studies should specify...; ...overview can

support...;). Use such terms as "may support", "could support", etc.

VERSION 1 - AUTHOR RESPONSE

Dr. Michael Wiblishauser:

- *"Did the studies cover the composition of the hospitals (generalists vs. specialists)?"*

  Thank you for taking time to review our scoping review. As part of the data extraction process, we recorded the hospital type for each included article. Hospital types were categorized as general hospital, academic hospital, specialty hospital, unknown type, or combinations of these categories. This information is described at the article level in the Results section on page 6 of the manuscript. If an overview at the KPI level is desired, this information is available in the full KPI dataset and can be provided upon request.

Dr. Warren Malambo:

- *"Methods section is missing protocol registration information. Per PRISMA-ScR item number 5, authors should indicate whether a review protocol exists; state if and where it can be accessed (e.g., a Web address); and if available, provide registration information, including the registration number. This has not been done."*

  Thank you for pointing this out. We agree that the protocol and registration information was insufficiently reported in the original manuscript. In accordance with PRISMA-ScR Item 5, we have now added a statement to the Methods section clarifying whether a review protocol existed and whether it was preregistered. An a priori methodological plan, including the eligibility criteria and data items to be extracted, was developed before conducting the review; however, this was not prospectively registered or made publicly available. We have clarified this in the revised manuscript (section 2.1, page 4).

- “*We'd recommend that authors show search results per each of the databases searched in the PRISMA diagram that yielded a total off 3300 records. This is to ensure that search strategy for at least one database can be validated.”*

  We have added the number of records identified per database to clarify the contribution of each database to the search results. This information has been added to the first paragraph of the Results section on page 6.
- *“Authors may wise to consider whether other scoping-review limitations (page 22), including exclusion of gray literature, no formal critical appraisal, possible missed studies because terminology for KPIs varies, and limitations of inductive categorization apply to their study.”*

  We have expanded the Strengths and Limitations section (page 18). In the revised manuscript, we also mention limitations regarding grey literature, challenges related to searching for KPI-related terminology, subjectivity in the inductive categorisation, and the absence of formal quality appraisal.
- *“The conclusion matches the scoping-review aim. However, I'd suggest that authors temper action-oriented language slightly and avoid implying effectiveness or causal priority among KPI sets, because the review mapped indicators rather than evaluating their predictive or operational performance. For example, avoid using directive language (... studies should specify...; ...overview can support...;). Use such terms as "may support", "could support", etc.”*

  Thank you for this comment and your suggestions, they are very useful. We agree that the language used Discussion is slightly directive. We have revised the wording throughout this section, and the Abstract has been adjusted accordingly.

We hope that the revised manuscript satisfactorily addresses the comments and recommendations provided. Thank you again for the constructive feedback and the opportunity to revise our manuscript.
