# Supplementary material for: Performance indicators for evaluating and optimising hospital services during COVID-19: a scoping review
Source: BMJ Open. 2026 Sep 28;16(9):e122362. doi: 10.1136/bmjopen-2026-122362 (PMC13630113; doi:10.1136/bmjopen-2026-122362)
Supplement: Supplementary file 4 [file bmjopen-16-9-s001.docx]

Appendix A: search string

**A. PUBMED**

**Concept 1: Pandemic/Health crises**

"Disease Outbreaks"[Mesh] OR "COVID-19"[Mesh] OR "SARS-CoV-2"[Mesh] OR Outbreak*[tiab] OR Epidemic*[tiab] OR Pandemic*[tiab] OR "Health Cris*"[tiab] OR "Health Emergenc*"[tiab] OR "COVID-19"[tiab] OR "COVID 19"[tiab]

**Concept 2: Performance**

"Efficiency, Organizational"[Mesh] OR "Health performance"[tiab:~2] OR "Health performances"[tiab:~2] OR "Healthcare performance"[tiab:~2] OR "Healthcare performances"[tiab:~2] OR "Logistic Performance"[tiab:~2] OR "Logistic performances"[tiab:~2] OR "Logistical Performance"[tiab:~2] OR "Logistical performances"[tiab:~2] OR "Key Performance"[tiab:~2] OR "Key performances"[tiab:~2] OR "Hospital Performance"[tiab:~2] OR "Hospital performances"[tiab:~2] OR "Medical performance"[tiab:~2] OR "Medical performances"[tiab:~2] OR "Performance Indicator*"[tiab] OR "Performance Metric*"[tiab] OR "Performance Measure*"[tiab] OR "KPI"[tiab] OR "Quality Indicator*"[tiab] OR "Operational efficiency"[tiab] OR "Operation* research"[tiab] OR "Operation* management"[tiab] OR "Organizational Efficiency"[tiab]

**Concept 3: Hospital care**

"Hospitals"[Mesh] OR "Hospital Units"[Mesh] OR "Hospital Departments"[Mesh] OR "Health Services"[Mesh] OR "Hospital*"[tiab] OR "Care service*"[tiab] OR "Care center*"[tiab] OR "Clinic"[tiab] OR "Clinics"[tiab] OR "Patient care"[tiab] OR "Emergency Department"[tiab] OR “ED”[tiab] OR "Intensive Care Unit*"[tiab] OR "ICU"[tiab] OR "Cardiolog*"[tiab] OR "Oncolog*"[tiab] OR "Neurolog*"[tiab] OR "Pediatric*"[tiab] OR "OB/GYN"[tiab] OR "Surger*"[tiab] OR "Surgeon*"[tiab] OR "Orthopedic*"[tiab] OR "Geriatric*"[tiab] OR "Radiolog*"[tiab] OR "Pulmonolog*"[tiab] OR "Nephrolog*"[tiab] OR "Gastroenterolog*"[tiab] OR "Dermatolog*"[tiab] OR "Hematolog*"[tiab] OR "Endocrinolog*"[tiab] OR "Anesthesiolog*"[tiab] OR "Trauma Center*"[tiab] OR "Transplant Center*"[tiab] OR "Urgent Care"[tiab] OR "Cancer Center*"[tiab] OR "Ward*"[tiab] OR "Hospital Unit*"[tiab] OR "Hospital Department*"[tiab] OR "Health Service*"[tiab]

**B. CINAHL:**

**Concept 1: Pandemic/Health crises**

((MH "COVID-19+") OR (MH "Disease Outbreaks+") or (MH "SARS-CoV-2+") OR TI(Outbreak* OR Epidemic* OR Pandemic* OR "Health Cris*" OR "Health Emergenc*" OR "COVID-19" OR "COVID 19") OR AB(Outbreak* OR Epidemic* OR Pandemic* OR "Health Cris*" OR "Health Emergenc*" OR "COVID-19" OR "COVID 19"))

**Concept 2: Performance**
((MH "Organizational Efficiency+") OR TI(((Health* OR Logistic* OR Key OR Hospital OR Medical) N2 Performance*)  OR "Performance Indicator*" OR "Performance Metric*" OR "Performance Measure*" OR "KPI" OR "Quality Indicator*" OR "Operational efficiency" OR "Operation* research" OR "Operation* management") OR AB(((Health* OR Logistic* OR Key OR Hospital OR Medical) N2 Performance*)  OR "Performance Indicator*" OR "Performance Metric*" OR "Performance Measure*" OR "KPI" OR "Quality Indicator*" OR "Operational efficiency" OR "Operation* research" OR "Operation* management"))

**Concept 3: Hospital care**
((MH "Hospitals+") OR (MH "Hospital units+") OR (MH "Health services+") OR (MH "Health facility departments+") OR **TI**("Hospital" OR "Hospitals" OR "Hospital Unit*" OR "Health Service*" OR "Health Facility Department*" OR "Care service*" OR "Care center*" OR "Clinic" OR "Clinics" OR "Patient care" OR "Emergency Department" OR “ED” OR "Intensive Care Unit*" OR "ICU" OR "Cardiolog*" OR "Oncolog*" OR "Neurolog*" OR "Pediatric*" OR "OB/GYN" OR "Surger*" OR "Surgeon*" OR "Orthopedic*" OR "Geriatric*" OR "Radiolog*" OR "Pulmonolog*" OR "Nephrolog*" OR "Gastroenterolog*" OR "Dermatolog*" OR "Hematolog*" OR "Endocrinolog*" OR "Anesthesiolog*" OR "Trauma Center*" OR "Transplant Center*" OR "Urgent Care" OR "Cancer Center*" OR "Ward*") OR **AB**("Hospital" OR "Hospitals" OR "Care service*" OR "Care center*" OR "Clinic" OR "Clinics" OR "Patient care" OR "Emergency Department" OR “ED” OR "Intensive Care Unit*" OR "ICU" OR "Cardiolog*" OR "Oncolog*" OR "Neurolog*" OR "Pediatric*" OR "OB/GYN" OR "Surger*" OR "Surgeon*" OR "Orthopedic*" OR "Geriatric*" OR "Radiolog*" OR "Pulmonolog*" OR "Nephrolog*" OR "Gastroenterolog*" OR "Dermatolog*" OR "Hematolog*" OR "Endocrinolog*" OR "Anesthesiolog*" OR "Trauma Center*" OR "Transplant Center*" OR "Urgent Care" OR "Cancer Center*" OR "Ward*"))

**C. EMBASE:**

**Concept 1: Pandemic/Health crises**
exp epidemic/ OR exp pandemic/ OR exp coronavirus disease 2019/ OR (Outbreak* OR Epidemic* OR Pandemic* OR "Health Cris*" OR "Health Emergenc*" OR “COVID-19” OR “COVID 19” OR "coronavirus disease 2019").ti,ab,kf.

**Concept 2: Performance**
exp organizational efficiency/ OR (((Health* OR Logistic* OR Key OR Hospital OR Medical) adj3 Performance*)  OR "Performance Indicator*" OR "Performance Metric*" OR "Performance Measure*" OR "KPI" OR "Quality Indicator*" OR "Operational efficiency" OR "Operation* research" OR "Operation* management" OR "Organizational efficiency").ti,ab,kf.

**Concept 3: Hospital care**

exp hospital / OR exp hospital care/ OR exp health service/ OR ("Hospital" OR "Hospitals" OR "Hospital care" OR "Health service*" OR "Care service*" OR "Care center*" OR "Clinic" OR “Clinics” OR “Patient care” OR "Emergency Department" OR “ED” OR "Intensive Care Unit*" OR "ICU" OR "Cardiolog*" OR "Oncolog*" OR "Neurolog*" OR "Pediatric*" OR "OB/GYN" OR "Surger*" OR "Surgeon*" OR "Orthopedic*" OR "Geriatric*" OR "Radiolog*" OR "Pulmonolog*" OR "Nephrolog*" OR "Gastroenterolog*" OR "Dermatolog*" OR "Hematolog*" OR "Endocrinolog*" OR "Anesthesiolog*" OR "Trauma Center*" OR "Transplant Center*" OR "Urgent Care" OR "Cancer Center*" OR "Ward*").ti,ab,kf.

**D. SCOPUS and WOS:**

**Concept 1: Pandemic/Health crises**
Outbreak* OR Epidemic* OR Pandemic* OR "Health Cris*" OR "Health Emergenc*" OR “COVID-19” OR “COVID 19”

**Concept 2: Performance**
((Health* OR Logistic* OR Key OR Hospital OR Medical) NEAR/2 Performance*)  OR "Performance Indicator*" OR "Performance Metric*" OR "Performance Measure*" OR “KPI” OR  "Quality Indicator*" OR "Operational efficiency" OR “Operation* research” OR “Operation* management”

**Concept 3: Hospital care**
"Hospital" OR "Hospitals" OR "Care service*" OR "Care center*" OR "Clinic" OR "Clinics" OR "Patient care"

OR "Emergency Department" OR “ED” OR "Intensive Care Unit*" OR "ICU" OR "Cardiolog*" OR "Oncolog*" OR "Neurolog*" OR "Pediatric*" OR "OB/GYN" OR "Surger*" OR "Surgeon*" OR "Orthopedic*" OR "Geriatric*" OR "Radiolog*" OR "Pulmonolog*" OR "Nephrolog*" OR "Gastroenterolog*" OR "Dermatolog*" OR "Hematolog*" OR "Endocrinolog*" OR "Anesthesiolog*" OR "Trauma Center*" OR "Transplant Center*" OR "Urgent Care" OR "Cancer Center*" OR "Ward*"
